# HIV among immigrants living in high-income countries: a realist review of evidence to guide targeted approaches to behavioural HIV prevention

**DOI:** 10.1186/2046-4053-1-56

**Published:** 2012-11-20

**Authors:** Tadgh McMahon, Paul R Ward

**Affiliations:** 1 Multicultural HIV and Hepatitis Service, PO Box M139, MISSENDEN ROAD, Camperdown, NSW, 2050, Australia; 2 Discipline of Public Health, School of Medicine, Flinders University, GPO Box 2100, Flinders, SA, 5001, Australia

**Keywords:** HIV prevention, Immigrants, Realist review, Culturally appropriate, Behavioural interventions

## Abstract

**Background:**

Immigrants from developing and middle-income countries are an emerging priority in HIV prevention in high-income countries. This may be explained in part by accelerating international migration and population mobility. However, it may also be due to the vulnerabilities of immigrants including social exclusion along with socioeconomic, cultural and language barriers to HIV prevention. Contemporary thinking on effective HIV prevention stresses the need for targeted approaches that adapt HIV prevention interventions according to the cultural context and population being addressed. This review of evidence sought to generate insights into targeted approaches in this emerging area of HIV prevention.

**Methods:**

We undertook a realist review to answer the research question: ‘How are HIV prevention interventions in high-income countries adapted to suit immigrants’ needs?’ A key goal was to uncover underlying theories or mechanisms operating in behavioural HIV prevention interventions with immigrants, to uncover explanations as how and why they work (or not) for particular groups in particular contexts, and thus to refine the underlying theories. The realist review mapped seven initial mechanisms underlying culturally appropriate HIV prevention with immigrants. Evidence from intervention studies and qualitative studies found in systematic searches was then used to test and refine these seven mechanisms.

**Results:**

Thirty-four intervention studies and 40 qualitative studies contributed to the analysis and synthesis of evidence. The strongest evidence supported the role of ‘*consonance*’ mechanisms, indicating the pivotal need to incorporate cultural values into the intervention content. Moderate evidence was found to support the role of three other mechanisms – ‘*understanding*’, ‘*specificity*’ and ‘*embeddedness*’ – which indicated that using the language of immigrants, usually the ‘mother tongue’, targeting (in terms of ethnicity) and the use of settings were also critical elements in culturally appropriate HIV prevention. There was mixed evidence for the roles of ‘*authenticity*’ and ‘*framing*’ mechanisms and only partial evidence to support role of ‘*endorsement*’ mechanisms.

**Conclusions:**

This realist review contributes to the explanatory framework of behavioural HIV prevention among immigrants living in high-income countries and, in particular, builds a greater understanding of the suite of mechanisms that underpin adaptations of interventions by the cultural context and population being targeted.

## Background

### Overview of HIV among immigrants in high-income countries: a global and local problem

HIV is one of the major global public health challenges of the past 30 years with the burden of morbidity and mortality concentrated in developing and middle-income countries. In many of these countries there is a generalised HIV epidemic attributed mainly to heterosexual transmission [1]. In contrast, in many high-income countries, HIV generally emerged in sub-populations of gay men, people who inject drugs and sex workers [2]. While behavioural approaches were the main focus of early efforts in HIV prevention, more recently, biomedical approaches and structural approaches have gained currency as essential components of what is termed “combination HIV prevention” [2,3]. Despite these shifts, behavioural approaches to HIV prevention remain central to public health efforts to contain HIV in high-income countries.

HIV has spread to all countries, with unprotected sex between men and women and men and men the most common routes of transmission [4]. The epidemic has not affected countries equally. In 2007, more than 95% of new HIV infections worldwide occurred in developing and middle-income countries and more than 65% of AIDS-related deaths occurred in sub-Saharan Africa [1]. Globally there are some promising indications that HIV is being contained in many parts of the world [3]. In high-income countries the earliest examples of successful behaviour change were observed in North America, Europe and Australasia among communities of gay men and people who inject drugs, resulting in a containment of HIV from the ‘general’ population in many of these countries. These early successes have been difficult to sustain over 3 decades [2] and new prevention challenges are emerging.

One of these challenges in high-income countries has been the upswing of HIV among immigrants [5]. This trend has been reported among immigrants in parts of the European Union [6-8], other European countries [9], the USA [10-13], Canada [14,15] and Israel [16]. This trend is concentrated among immigrants from developing and middle-income countries in sub-Saharan Africa, Asia and the Caribbean where HIV is more prevalent, reflecting the global (and unequal) distribution of HIV, and the trend is characterised as affecting multiple communities (or ethnicities) of immigrants. Heterosexual transmission is more common among these immigrants reflecting the dominant pattern of HIV transmission in countries of origin [6,8,9,12,14,17,18]. However some epidemiological studies have noted the significant proportion of HIV cases among immigrant gay men across the European Union [8], in the UK [19] and in the USA [20].

There is a growing body of evidence of inequalities being experienced by immigrants in high-income countries in the context of HIV. Epidemiological studies have found different prevalence rates between immigrants and host populations in the opportunistic infections of HIV disease – with, for example, higher rates of TB observed among HIV-positive immigrants in France [21], the USA [22] and Australia [23]. Other epidemiological studies have documented disparities between immigrants and locally born populations in terms of later presentation with HIV [24]. Early presentation and early treatment of HIV have significant individual and public health benefits in terms of reducing morbidity and mortality and reducing HIV transmission [25,26]. Epidemiological studies have reported later presentation among African and Caribbean immigrants in Europe [27-29], among Latinos [18,30], Asian and Pacific Islanders [31], and African and Caribbean immigrants in the USA [17] and among Asian and sub-Saharan African immigrants in Australia [32-34]. It is important to note that these disparities between immigrant and locally born populations in high-income countries are mainly observed among immigrants born in developing and middle-income countries rather than immigrants born in other high-income countries [8,24,35]. The decline observed in AIDS reports across the European Union attributed to the impact of HIV treatments has not been seen among immigrants from developing and middle-income countries and has been characterised as a failure of both primary and secondary HIV prevention [8].

Population mobility and migration are unprecedented today in volume, speed and reach [36]. With more rapid and frequent travel, the multiple health environments that immigrants reside in, and return to, can become more closely linked. Thus “merging health environments reflect the socio-economic and cultural background and disease prevalence of communities of origin, transit, destination and return” ([37] p. 26). Population mobility and migration are entwined in the history of infectious diseases such as TB and hepatitis B (with higher rates observed among immigrants) [37-39]. In a HIV context, population mobility and migration are thought to have contributed to the spread of HIV by bringing populations with different background prevalence rates of HIV into closer proximity with each other [39,40]. The complexities and interactions between the local and global dimensions of public health are part of a wider consciousness of the challenges of promoting health in an era of globalisation [39,41].

### Behavioural HIV prevention in high-income countries

A number of biological and behavioural factors are known to affected HIV transmission, including the average probability of transmission, the number of exposures, viral load, co-infection with other sexually transmissible infections and the prevalence of HIV within a given population [4]. These proximal factors are often assumed to be the ‘real stuff’ of HIV prevention [3]. There is now a growing acceptance that distal contextual factors such as societal and cultural norms, gender inequalities and socioeconomic variables are important influences in HIV transmission [4,42].

Health promotion has been at the heart of public health over the last 20 years around a central tenet to enable individuals and communities to take control of their health [43,44]. The span of behavioural HIV prevention interventions can be stratified into individual-level interventions that primarily aim to influence personal knowledge, attitudes, motivations and skills; group-level and community-level interventions that attempt to influence personal skills and information with peers, groups or wider social networks; and structural interventions that aim to support change and remove barriers conducive to health across populations [2]. HIV prevention in high-income countries has utilised individual, group, community and structural interventions [45] drawing on a wide range of health promotion theories and models [44,46,47] with a growing shift away from individual behaviour change toward community-level and structural interventions [2,3,42]. Behavioural interventions that explicitly include HIV-positive people in prevention efforts have gained currency in high-income countries in the era of effective HIV treatments to minimise the likelihood of HIV-positive people being re-infected with other strains of HIV (or other sexually transmissible infections) and to reduce the risk of onward transmission of HIV [2,48].

Behavioural HIV prevention in high-income countries has also balanced the need for whole-of-population approaches and targeted approaches that address specific sub-populations that are disproportionately affected by HIV [45,49]. Targeted approaches have been defined as those that take into account the shared characteristics of the members of a sub-population [3,50]. The pitfalls of targeted approaches are that they rely on assumptions of homogeneity in the sub-population [50] and that they can contribute to marginalisation and discrimination of these populations [49]. Targeted approaches are often grounded in community engagement and community mobilisation [3,45] to ensure that the methods and communication used in HIV prevention is congruent with the behaviours and practices of the target group themselves, uses language that is readily understood and invokes culturally specific values – all hallmarks of a culturally appropriate approach [49,50].

### Behavioural HIV prevention with immigrants in high-income countries

At a time of increasing priority of the ‘problem’ of HIV among immigrants in high-income countries [5,8], there is a growing need to build the evidence base to guide targeted and culturally appropriate behavioural HIV prevention for immigrants. Targeted approaches have been utilised in many high-income countries and have included interventions with the ‘general’ immigrant population [51] or specific populations within immigrant communities such as gay men [52], people who inject drugs [53] and women [54].

A preliminary search of the literature found few reviews of evidence that analysed and synthesised insights from group- or community-level behavioural HIV prevention with immigrants in high-income countries [18,55-60]. While many of the reviews touched on the adaptation of interventions to address cultural appropriateness, only one addressed in any detail the mechanisms by which this was achieved [58]. Typically these reviews reported on the dearth of published studies of behavioural HIV prevention with immigrants that met the inclusion criteria [18,56-58,60]. In light of this it was important to shift from the narrow focus of ‘conventional’ systematic reviews that often see study design and outcome measures as the key inclusion criteria and attempt instead to gain insights into the processes, theories and mechanisms underpinning interventions with immigrants that might inform policy and practice. As Petticrew [61] notes, “policymakers…are less interested in evidence we don’t have, than in which direction the evidence is pointing (with suitable caveats)” (p. 411). In areas where the evidence base is ‘weak’, we need to make the best use of the available evidence, whatever its limitations, in our efforts to uncover the mechanisms that might be contributing to outcomes [61-63]. In the context of behavioural HIV prevention with immigrants, we also need to try to uncover mechanisms that might potentially be transferable across multiple immigrant populations and different contexts.

## Methods

### A realist review: an opportunity to add to the evidence base

A fundamental goal of public health interventions is to influence human volition across populations to equip, mobilise and enable people to create their own health [39,43]. The extensive work of the World Health Organisation’s Commission on the Social Determinants of Health has added to the central argument that health is created or retarded in wider social contexts [64]. Thus an intervention, or set of interventions, may succeed or fail depending on the wider social systems in which they are implemented. The nature of public health interventions can also mean that outcomes are harder to evaluate as it is often difficult in a programme that is emergent, and attempting to be participatory, to isolate and predict what activities may have contributed toward any observed change in outcomes [65]. Reviews of evidence to inform behavioural HIV prevention tend to be dominated by ‘conventional’ systematic reviews that use pre-defined outcome measures and study design inclusion criteria and can often fail to show positive or negative effects [4]. To date, systematic reviews of HIV prevention with immigrants in high-income countries have followed a similar trajectory [18,56] and have been constrained by the paucity of primary studies meeting pre-defined inclusion criteria.

We addressed these challenges in this review of evidence by adopting a realist methodology [66], a relatively new approach to knowledge synthesis, which is concerned with explaining more fully the context, processes (or theories) and outcomes of interventions. The realist approach to reviewing evidence is based on the assumption that no deterministic theories can predict outcomes in all contexts [67]. Rather, behind the processes of an intervention there is an underlying mechanism (or mechanisms) that causes the change (or outcome) to occur under specific contexts [68]. Realist reviews can contribute to programme understandings even when the outcomes are not rigidly defined at the outset of the review [66] and have been characterised as a theory-driven and interpretive approach to systematic reviews [69] to answer questions about what works, for whom and in what circumstances [67]. At the time that we were undertaking this research, there were very few published realist reviews and consequently limited guidance on the methods to be followed. Some researchers have argued for greater clarity around review designs as a way to address the current proliferation of types of systematic reviews and provide better guidance on the methods and terminology to be followed with each type of review (in particular for new and emerging methods) [70]. In their typology realist reviews are configurative reviews where the prime purpose is exploratory and they can accommodate different types of empirical evidence [70]. There are currently attempts underway to strengthen the methodological guidance for undertaking realist reviews [71].

A preliminary scan of the literature revealed that most behavioural HIV prevention interventions with immigrants had been adapted to make them ‘culturally appropriate’, but that this was largely based on implicit theories for achieving ‘cultural appropriateness’. Consequently, this shifted the primary purpose of this review to surface these implicit theories and see how they worked in different contexts. Thus the research question became: *What are the key mechanisms for cultural appropriateness operating in behavioural HIV prevention interventions at group and community levels among immigrants from developing and middleincome countries who live in highincome countries*? A realist review [66] embraces the opportunity to glean explanations from the differing reactions that immigrant populations have to behavioural HIV prevention interventions across social systems in multiple high-income countries. These different contexts, while challenging to analyse and synthesise, are nonetheless necessary to develop greater understandings of successes and failures (and all points in between) [66,72] of behavioural HIV prevention with immigrants and the resulting findings can “act as the initial empirical guide for future optimal locations [for interventions]” ([66] p. 22). This focus is also where realist reviews dovetail with the ‘theory-driven’ family of evaluation methodologies and, in this review of evidence, the methods developed by Weiss [73,74] were critical.

The preliminary scan of the literature also uncovered studies that explored the perspectives of immigrants towards HIV. Lay perspectives – what Harden and her colleagues [75] call “views studies” – place “people’s own voices at the centre of the analysis” (p. 794) and are well suited to harness evidence to answer questions of the cultural appropriateness of interventions [76-80]. Thus the centrality of the cultural appropriateness of HIV prevention in the research question pointed to a review of two ‘types’ of primary studies: behavioural intervention studies and studies that explored the views of immigrants themselves towards HIV prevention. Realist reviews of evidence have been categorised as one of the “narrative approaches” [78] to systematic reviews suited to integrating a heterogeneity of quantitative and qualitative evidence [70]. Methods to integrate intervention studies and the views of immigrants in this review of evidence drew on reviews carried out by the EPPI-Centre in the UK [75,80-82]. Finally, the focus in this review of evidence was on group- and community-level interventions to address the growing recognition of the limitations of individual-level interventions, particularly in preventing the sexual transmission of HIV [2,3,42].

### Systematic searching for primary studies

Four electronic databases, PubMed, CINAHL, PsychInfo and Sociological Abstracts, were chosen for this review following guidance from related systematic reviews [18,80] and handbooks [79,83]. Sociological Abstracts allowed for simultaneous searching of Social Services Abstracts, ERIC (an education database) and PAIS (a public affairs database). This was supplemented by a Google Scholar search to enhance the possibility of picking up ‘grey’ literature. The Expert Reference Group (see Acknowledgements) – made up of international researchers and practitioners with expertise in HIV prevention among immigrants – were consulted on the suitability of the proposed databases and Google Scholar to retrieve primary studies including ‘grey’ literature.

We framed the search strategies having categorised interventions into their constituent ‘parts’. We used the categories adopted by the Cochrane Health Promotion and Public Health Field [79], which frame interventions in terms of PICO(T) – Population, Intervention, Comparison, Outcome and Type (of study design). This review was concerned with the ‘population’ of immigrants from developing or middle-income countries and the ‘intervention’ of behavioural HIV prevention at group and community levels. In keeping with a realist methodology [66], we did not limit the framing of search strategies in terms of comparisons, outcomes or types of study design as we primarily wanted to capture studies that could inform programme theory in this area to answer ‘how and why’ questions as well as ‘what works’ in these interventions. Similarly, the framing of search strategies for views studies was defined around the ‘population’ of immigrants from developing or middle-income countries as well as attitudes toward behavioural HIV prevention.

Separate search terms were developed to search for intervention and views studies on each of the four databases but followed essentially the same process described below. Developing search terms is often defined in terms of a trade-off between sensitivity and specificity [78,84,85]. With this in mind we followed the methods reported by others in a review of sexual health promotion interventions [82]. The starting point was to bring together two ‘known sets’ of primary studies of relevance to the review question ([82] p. 47). These ‘known sets’ were comprised of studies known to the lead author and studies found in preliminary searches of databases in the initial stages of the review. These ‘known set’ studies did not necessarily meet our inclusion criteria (which we developed iteratively later). In our ‘known sets’ of primary studies we purposively aimed to include evidence from a variety of contexts in high-income countries, a variety of immigrant populations, and a variety of study and intervention types. These ‘known sets’ of 20 intervention studies (see Additional file 1) and 20 views studies (see Additional file 2) were then traced back to the four databases and, if found, the controlled vocabulary under which each study was indexed on that database was noted [82]. This controlled vocabulary for the 40 ‘known set’ studies was scanned to generate search terms for pilot searches [78,79,82] on each database. These pilot searches, eight in all, allowed for an iterative refinement of the search terms [84] – expanding the search terms if ‘known set’ studies indexed on that database failed to be found or narrowing the search terms, without losing ‘known set’ studies, if too many citations were generated. This meant that search terms generated from the controlled vocabulary of ‘known set’ studies on each database were tested and refined. In addition, the final search terms were circulated to an Expert Reference Group (see Acknowledgements), and their feedback on the comprehensiveness of the proposed search terms and proposed databases was sought and incorporated. The final searches on electronic databases were carried out between November 2007 and January 2008.

### Methods for identifying candidate mechanisms

**Figure 1:**
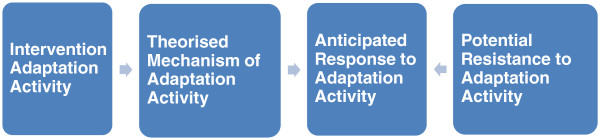
An intervention implementation ‘chain’.

In a realist review one of the first steps is to develop an explanatory framework of interventions that are being implemented in the field [66,68]. The search attempts to uncover administrative thinking, policy history and key points of contention that lie behind the family of interventions [66,69] – in this case behavioural HIV prevention with immigrants. Papers found in the preliminary scan of the literature were reviewed and analysed for dominant themes as to ‘how’ and ‘why’ interventions with immigrants were supposed to work. A key theme was one where ‘cultural appropriateness’ was assumed as a key principle for HIV prevention in this area but where this principle was rarely defined or discussed in detail [50,86-89]. The papers were then reviewed to explore the common intervention activities in behavioural HIV prevention with immigrants. Weiss [74] frames interventions as a series of implementation ‘chains’ comprising intervention actions and participant reactions. We have represented the ‘chain’ in our review as being made up of an adaptation activity that generates an anticipated response and a potential resistance to the intervention from immigrants (Figure 1) [73]. Intervening between the adaptation activity and anticipated response by immigrants are the theorised mechanisms – the ‘change elements’ – of the intervention (Figure 1) [73]. It is important to note that in reality these ‘chains’ can operate in non-linear and unpredictable ways depending on the context [68]. Here, for simplicity, the implementation ‘chain’ is presented in a linear ‘path’ with the participant response and participant resistance represented as outcomes that point in different directions. In reality, these two different outcomes can be alternate reactions triggered by the context of the mechanism. The broad adaptation activities being implemented to make HIV prevention interventions more ‘culturally appropriate’ were across the dimensions of ‘staffing’, ‘language’, ‘content’, ‘ethnic diversity’, ‘settings’, ‘community consultation’ and ‘priority setting’. These adaptation activities were the first step for the development of the candidate mechanisms and the results of this first step are made available elsewhere (see Additional file 3).

Generating hypothesised mechanisms underlying the adaptation activities are integral to understanding ‘how’ and ‘why’ behavioural HIV prevention with immigrants ‘works’. Weiss [74] suggests harnessing the perspectives of programme stakeholders as a key source of candidate mechanisms. Programme theory deals with “the mechanisms that intervene between the delivery of the program … and the occurrence of outcomes of interest. It focuses on participants’ responses to [the] program” ([73] p. 73). So in HIV prevention interventions with immigrants we need to understand the inducements to change offered by the intervention (e.g., you will worry less if you know your HIV status) and these can only work as intended if participants go along with these inducements (e.g., agree with the proposition that an HIV test will lead to reduced anxiety about HIV).

The second step in identifying candidate mechanisms involved the lead investigator testing the initial adaptation activities in interventions – ‘staffing’, ‘language’, ‘content’, ‘ethnic diversity’, ‘settings’, ‘community consultation’ and ‘priority setting’ – against the literature of the 20 ‘known’ intervention studies (see Additional file 1) and other papers on HIV prevention among immigrants, listing the responses/resistances of intervention participants to these activities and interpreting the candidate mechanisms operating between the activities, responses and resistances [73]. This process iteratively developed seven hypothesised ‘chains’ arising from the adaptation activities operating in culturally appropriate HIV prevention with immigrants [74]. The results of these activities, responses, resistances and candidate mechanisms are described more fully elsewhere (see Additional File 3). The seven candidate mechanisms – ‘*authenticity*’, ‘*understanding*’, ‘*consonance*’, ‘*specificity*’, ‘*embeddedness*’, ‘*endorsement*’ and ‘*framing*’ – were ‘the lens’ [66] to analyse the evidence from intervention and views studies to refine each adaptive mechanism in behavioural HIV prevention interventions with immigrants. These seven mechanisms were theorised as the key, rather than the only, interrelated mechanisms contributing to cultural appropriateness in interventions with immigrants and will be discussed in detail in the results section but are summarised in a case study below.

### A case study of the candidate mechanisms

Taking an example of an intervention with Latino immigrants in the USA, ‘*authenticity*’ and ‘*understanding*’ mechanisms are hypothesised to operate when, for example, Latino facilitators delivered a series of HIV education sessions to groups of Latino immigrants in Spanish. ‘C*onsonance*’ is hypothesised to be operating when Latino cultural norms such as *machismo* are drawn on for the intervention content. ‘*Specificity*’ is hypothesised to be operating when Mexicans, rather than Latinos, are specifically targeted by the intervention. ‘*Embeddedness*’ is hypothesised to operate when an intervention is delivered at farms where Mexican migrant day labourers work. The ‘*endorsement*’ mechanism operates when an intervention consults extensively with Mexican immigrants to garner their support for intervention strategies. Finally, the ‘*framing*’ mechanism is operating when the intervention partners with a Mexican community organisation in ‘priority setting’ around intervention goals.

### Inclusion criteria - appraising the relevance of the evidence

Appraising studies for relevance in realist reviews is less predetermined at the outset when compared to a ‘conventional’ systematic review [90,91]. Pawson [66] asserts that as the prime focus is to explore underlying theories of programmes (rather than the programmes themselves), a wider range of primary studies may be relevant to the analysis and synthesis. The resources available for this doctoral research meant that it was not feasible in this review to follow this path. Instead, inclusion criteria for intervention and views studies were generated drawing on guidance from related systematic reviews [56-58,80] and refining them iteratively in light of the focus of the review. Intervention and views studies were included if the study:

Focused on HIV/AIDS

Focused on immigrants from developing or middle-income countries [92]

Was carried out in high-income countries [92]

Was available for review in English

And more than 65% of intervention participants were reported to be (or could be inferred to be) immigrants from developing or middle-income countries [92]

In addition, intervention studies were included if:

It was implemented at a group or community level

View studies were included if the study:

Focused on the views of immigrants – “as to what helps or hinders them in relation to … [HIV/AIDS] and about their perceptions of HIV-related … health” ([80] p. 19)

These criteria generated a feasible number of intervention studies for reviewing full reports. However, as there were more than 350 views studies still retained after reviewing the abstracts, two additional criteria were added to retain studies if:

Qualitative research methods were used including studies where this was in conjunction with, or preceded by, quantitative research methods

Qualitative studies had successfully recruited more than 15 immigrant participants

The decision to include this final criterion was a pragmatic one to cull the large number of abstracts to a feasible number.

In practice, the filtering of intervention and views studies occurred by progressively applying these inclusion criteria. For example, when reviewing the relevance of a title of a primary study it was usually only possible to assess the title against the criteria ‘HIV/AIDS’, that it was available in English and that it had a focus on immigrants or ethnic minorities. If the lead author was in doubt about the relevance of a title it was retained for later appraisal at the abstract reviewing stage. Similarly, if there was doubt about the relevance of an abstract it was retained for appraisal at full-text stage. It was often only possible at the full-text stage to assess the study against all of our inclusion criteria.

### Quality frameworks - appraising the rigour of the evidence

Quality appraisal of primary studies is often in-built to the exclusion criteria of a ‘conventional’ systematic review through the use of study design as a key inclusion criterion [79]. This is less appropriate in a realist review that is centrally concerned with the processes and mechanisms of interventions [66]. Instead Pawson [66] argues that “the appraisal criteria should be subordinate to the usage to which the primary study is put” (p. 87). In this review appraising the rigour of intervention studies was guided by one principle – did the inferences drawn have sufficient weight to make a methodologically and conceptually sound contribution to the test of theories in this review. This principle translated primarily into a check when reviewing full reports, borrowed from a quality appraisal framework developed by the EPPI-Centre, ([79] p. 52) around whether the study described the key processes involved in delivering the intervention that related to the adaptive mechanism. This could either be confirmatory or contradictory evidence. Appraising the rigour of views studies was guided by the same general principle – did the inferences drawn have sufficient weight to make a methodologically and conceptually sound contribution to the test of theories in this review. This principle translated into using checks adapted from a quality assessment framework by the EPPI-Centre of views studies ([80] pp.22-23).

### Data management, analysis and synthesis methods

In realist reviews, analysis and synthesis of the evidence tend to occur alongside each other [66]. Full reports of 164 intervention studies (2 of which were unable to be found) were appraised for relevance and rigour, and this resulted in 34 studies being retained for the analysis and synthesis (see Additional file 4). The full reports of 110 views studies (1 of which was unable to be found) were appraised for relevance and rigour, and this resulted in 40 studies being retained (see Additional file 5). The studies retained for analysis and synthesis included studies uncovered after the Expert Reference Group (see Acknowledgements) of international experts provided feedback on an earlier shortlist of intervention and views studies. The studies suggested by the Expert Reference Group resulted in one full report of an intervention and five full reports of views being included after being appraised for relevance and rigour.

### Collation of studies included in the analysis and synthesis

The full reports of primary studies, 34 intervention studies and 40 views studies, were collated and annotated into templates so that evidence could begin to be extracted for the analysis and synthesis [66]. For intervention studies, a table adapted from a related review [58] was used. Each report was reviewed to collate the key elements into a descriptive table (see Additional file 4). A similar descriptive table was adapted [58,80] to collate the key elements of views studies (see Additional file 5).

Each intervention and views study was then annotated into two additional templates so that results from each report could be extracted for analysis and synthesis [66,91]. Each report was rated against the seven adaptive mechanisms – ‘*authenticity*’, ‘*understanding*’, ‘*consonance*’, ‘*specificity*’, ‘*embeddedness*’, ‘*endorsement*’ and ‘*framing*’. The process was undertaken twice by the lead investigator to achieve an initial tentative rating and then reviewed to obtain a final rating. The outcomes of this annotation process are summarised in a table for intervention studies (see Additional file 6) and a table for views studies (see Additional file 7), and more details on this rating process are provided elsewhere (see Additional file 8).

## Results and discussion

### Search results

Searches for intervention studies yielded 3,323 records that were culled to 1,061 records after reviewing the title. Searches for views studies, yielded 2,715 records, which were culled to 868 records after reviewing the title. In the Google Scholar search for intervention and views studies only the first 1,000 titles could be reviewed. Amalgamation of duplicate search records at two separate points resulted in 282 intervention studies and 384 views studies being culled. Abstracts of 848 records of intervention studies and 744 records of views studies were appraised for relevance against the inclusion criteria. This process is summarised in a PRISMA flow diagram elsewhere (Additional file 9). Full reports of 164 intervention studies were appraised for relevance and rigour, and this resulted in 34 studies being retained for the analysis and synthesis (see Additional file 4). The full reports of 110 views studies were appraised for relevance and rigour, and this resulted in 40 studies being included in the review (see Additional file 5).

The majority of the intervention studies retained for analysis was conducted in the USA, with three studies in Israel and The Netherlands, and one each in Switzerland, Australia, Canada and New Zealand. In some cases the studies reported on different aspects of an intervention. In all, the 34 reports on 30 distinct interventions were almost equally divided between group-level and community-level interventions with a mix of interventions targeted to the ‘general’ immigrant community or specific subgroups such as gay men or women. Almost all the interventions focussed on primary HIV prevention, with only two targeting HIV-positive immigrants. The majority of the views studies retained for analysis was conducted in the USA, with eight studies carried out in the UK, seven in Australia, and one each in Sweden, Canada and Japan. About half of the studies reported on the views of HIV-positive immigrants, and there was a mix of study populations in terms of gender and sexual orientation. In all the 40 views studies reported on 28 distinct research projects.

### Results: generating candidate mechanisms

The initial explanatory framework of behavioural HIV prevention explored the adaptations in the literature in relation to ‘how’ and ‘why’ interventions with immigrants were supposed to work and their limitations [66,68]. The results of this first step and the literature that informed this process are detailed more fully elsewhere (see Additional file 3). The intervention adaptation activities – ‘staffing’, ‘language’, ‘content’, ‘ethnic diversity’, ‘settings’, ‘community consultation’ and ‘priority setting’ – were then further tested using methods described earlier against the literature from the ‘known studies’ and other studies to populate seven intervention ‘chains’, each made up of activities, mechanisms, responses and resistances [73,74], and led to the development of the candidate mechanisms. The results of this second step and the literature that informed this process are provided elsewhere (see Additional file 3) In summary, seven mechanisms – ‘*authenticity*’, ‘*understanding*’, ‘*consonance*’, ‘*specificity*’, ‘*embeddedness*’, ‘*endorsement*’ and ‘*framing*’ – were theorised to be operating in behavioural HIV prevention interventions with immigrants in high-income countries.

These steps in reviewing and analysing the literature also pointed to higher-level structural issues that can influence HIV prevention with immigrants that are not accounted for in this mapping of mechanisms. These include facilitating access to primary health care (e.g., for voluntary HIV testing) and to safe working and living environments (e.g., through ensuring that temporary working visas protect the immigrants’ human rights) [93]. While these structural issues are likely to significantly impact on the HIV interventions with immigrants, a detailed analysis of their impact was outside of the scope of this research.

In keeping with a realist synthesis, the primary data of intervention and views studies found in systematic searches was then used to test and refine [66,67] the seven adaptive mechanisms – ‘*authenticity*’, ‘*understanding*’, ‘*consonance*’, ‘*specificity*’, ‘*embeddedness*’, ‘*endorsement*’ and ‘*framing*’. In realist reviews non-equivalence of interventions and primary studies is the norm; thus, one way to synthesise and organise the evidence is to present the findings not study by study, but programme theory by programme theory [72]. The findings reported here are based on a much larger unpublished synthesis. We will now present the evidence from intervention studies and views studies for the four adaptive mechanisms where we found the strongest evidence. The results for three other adaptive mechanisms – ‘*authenticity*’, ‘*framing*’ and ‘*endorsement*’ – are provided elsewhere (see Additional file 10). The evidence is presented under headings as if the mechanisms were linear ‘chains’ from activity through to response and resistance influenced by context, whereas in reality these mechanisms may fire or misfire dependent on the context.

### Results: evidence synthesis and discussion

#### The evidence around ‘consonance’ mechanisms

##### Activity

**Figure 2:**
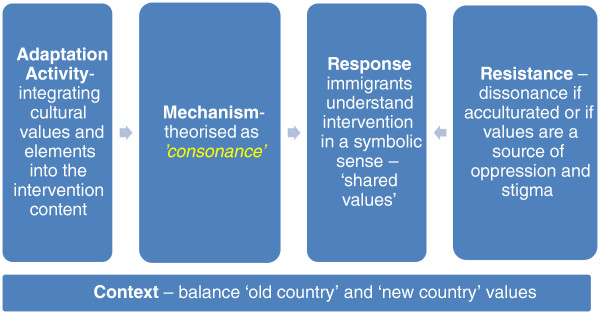
**‘*Understanding*’ mechanisms.**

The evidence from the intervention studies strongly supported the importance of this mechanism – where the content of the intervention is ‘consonant’ with the existing values of immigrants – in behavioural HIV prevention (Figure 2). Thirty-five interventions reported sound or moderate evidence of this mechanism, indicating the pivotal role of this adaptive mechanism in contributing to cultural appropriateness in interventions with immigrants. This mechanism was found to be operationalised primarily through drawing on research or consultations to elicit dominant cultural values, which were then incorporated into the intervention content [51,54,94-112]. In some interventions, such as those carried out with gay men, the dominant immigrant community values on homosexuality were juxtaposed with the dominant cultural values in the mainstream gay community in the destination country, leading to interventions for Latino and Asian gay and bisexual men that addressed positive ethnic and sexual identities [94,95,97,113]. Sound or moderate evidence to support this mechanism was reported in 37 views studies indicating that ‘*consonance*’ mechanisms were also seen as critical by immigrants themselves. There were a range of dominant interrelated themes in the views studies that pointed to broad commonalities across ethnicities, countries of origin and experiences of migration that, in the views of immigrants, impacted on HIV prevention efforts. These included the association of HIV with risk groups – such as homosexuals, prostitutes and people with multiple sexual partners – and a strong theme of not personalising HIV risks [114-121]. This sense that HIV happened to certain ‘kinds of people’ often resulted in profound feelings of shock when immigrants were diagnosed with HIV, such as those expressed by an African-born HIV-positive woman in the UK: “I wasn’t that sort of person” ([114] p. 100).

This mechanism also influenced the use of culturally appropriate communication tools in behavioural HIV prevention. Proverbs were employed with Latino gay men in New York City [122] and in an intervention with newly arrived Ethiopian immigrants in Israel [123]. Other communication tools, already well recognised within Latino immigrant communities, included *fotonovelas* (low-literacy Latino comic books), *radionovelas* (Latino radio dramas), *lotteria* (Mexican tarot) cards, [94,106,124] and theatre [102] to enhance communication around HIV. In two interventions with African-born immigrants in the UK, one used theatre and the other a soccer tournament to enhance communication around HIV [105].

##### Response and resistance

The theorised response of immigrants to this mechanism is that the intervention has recognisable elements of their own culture around a notion of ‘shared values’. However, in general the intervention studies did not report feedback of programme participants on this specific mechanism. Some positive impacts of this mechanism could be inferred. One study reported favourable changes in beliefs about gender roles among Latina women [101] and another study reported greater connectedness to Latino gay community networks [97], though neither of these outcomes can be directly attributed to the incorporation of cultural values into the interventions. Two interventions with African-born immigrants in the UK reported positive feedback in the use of theatre and soccer as effective communication tools where there was a potential for a racist backlash from the mainstream community, some of whom associated HIV with the inward migration of African-born refugees and asylum seekers [105].

As most views studies were concerned with the sexual transmission of HIV, there were a multitude of potential resistances to the goals of the intervention reported by immigrants dependent on the cultural context. These included: condoms as barriers to intimacy [125-127], gender roles and the negotiation of condoms [127-130], associations of condoms with contraception [131] or promiscuity [132], and homophobic attitudes in some cultural contexts [133,134]. Here it is not possible to list all the reported resistances but to underscore that while the cultural contexts may not cause behaviours they may shape both protective and risk behaviours in HIV prevention [117,127].

##### Context

Incorporating cultural values, especially attempts to find ‘shared values’, into the intervention content sometimes involved a nuanced interpretation of what the dominant immigrant cultural values were – as these often clashed with the cultural norms of the destination country around gender and sexuality. Other interventions explicitly explored ‘old-country’ and ‘new-country’ norms in relation to HIV as exemplified in a programme with newly arrived Ethiopian immigrants to Israel [109]. A range of difficulties was reported by immigrants in negotiating and integrating competing values, experiences and information from their country of origin with those in the ‘new’ country [116,119,120,125,132,135-142]. This sense of living between ‘two worlds’ was characterised by a struggle between the retention or rejection of the past and the ‘old’ country. An example of this struggle was the retention of lay health understandings about the causal mechanisms of HIV transmission and strategies for HIV prevention based on (erroneous) notions of ‘cleanliness’ [125,139,141] that are in contrast to the biomedical understandings of HIV transmission used in destination high-income countries. Another example was the widespread perception expressed by many HIV-positive immigrants that an HIV diagnosis was ‘AIDS’ and therefore a ‘death sentence’, which was sometimes related to information deficits (e.g., not knowing about HIV treatments or the difference between HIV and AIDS) but often was also related to direct, and often harrowing experiences, of knowing people with HIV in their ‘old countries’ where the stigma around HIV was profound and access to treatments difficult or impossible [116,118-120,127,143,144].

The often high levels of stigma associated with HIV among immigrant communities led to a strong culture of silence and secrecy [114,115,118,120,121,127,128,133,138,140,143-147]. This silence and secrecy extended to any discussion of HIV in social and community contexts and – among people who were HIV-positive – contributed to a strong reluctance to disclose their HIV status – especially in co-ethnic social networks. Indeed the need for secrecy for HIV-positive immigrants – an expression of ‘old-country’ values – was put forward as an important daily challenge [118].

Other dominant themes in cultural values that impacted on HIV prevention included the differences between male and female gender roles among many immigrant communities [127-129,136,137,142,148-150], which in the studies of Latino communities were often described using concepts such as *machismo*. “To be a strong male is to get sex and to be a strong woman is to resist sex” ([136] p. 411). This theme was also expressed by Latino gay men whose sexual identity was often constructed in line with dominant gender roles [133,134]. Some Latina HIV-positive women who had experienced domestic violence saw gender roles as a contributing factor to both their experiences of violence and acquiring HIV [127].

This mechanism also raises specific challenges for the design and implementation of interventions in seeking to find an appropriate balance between ‘old country’ and ‘new country’ – or the ‘past’ and the ‘present’ context – across a range of dimensions including norms [109], values, experiences [116,120,132,135,137,138,142] and understandings of critical information around HIV [125,139,141]. The evidence to support this mechanism from the intervention and views studies was largely consistent with how the ‘*consonance*’ mechanism had originally been theorised. However, the evidence gave deeper insights into how ‘*consonance*’ is enacted, and potentially resisted, as a key mechanism to deepen the symbolic understanding by immigrants of the goals of HIV prevention interventions.

##### Discussion of ‘consonance’ mechanisms

The strong evidence to support this adaptive mechanism was consistent across the intervention studies and the views studies indicating the pivotal role of this mechanism in behavioural HIV prevention (including prevention efforts which involve people living with HIV) at group and community levels with a focus on ‘shared values’ to deepen the symbolic understanding of the intervention. This mechanism can be seen as an expression of the theme of ‘matching’ the intervention to the immigrant participants with a focus here on ‘matching’ the intervention with cultural values, norms and symbols to increase the symbolic understanding of interventions. However, studies that have attempted to empirically test if incorporating cultural elements contributes to the effectiveness of HIV interventions with immigrants have found mixed results [53]. There is support for ‘*consonance*’ as theorised in this review in the wider literature where there is a strong critique of cognitive and behavioural individualistic approaches to HIV prevention among immigrants for their failure to account for the wider impact of social and cultural influences on behaviour [18,50,52,93,151-155]. This mechanism also shares to some extent the territory of broader theoretical frameworks in health promotion interventions, in particular, the widely used social cognitive theory, which builds upon the importance of social norms [44,46].

However, this territory of norms and cultural values in HIV prevention with immigrants can present a range of important dilemmas. The first dilemma is that HIV interventions may need to challenge dominant cultural norms with ‘new-country’ values in the intervention content and thus the intervention may be seen to be subversive to deeply held values that have been ‘retained’ from the ‘old country’ [156]. Yet the same ‘subversive’ content may be appealing to other immigrants such as women or gay men who are potentially more likely to have experienced marginalisation within ‘old-country’ values [97,136,156]. In response to this issue Shtarkshall and Soskolne [109] propose using “cultural insighters” (p. 7) – immigrants drawn from the target community of the intervention – to negotiate this terrain and to address the tensions that have been found when attempting to reconcile immigrant and ‘new-country’ values in other areas such as education [157]. These “cultural insighters” could potentially contribute to two other adaptive mechanisms – ‘*authenticity*’ (see Additional file 10) and ‘*understanding*’ (reported below) – in the implementation of interventions.

A second dilemma raised by this adaptive mechanism relates to the limitations of ‘knowing’ what in fact the ‘cultural values’ are in any given immigrant population. In this review only the broad ‘Latino community’ [18], and to some extent Latino gay men [97], in the USA had a body of social sciences literature that could be drawn on by those designing an HIV prevention intervention for immigrants. In addition, even if you ‘know’ the cultural values you also need to disentangle those that are protective for HIV risk behaviours from those that point towards increased risk. This raises an important limitation in relation to this mechanism: in practice, many HIV prevention interventions may simply have to ‘fly blind’ with only informal research or community consultation processes, or follow Shtarkshall and Soskolne’s [109] strategy of “cultural insighters” (p. 7), to guide the development of the content and implementation of interventions.

#### The evidence around ‘understanding’ mechanisms

##### Activity

**Figure 3:**
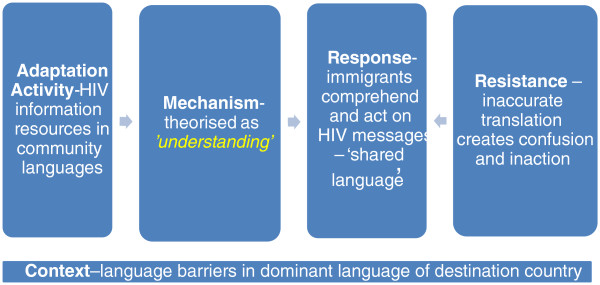
**‘*Consonance*’ mechanisms.**

The evidence from the intervention studies strongly supported the importance of this mechanism (Figure 3). Twenty-six interventions reported sound evidence indicating the strong role of this mechanism in culturally appropriate HIV prevention with immigrants. This mechanism was found to be operationalised primarily through the use of the first languages of immigrants themselves in interventions [51,54,94-98,101-104,107-111,122-124,158-162]. A further seven interventions reported moderate evidence of this mechanism [99,100,106,113,159,163,164] primarily through less comprehensive use of the language spoken by participants (e.g., only in supporting health promotion resources). Sound or moderate evidence to support this mechanism was reported in 18 views studies indicating that the ‘*understanding*’ mechanism was also valued by immigrants themselves in contributing to culturally appropriate HIV prevention [115,120,121,125-127,129-133,139,144,147,150,165,166]. There was partial evidence from a further eight studies for this mechanism largely inferred from the use of community languages by the researchers to successfully elicit the views of immigrants [117,119,135,137,141,142,148,149]. The strengths of ‘*understanding*’ mechanisms from the perspectives of immigrants were that communication (reading, writing, listening or speaking) in your preferred first language lowered the threshold of, and maximised participation and engagement with, intervention activities and, in turn, increased the opportunities to act on and benefit from the intervention.

##### Response and resistance

The theorised response of immigrants to this mechanism is that they ‘understand’ the intervention and the information that is being conveyed in a literal sense. However, in general, intervention studies did not report the feedback of programme participants on this specific mechanism. There was positive feedback of ‘*understanding*’ mechanisms reported in two studies of a single intervention with Latina immigrant women in the USA [99,100] and in an intervention with Turkish and Moroccan immigrants in The Netherlands [158]. There were reports of general satisfaction or improved recruitment to, or demand for, intervention activities among programme participants in five studies that employed this adaptive mechanism [51,54,95,110,124], though this could not be attributed specifically to the use of the first language of immigrants. The evidence to support this mechanism in views studies indicated that what immigrants themselves most value is ‘closeness’ in terms of ‘shared language’ [115,120,121,125-127,129-133,140,144,147,150,165,166]. Immigrants valued this mechanism for the potential of not relying on others (e.g., interpreters) or co-linguistic social networks (e.g., family and friends) to access information about a sensitive topic such as HIV or to navigate complex and unfamiliar health-care systems [115,120,121,125-127,129-133,140,144,147,150,165,166]. For example, immigrant sex workers in San Francisco relied on bilingual outreach workers to engage with HIV prevention interventions [130]. While the use of first languages is strongly supported by immigrants themselves in many contexts, it can present some additional challenges in HIV prevention. The cultural silences around HIV/AIDS observed among Chilean and Turkish women in a study in Melbourne were reported to be mirrored in these participants’ first languages – Spanish and Turkish [129]. Similarly, a study of the views of frontline Asian and Pacific Islander workers in the USA found that cultural taboos are often reflected in the language: “It [HIV prevention] seems harder [in the Cambodian language] because that language that we use … we don’t speak about sex in that language and it just seems so forbidden to speak about sex [in the Cambodian language] ” ([132] p.149).

The only explicitly reported resistance to this mechanism was the potential for mistranslation in an intervention with Vietnamese women in Los Angeles [98]. A related resistance to this mechanism reported in some studies, which overlaps to some extent with the ‘*authenticity*’ mechanism (see Additional file 10), was through the use of interpreters or translators as a way to operationalise this mechanism. This was viewed as sub-optimal as interpreters were not able to support or educate immigrants beyond language assistance [115,144], could be difficult to schedule or be a different gender, [115], could amplify concerns around confidentiality [120] or simply reinforce a reliance on a third party. As one Chinese HIV-positive immigrant in New York City put it: “Of course it’s better seeing a Chinese doctor. You don’t need a translator! You can just say whatever you want without having to go through another person” ([115] p.18).

##### Context

There was moderate evidence to support this adaptive mechanism across the intervention studies and the views studies indicating its key role in behavioural HIV prevention at group and community levels in contexts where there were language barriers for the immigrant population(s). This mechanism generally existed alongside the ‘*authenticity*’ mechanism in group-level interventions (see Additional file 10). In practice, this meant that bilingual staff were also bicultural and used both language and cultural skills to plan and deliver the intervention. The evidence to support this mechanism from the intervention and views studies was largely consistent with how the ‘*understanding*’ mechanism had originally been theorised. However, the evidence gave deeper insights into how this mechanism increases a literal understanding by immigrants of HIV prevention interventions.

The operationalisation of this mechanism in interventions where there was only partial evidence for ‘*understanding*’ mechanisms tended to be among immigrants who could be inferred to speak the language of the destination country as their lingua franca, as in the case of sub-Saharan African immigrants in the UK [105] or young immigrants from the former Soviet Union and Ethiopia in Israel [167]. Similarly, 14 of the views studies did not report any evidence around this mechanism in HIV prevention. As with the intervention studies, this may be due to these immigrants not experiencing significant language barriers in the destination country. Indeed 9 of these 14 studies were carried out in contexts where we can infer that most study participants spoke the dominant language of the destination country as their lingua franca: with seven studies carried out in the UK with sub-Saharan African participants, one study in New York City with Indian immigrants and one in Toronto with young Asian gay and lesbian participants. This points to the importance of this mechanism in contexts where the target immigrant population is known to have poorer spoken or written skills in the dominant destination country language or languages. However, a preference for accessing co-linguistic health professionals could potentially lead to using health-care workers who were less knowledgeable about HIV/AIDS [10]. In addition, language barriers were, in some instances, associated with gender [125,166]. For some HIV-positive women in Sydney, language barriers led to a reliance on their male partners, who were more proficient in English, to access HIV testing [166].

##### Discussion of ‘understanding’ mechanisms

The evidence suggests that what is valued most by immigrants, especially those who are ‘excluded’ by their limited language skills in the high-income destination country, is the opportunity to have a ‘shared language’. This is hardly surprising given the critical importance of language, and language proficiency, in complex human communication [50]. Using community languages can present both challenges and opportunities for interventions. Challenges in HIV prevention contexts for this adaptive mechanism as theorised in this review can include limited community language terms to communicate or talk about key risk behaviours like sex [132]. Opportunities can include the potential to use nuanced language, which may increase the literal understanding and even the symbolic understanding of the intervention among immigrant participants [129,132]. Some of the evidence in relation to ‘*understanding*’ in interventions points to the problems associated with translation (or rather mistranslation) in immigrant languages to communicate culturally appropriate messages [98]. The heterogeneity in terms of languages spoken by immigrants in many high-income countries can present a dilemma to the practical application of ‘*understanding*’ mechanisms. It may be hard to define which of the multiple languages spoken across immigrant communities to prioritise in the intervention with multiple translations potentially contributing significant costs, especially for smaller immigrant populations who were reported to be poorly served in terms of access to HIV information in their preferred language [115,165]. However, as noted earlier, in some contexts and with some populations, using community languages is less important in addressing barriers to HIV prevention. For example, in a comprehensive review of research relevant to the development of interventions for immigrants from sub-Saharan Africa in Europe, language did not feature as a significant barrier to be addressed in HIV prevention efforts [60], which may be due to a shared lingua franca – such as English or French, often derived from a colonial past – being widely spoken in these diverse African-born communities.

#### The evidence around ‘specificity’ mechanisms

##### Activity

**Figure 4:**
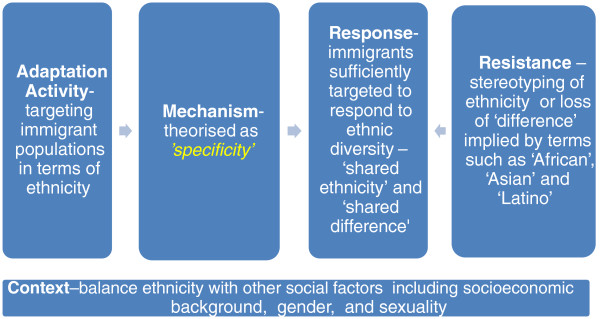
**‘*Specificity*’ mechanisms.**

The evidence from the intervention studies strongly supported the importance of this mechanism – where the ethnicity of immigrants is used to target behavioural HIV prevention (Figure 4). Seventeen interventions reported sound evidence and a further 15 interventions reported moderate evidence for this adaptation mechanism, suggesting a strong role for ‘*specificity*’ in culturally appropriate HIV prevention with immigrants. This mechanism was found to be operationalised primarily through segmentation of populations using ethnicity indicators, such as country of birth or language spoken, to target the intervention [98,102,105,109,123,124,158,162,168]. Another commonly used, though less specific, strategy was to target broader categories of immigrants such as Latinos, Asians, Pacific Islanders or Africans, and within these broader categories it was commonly reported that there was a dominant ethnicity, such as Mexican, Filipino or Ugandan, reached by the intervention [54,94,95,97,99-101,103,105-111,113,122,163,167]. Some interventions that reported only partial evidence of this mechanism could be inferred to have made a choice to target multiple ethnicities at the expense of ‘*specificity*’, as theorised in this review [96,104,105,113,164,167]. Similarly, an ethnic media campaign to promote HIV testing among immigrants in Australia [161] used ethnicity data from routine HIV notifications to prioritise the immigrant populations for a campaign that was implemented across 14 immigrant communities largely because this reflected the low but even spread of HIV across these multiple ethnicities.

The evidence to support this mechanism was weaker in views studies with moderate or sound evidence reported in 18 studies, indicating that this mechanism was not viewed as being as critical by immigrants themselves. Nonetheless, immigrants did highlight the important differences between ethnic groups who in some contexts share the same broad label – for example, Latino or Asian – such as the differences between Salvadorian and Chilean women in Melbourne [136,137], between Dominicans and Puerto Ricans [142] and between South Asian and Southeast Asian gay men in New York City [169]. Similarly, African-born HIV-positive women in the UK expressed a need for more ethnic-specific services – rather than the pan-African services, which already existed [146]. This highlighting of difference and heterogeneity can be viewed as supporting the need for ‘*specificity*’ to address the differences between immigrant populations that are often overlooked in interventions that attempt to reach ‘Africans’, ‘Asians’, ‘Latinos’ or ‘Pacific Islanders’.

##### Response and resistance

The theorised response of immigrants to this mechanism is that they perceive the intervention is sufficiently specific to their shared ethnicity around ‘shared difference’. Evidence to support this mechanism came primarily from evaluations of interventions that reported differential responses to the intervention among some ethnicities, as in the case of non-Mexican and non-Puerto Rican women in an intervention targeting Latinos in Chicago [103] and non-Chinese and non-Filipino participants in an intervention for Asian and Pacific Islander gay men in San Francisco [113]. However, it is not possible to conclude that targeting the interventions using ethnicity as theorised in the ‘*specificity*’ mechanism would have been sufficient to generate a more even response across the diverse ethnicities of immigrants. The positive impact of this mechanism could also be inferred from an intervention in Israel targeting newly arrived Ethiopian immigrants, which was estimated to have reached 60% of these immigrants [123]. While it is clear that Ethiopian immigrants were specifically targeted because of their high rates of HIV and recent arrival in traumatic circumstances to Israel, it is difficult to conclude if the significant reach reported in this study was due to the targeting of Ethiopian immigrants (and therefore ‘*specificity*’) or due to the resources assigned to the task by public health officials in Israel (and therefore programme implementation) [123]. An intervention in the UK targeted African-born immigrants that initiated a soccer tournament modelled on the African Nations Cup was reported to have reached a wide diversity of African-born young men [105]. It could be inferred that drawing on African nations in this way utilised ethnicity – and therefore ‘*specificity*’ – as an effective way to reach diverse ethnicities of African-born immigrants. Evidence of resistance to this mechanism could be inferred from interventions where the lack of ‘*specificity*’ – where the heterogeneity of ethnicities in the target population (e.g., Africans) was not acknowledged – was a major limitation of two interventions with African-born immigrants [105,163]. The views studies pointed more clearly to a delicate balance between a resistance that failed to acknowledge differences (e.g., “Puerto Ricans are like all other Latinos”) or a related resistance of stereotyping around ethnicity (e.g., “all Puerto Ricans are the same”). In a similar vein, African-born HIV-positive people in the UK reported valuing both pan-African social interactions for the shared difference (not being British) while simultaneously desiring ‘*specificity*’ that acknowledged the reality of diverse ethnicities among African communities in the UK [143].

##### Context

The strongest evidence to support this theorised mechanism came from the intervention studies with moderate evidence for the role of this mechanism from the views studies. This may be an artefact of the higher number of interventions that reported using ethnicity to target their intervention activities. Fewer views studies were framed in terms of ethnicity – choosing instead to research broader samples of immigrants (e.g., Latinos) or multi-ethnic samples. Studies that emphasised the differences as well as the commonalities of ethnicities often positioned ethnicity alongside other factors such as socioeconomic background, experiences and reasons for migration, language and gender, [114,117,127,130,134,135,166,170] and sometimes reported the relevance of ethnicity as being related to levels of acculturation [130]. The degree to which commonalities and differences across ethnicities should be considered in the implementation of interventions is also important contextual information for this mechanism. African-born HIV-positive immigrants in the UK highlighted the impact of migration on ethnicity – where participants reported leaving Africa with an ethnicity (e.g., Ugandan) and arrived in the UK to be labelled as ‘African’ or ‘refugee’ or ‘asylum seeker’, which indicates these immigrants’ desire to maintain ethnicity after migration [143].

The moderate evidence from the intervention and views studies nonetheless points to the applicability of this theorised mechanism across primary HIV prevention and prevention efforts that seek to involve people living with HIV. The evidence to support this mechanism from the intervention and views studies was largely consistent with how the ‘*specificity*’ mechanism had originally been theorised. However, the evidence gave deeper insights into how ethnicity and therefore ‘*specificity*’ was highly related to context in the way that it was operationalised, and potentially resisted, as a mechanism for targeting behavioural HIV prevention.

##### Discussion of ‘specificity’ mechanisms

The moderate evidence for this mechanism is consistent with the theory of ethnic groups in the literature that sees them as being defined in a social context derived from “the ethnicity claimed by people themselves and the ethnicity attributed to them by others” ([171] p. 3). A related question raised by this theorised mechanism relates to the impact of ethnicity on health, or more particularly, is ethnicity an important determinant of HIV-related health among immigrants? From the literature, Fenton ([172] p. 181) argues that while ethnicity can be a source of motivation, it rarely is, nor does it usually constitute the principal framework of social organisation, nor is it the fundamental principle of action. The evidence from this review is broadly in line with this and suggests that interventions should situate ethnicity alongside other characteristics in immigrant target populations such as gender, sexual identity, age and socioeconomic backgrounds [57,87,88,109,155,156,173-175]. This can be interpreted as a limitation of this mechanism or, at the very least, the insufficiency of ethnicity or ‘*specificity*’ alone to address important variations across immigrant populations based on gender, sexual orientation and social class that are pertinent to HIV prevention with immigrants [88,93]. In considering these population variations, there is some evidence to suggest a greater weighting be placed on ethnicity among immigrants who are less acculturated in the destination country, though it is unclear if acculturation increases or decreases HIV-related risks [93,176,177].

A key question thrown up by this mechanism relates to the degree to which commonalities and differences across ethnicities should be considered in the implementation of interventions. In other words, for example, to what degree are Thai-born Australians similar to, but unlike, other Asian-born immigrants in terms of HIV prevention? People who share the same label in high-income countries – Latino, Asian, African – can be vastly different in terms of ethnicity and it has been recommended that these within-group differences in populations that are labelled in this way should not be ignored [57,177,178].

#### The evidence around ‘embeddedness’ mechanisms

##### Activity

**Figure 5:**
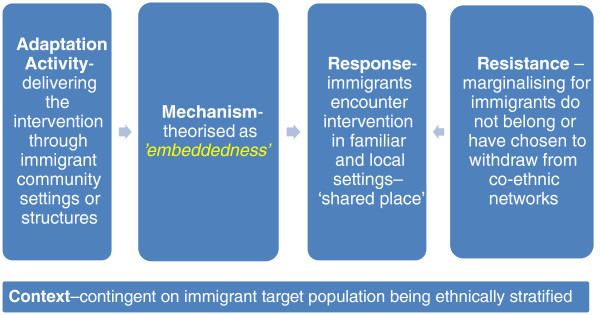
**‘*Embeddedness*’ mechanisms.**

The evidence from the intervention studies strongly supported the role of this mechanism – where behavioural HIV prevention interventions are ‘embedded’ in settings that are familiar to immigrants (Figure 5). Twenty-one interventions reported sound evidence, and a further eight interventions reported moderate evidence for this adaptive mechanism in contributing to culturally appropriate interventions. Interventions were often delivered through outreach to physical settings where immigrants gather including public places, community events, places of worship or recreation (e.g., cafes and bars) and even people’s homes [51,95-98,102,105,110,123,124,158,160,162-164]. The settings could also be community structures such as ethnic press and radio media [51,95,103,107,147,150,161], which was sometimes employed as a stand-alone strategy or as an adjunct to outreach to physical settings. Examples of partial evidence of this mechanism included interventions delivered in settings where the immigrants were there for another reason such as clinics [111] or refugee reception centres [159].

The evidence from views studies to support this mechanism was almost equally divided among sound, moderate, partial and no evidence, indicating that this mechanism was seen as less important by immigrants themselves in terms of culturally appropriate HIV prevention. Studies with sound or moderate evidence for ‘*embeddedness*’ usually reported that social networks were ethnically stratified with immigrants’ primary social interactions being with other co-ethnics [115,170] or other ethnicities related by language in the case of Latinos in the USA [131,148] or region of birth in the case of African-born immigrants in the UK [116,143,145]. These social networks were also stratified in other ways such as the ethnic stratification of gay male bars reported in three North American cities and the ethnic stratification of commercial sex work between Latino and Asian sex workers in the USA [130,142]. A potential for using faith-based settings was reported in two views studies among African-born HIV-positive immigrants in the UK [116,118], indicating a faith-based stratification might also be a way to reach some immigrant populations.

##### Response and resistance

The theorised response of immigrants to this mechanism is that they encounter the intervention in familiar and local settings around a notion of ‘shared place’. The intervention studies did not directly report the feedback of participants on the impact of this mechanism, though the reach of many interventions can be inferred, at least in part, to the use of community settings and structures. Certainly those planning and delivering the intervention attributed ‘*embeddednes*s’ as an integral part of the intervention. For example, there was a high reach reported in interventions that used community settings including an intervention with Ethiopian immigrants in Israel [123], with Latino gay men in San Francisco [97], with Spanish, Portuguese and Turkish immigrants in Switzerland [51] and with immigrant gay and bisexual farm workers in Texas and California [110]. Interventions that used community structures such as ethnic media reported increased HIV testing among target immigrant communities, though it was not possible to attribute this outcome to the media campaign [161]. Another intervention that was struggling to recruit gay Latino immigrants in California reported success after implementing social marketing strategies in Latino gay/bisexual publications [95]. There was no reported resistance to the ‘*embeddedness*’ of interventions and no resistances could be inferred from the studies in the review. The evidence for potential resistances to a settings approach came mainly from the views studies with partial or no evidence for the role of this mechanism. Many of these studies were among HIV-positive immigrants, some of whom reported purposively withdrawing from co-ethnic family and friendship networks because of actual or perceived breaches of confidentiality or high levels of HIV-related social stigma [114,118-121,127,133,144,146,147,166]. This isolation from friends and families – a potential resistance to an ‘*embeddedness*’ approach – was done out of perceived necessity, even though as one Latino HIV-positive person explains: “For those of us who are infected, you don’t know how important the family is for us: the family can help to cure without medicine even the most terrible physical pain” ([121] p. 439). Here, we can see that family is highly valued among immigrants even as these same HIV-positive people choose to isolate themselves from family and co-ethnic networks that might offer support and solace. The need for secrecy for HIV-positive immigrants tended to outweigh the retention of co-ethnic social networks in the event their HIV diagnosis was revealed or suspected in their close social interactions [120,140,144,166].

##### Context

The key context of this mechanism in interventions is that it is contingent on the social networks of the target immigrant population being ethnically and/or linguistically and/or racially stratified. For example, Moroccans in The Netherlands [158], Latinos in the US [122] and African-born immigrants in the UK [105] (where there was some evidence of a pan-African identity) were examples from interventions using settings included in the review where there was ethnic, linguistic and racial stratification, respectively. In addition, there was some evidence suggesting the utility of faith-based stratification of immigrant social networks and the use of faith-based settings for ‘embedding’ interventions. The evidence from intervention studies suggests that ‘*embeddedness*’ has most utility in terms of primary HIV prevention. The views studies indicated that HIV-positive immigrants often purposively withdrew from ethnic, linguistic and racial networks (preferring instead the anonymity of other networks) as a strategy to maintain secrecy around their HIV status and avoid potential or actual stigma associated with HIV in their ethnic community. Consequently, ‘*embeddedness*’ would seem to have little relevance in HIV prevention efforts that seek to engage immigrants living with HIV. Other marginalised groups of immigrants, such as gay men, injecting drug users and sex workers, may also purposively withdraw from co-ethnic networks, which suggests that primary prevention interventions seeking to use ‘*embeddeddness*’ with these groups of immigrants need to respond to this in the choice of settings for delivering the intervention (e.g., delivering the intervention at gay bars frequented by Latino gay men).

The evidence to support this mechanism from the intervention and views studies was largely consistent with how the ‘*embeddedness*’ mechanism had originally been theorised. However, the evidence gave deeper insights into how this mechanism was contingent on the stratification of immigrant communities to ‘embed’ HIV prevention interventions within familiar settings and structures.

##### Discussion of ‘embeddedness’ mechanisms

The moderate evidence from the intervention and views studies suggested that this mechanism could be regarded as another expression of ‘closeness’ found in ‘*authenticity*’ mechanisms (see Additional file 10), which tended to relate to interpersonal ‘closeness’ functioning at a broader social and community level. Evidence from the views studies implied that friends, families and co-ethnic communities were seen as valuable sources of support and interventions had to infiltrate these ‘ethnic worlds’ and social networks to effectively reach immigrant populations in HIV prevention. The wider literature of health promotion acknowledges the role of using settings that are familiar to the target group [52,153,178] and of using community structures such as ethnic media [52,176] to implement effective interventions with immigrants. The evidence from this review suggests that the contribution of this mechanism in HIV prevention interventions is contingent on the immigrant target group being stratified along ethnic, linguistic, racial or religious lines. Where there is no ‘ethnic world’ to infiltrate – where the target immigrant population is socially dispersed with no unifying networks or connections – the utility of ‘*embeddedness*’ mechanisms is diminished. Similarly, the utility of this mechanism is diminished in HIV prevention efforts involving HIV-positive immigrants who are widely reported to withdraw from co-ethnic networks to maintain the ‘secret’ of their HIV status [145]. ‘*Embeddedness*’ mechanisms may be particularly relevant in contexts where there is evidence of disparities in terms of access by immigrants to HIV-related health services by implementing outreach programmes and ‘embedding’ interventions in physical and other settings that are more familiar to their daily lives.

##### Strengths and limitations of the review of evidence

At the outset this review of evidence sought to generate insights to answer a research question around ‘How are HIV prevention interventions with immigrants adapted to suit the cultural context?’ The review could have been more straightforward had we chosen to carry out a systematic review with explicit inclusion and exclusion criteria focussed on outcomes and study types. While the systematic searches would have been just as comprehensive (unless we employed filters for study types) it is likely that applying the kinds of criteria usually used in ‘conventional’ systematic reviews would have excluded virtually all of the intervention studies included in this review. Instead this review chose a path that was arguably more difficult for appraising the interventions to be included in the review. Somewhat paradoxically, the inclusion of intervention studies that in the eyes of some should be excluded as valid evidence actually increased the complexity of the review and increased the opportunities to glean insights from the evidence that was found. In addition, we chose to integrate the perspectives of immigrants themselves from qualitative studies and these studies did indeed contribute to critical understandings of how and why interventions worked (or not) for immigrants.

A key limitation of this realist review is that the inferences we have drawn from the primary studies are interpretive, and thus open to potentially different interpretation by other researchers. This is a limitation reported by others who have conducted realist reviews [67,69] and attempts are now underway to strengthen and provide guidance on quality assurance and more uniform reporting of realist reviews [71]. We have attempted to address this limitation by being as explicit as we can about the methods we followed and providing a breadth of information including data extraction templates in the additional files to this article so that others may see how we have arrived at our findings. We have also completed a PRISMA checklist (see Additional file 11) to assist others in assessing the reporting of this review.

In realist reviews the focus on the underlying theories of programmes (rather than the programmes themselves) means that a wider range of primary studies may be relevant to the analysis and synthesis. It is likely that since our searches were limited to the field of HIV/AIDS, other primary studies of interventions to improve the health and well-being of immigrants in high-income countries were missed. These studies may have shed light on our mechanisms and offered alternative conceptions of our mechanisms but it was beyond scope of this doctoral research to attempt to retrieve this evidence.

In our review we sought to unpack the mechanisms contributing to cultural appropriateness in HIV prevention with immigrants. In our initial scan of the literature we found seven candidate mechanisms and we chose to retain all seven mechanisms for further testing against the evidence found in systematic searches. This decision means that our review covered a breadth of activities, mechanisms and contexts that can be difficult to summarise except in very broad ways and therefore our findings may lack sufficient detail to some potential users of the review.

In our review separate searches were carried out for intervention studies and for views studies. These systematic searches across four public health databases resulted in eight sets of records that needed to be appraised for relevance and rigour. As the lead investigator carried out this appraisal process there was a strong sense that there was a significant crossover and duplication between the search records for interventions and the search records for views studies retrieved from each database. Therefore we would urge others undertaking a review of this kind to consider expanding the search terms and carrying out a single search on each database for intervention and views studies. The search records retrieved could then be progressively appraised for relevance and rigour by title, abstract and full report. During the appraisal process studies could be categorised as either intervention or views studies. This could potentially reduce ‘double-handling’ of search records while not compromising on the comprehensiveness of the search strategies.

Another difficulty in appraising the evidence, which is perhaps peculiar to this review, was that we often needed to access the full report to determine whether the study did in fact relate to immigrants and/or relate to immigrants from developing and middle-income countries. Studies frequently referred to ‘ethnic minorities’, ‘racial minorities’, ‘Latinos’, ‘Africans’ and ‘Asians’, and it was only after accessing the full report that we could assess whether these terms actually referred to immigrants. This peculiarity probably stems in large part from the lack of consistency in the language used around ethnicity and race across high-income countries. For example, what might be called racial conflict in the USA is likely to be called ethnic conflict in countries of the former Yugoslavia.

Contextual information in terms of intervention participant characteristics or implementation environments is highly valued in a realist review as mechanisms may fire or misfire dependent on these contexts [66]. This review was able to retrieve information on the contexts of intervention participants, largely through the inclusion of views studies. The review was less able to attend to the implementation environments as this information was often not available in the studies, and the span of high-income countries in which the intervention and views studies were carried out made it difficult to infer this contextual information. Future research may need to explore these implementation environments in more detail.

## Conclusion

Today, population mobility and migration are unprecedented in volume, speed and reach [36], and immigrants from developing and middle-income countries have emerged as populations significantly affected by HIV in many high-income countries in Europe, North America and Australasia. The vulnerabilities of immigrants living in high-income countries to the negative impacts of HIV include social exclusion along with socioeconomic, cultural and language barriers to HIV prevention [5,7]. Indeed there is emerging evidence of inequalities being experienced by immigrants from developing and middle-income countries living in high-income countries in relation to HIV.

HIV prevention in high-income countries has generally included whole-of-population approaches and targeted approaches that take into account the shared characteristics of the members of a sub-population. It is generally accepted that prevention interventions will be more effective if they are culturally appropriate to the population they serve. However the strategies used to achieve cultural appropriateness in behavioural HIV prevention with immigrants vary widely and the underlying mechanisms of these strategies have rarely been examined.

This review of evidence explored targeted approaches in behavioural HIV prevention interventions with immigrants in high-income countries. In particular, the research generated insights on the adaptive mechanisms that contribute to culturally appropriate HIV prevention. Two types of studies contributed to the review: studies of interventions and qualitative studies of immigrants’ views of HIV prevention. In this way the review of evidence brought together ‘expert’ and ‘lay’ perspectives to the analysis and synthesis. The rationale to frame this review across diverse immigrant populations is not to diminish the important differences between these populations but rather to generate insights from the commonalities that exist to allow potential users of this review to apply the findings with multiple immigrant communities.

Overall, the review suggests that adaptations to targeted HIV prevention interventions with immigrants are feasible. The review suggests that the suite of adaptive mechanisms we have mapped – ‘*authenticity*’, ‘*understanding*’, ‘*consonance*’, ‘*specificity*’, ‘*embeddedness*’, ‘*endorsement*’ and ‘*framing*’ – do contribute to cultural appropriateness in HIV prevention with immigrants and are potentially transferable to multiple immigrant populations in high-income countries. Our review suggests that adopting any of these mechanisms in interventions requires a careful appraisal of contextual issues in terms of population characteristics and implementation environments.

The strongest evidence supported the role of ‘*consonance*’ mechanisms, indicating the pivotal need to incorporate elements of cultural values into the intervention content. Moderate evidence was found to support the role of three other mechanisms – ‘*understanding*’, ‘*specificity*’ and ‘*embeddedness*’ – which indicated that using the language of immigrants – usually the ‘mother tongue’, targeting (in terms of ethnicity) and the use of settings were also critical elements in culturally appropriate HIV prevention. There was mixed evidence for the roles of ‘*authenticity*’ and ‘*framing*’ mechanisms, which suggests that staffing the intervention with immigrants and priority setting with immigrant community institutions encompass less critical mechanisms. Only partial evidence was found to support role of ‘*endorsement*’ mechanisms, indicating that gaining immigrant community endorsement was the least critical mechanism in culturally appropriate behavioural HIV prevention.

HIV prevention in high-income countries is often framed in terms of priority populations such as gay men/men who have sex with men, people living with HIV, people who inject drugs, sex workers, and (more recently) heterosexual men and women. The evidence from this review suggests that, after taking contextual issues into consideration, the optimal locations for interventions with immigrant gay men/men who have sex with men are around ‘*consonance*’, ‘*understanding*’, ‘*specificity*’, ‘*embeddedness*’ mechanisms and to a lesser extent ‘*authenticity*’ and ‘*framing*’. The optimal locations for interventions with heterosexual men and women are the same, with the addition of a limited role for ‘*endorsement*’ mechanisms. The review suggests that interventions that primarily seek to engage immigrants living with HIV should focus on consonance ‘*understanding*’, ‘*specificity*’ mechanisms and to a lesser extent ‘*framing*’. This review is not able to give guidance for interventions with immigrants who inject drugs or immigrant sex workers because of the very low number of primary studies with these sub-populations included in the review.

The adaptive mechanisms outlined in this review should be seen as the key, rather than the only, mechanisms contributing to cultural appropriateness, and the review found evidence that they were interrelated rather than mutually exclusive mechanisms. Further research is needed to examine the relationships among these seven mechanisms and any impacts they contribute to the effectiveness of interventions and HIV-related health outcomes among immigrants.

This review found that behavioural HIV prevention interventions with immigrants in high-income countries are relatively underdeveloped. The review has contributed to knowledge of the mechanisms that operate in these interventions, which are at an early stage of policy development [78]. Thus, to paraphrase Pawson et al. [68], the progress made in our realist review of evidence is not from ignorance to answer, but from some knowledge to more knowledge, of the suite of mechanisms that underpin culturally appropriate HIV prevention for immigrants. This knowledge can contribute to addressing the vulnerabilities and negative impacts of HIV for immigrants from developing and middle-income countries living in high-income countries in a context of accelerating mass movements of people in a globalised world.

## Competing interests

The authors declare that they have no competing interests.

## Authors’ contributions

TMM had the original research idea, which was refined by PW. PW guided the methodology and methods and provided advice on all stages of the review process. TMM carried out the searches for primary studies and screened them for inclusion. TMM also carried out the data extraction and wrote the initial unpublished report on which this manuscript is based with guidance and support from PW. TMM and PW contributed to the drafting and structure of this manuscript and both authors have read and approved the final manuscript.

## Supplementary Material

Additional file 1 ‘Known set’ of intervention studies with controlled vocabulary terms. Click here for file

Additional file 2 ‘Known set’ of views studies with controlled vocabulary terms. Click here for file

Additional file 3 Results: generating candidate mechanisms. Click here for file

Additional file 4 Collation of intervention studies included in the review. Click here for file

Additional file 5 **Collation of views studies included in the review.** (PDF 342 kb) Click here for file

Additional file 6 Annotation of intervention studies: Summary against adaptive mechanisms. Click here for file

Additional file 7 Annotation of views studies: Summary against adaptive mechanisms. Click here for file

Additional file 8 Annotation process for primary studies included in the analysis and synthesis. Click here for file

Additional file 9 PRISMA flow diagram. Click here for file

Additional file 10 Supplementary results: evidence synthesis and discussion. Click here for file

Additional file 11 PRISMA checklist for reporting of systematic reviews. Click here for file
