# Supplementary material for: HIV among immigrants living in high-income countries: a realist review of evidence to guide targeted approaches to behavioural HIV prevention
Source: Syst Rev. 2012 Nov 20;1:56. doi: 10.1186/2046-4053-1-56 (PMC3534573; doi:10.1186/2046-4053-1-56)
Supplement: Additional file 1 — ‘Known set’ of intervention studies with controlled vocabulary terms. [file 2046-4053-1-56-S1.pdf]

‘Known set’ of intervention studies with controlled vocabulary terms

Article	PubMed	Psych Info	CINAHL	Sociological abstracts/ ERIC/PAIS/ Social Services Abstracts
**1. Peragallo, N DeForge, B O’Campo, P Mi Lee, S Ju Kim, Y Cianelli, R Ferrer, L.** (2005). *A randomized clinical trial of an HIV-risk-reduction intervention among low-income Latina women.* Nursing Research, 54 (2), 108-118.	MH - Acculturation<br>MH - Adolescent<br>MH - Adult<br>MH - Female<br>MH - HIV Infections/*prevention & control<br>MH - *Health Education<br>MH - *Hispanic Americans<br>MH - Humans<br>MH - *Mexican Americans<br>MH - Poverty<br>MH - *Risk Reduction Behavior<br>MH - *Sexual Behavior<br>MH - United States	Subject headings:<br>*AIDS Prevention<br>*Hispanics<br>*HIV<br>*Human Females<br>*Sexual Risk Taking<br>Intervention<br>Lower Income Level<br>Urban Environments<br><br>Key concepts<br>low-income Latina Women, HIV risk reduction intervention, high-HIV-risk sexual behaviors, urban areas	Adult<br>Bandura's Social Cognitive Theory<br>Bivariate Statistics<br>Chi Square Test<br>Clinical Trials<br>Coefficient Alpha<br>Condoms / ut [Utilization]<br>Convenience Sample<br>Cultural Sensitivity<br>Data Analysis Software<br>Descriptive Statistics<br>Effect Size<br>Female<br>Funding Source<br>*HIV Education<br>*HIV Infections / pc [Prevention and Control]<br>Health Knowledge / ev [Evaluation]<br>*Hispanics<br>Illinois<br>Interviews<br>Mexico<br>Multivariate Analysis<br>Poverty<br>Professional Practice, Theory-Based<br>Puerto Rico<br>Random Assignment<br>Regression<br>Repeated Measures<br>Research Subject Recruitment<br>*Risk Taking Behavior / pc [Prevention and Control]<br>Safe Sex<br>Sample Size<br>Scales<br>Teaching Methods<br>*Transcultural CareUrban Areas	not found

**2. Kocken, P Voorham, T Brandsma, J Swart, W. (2001).** *Effects of peer-led AIDS education aimed at Turkish and Moroccan male immigrants in The Netherlands.* European Journal of Public Health, 11(2), 153-159.	MH - Acquired Immunodeficiency Syndrome/*prevention & control/transmission<br>MH - Adolescent<br>MH - Adult<br>MH - Condoms/utilization<br>MH - Disease Transmission, Horizontal/prevention & control<br>MH - Educational Status<br>MH - Health Education/*methods<br>MH - *Health Knowledge, Attitudes, Practice<br>MH - Humans<br>MH - Logistic Models<br>MH - Male<br>MH - Middle Aged<br>MH - Morocco/ethnology<br>MH - Multivariate Analysis<br>MH - Netherlands/epidemiology<br>MH - *Peer Group<br>MH - Program Evaluation<br>MH - Questionnaires<br>MH - Risk Assessment<br>MH - Sexual Behavior/ethnology<br>MH - Socioeconomic Factors<br>MH - Transients and Migrants/*statistics & numerical data<br>MH - Turkey/ethnology	Subject Headings:<br>*AIDS Prevention<br>*Health Education<br>*Immigration<br>*Peers<br>Condoms<br>Family Planning Attitudes<br>Health Attitudes<br>Human Males<br>Program Evaluation<br><br>Key concepts<br>AIDS education, immigrant men, Turkish immigrants, Moroccan immigrants, Netherlands, perceived AIDS threat, condom use beliefs	not found	not found
**3.McQuiston, C Flaskerud, JH (2003)** *"If they don't ask about condoms, I just tell them": A descriptive case study of Latino lay health advisers' helping activities.* Health Education and Behavior, 30 (1), 79-96.	MH - Adolescent<br>MH - Adult<br>MH - Attitude to Health/*ethnology<br>MH - Communication Barriers<br>MH - *Community Health Aides<br>MH - Condoms/*utilization<br>MH - Female<br>MH - HIV Infections/ethnology/*prevention & control<br>MH - Health Education/*manpower/organization & administration<br>MH - Health Promotion/*manpower/organization & administration<br>MH - Humans<br>MH - Male	Subject Headings:<br><br>*AIDS Prevention<br>*Educational Programs<br>*Health Education<br>*Hispanics<br>*HIV<br><br>Key concepts<br>lay health adviser program, prevention, HIV, AIDS, Mexican Americans	Acculturation<br>Adult<br>Audiorecording<br>Case Studies<br>Community Health Services<br>Community Health Services / ev [Evaluation]<br>Condoms<br>Content Analysis<br>Control (Psychology)<br>Cultural Sensitivity<br>Descriptive Research<br>Descriptive Statistics<br>Ethnography<br>Evaluation Research<br>Female<br>Fieldwork<br>Funding Source	not found

	MH - Mexican Americans/*education/psychology<br>MH - North Carolina<br>MH - Organizational Case Studies<br>MH - Program Evaluation<br>MH - Qualitative Research<br>MH - Sexual Behavior/*ethnology<br>MH - Sexually Transmitted Diseases/ethnology/*prevention & control<br>MH - Social Support<br>MH - Vulnerable Populations		HIV Education<br>*HIV Infections / eh [Ethnology]<br>*HIV Infections / pc [Prevention and Control]<br>Health Education<br>Health Promotion<br>Hispanics<br>Immigrants<br>Male<br>Medically Underserved<br>North Carolina<br>Participant Observation<br>Professional Role<br>Program Evaluation<br>Questionnaires<br>Referral and Consultation<br>Semi-Structured Interview<br>Summated Rating Scaling<br>Support, Psychosocial<br>Volunteer Workers / ed [Education]<br>*Volunteer Workers	
**4. Loue, S Lloyd, LS Phoombour, E. (1996).** *Organizing Asian Pacific Islanders in an urban community to reduce HIV risk: A case study.* AIDS Education and Prevention, 8(5), 381-393.	MH - Acquired Immunodeficiency Syndrome/ethnology/*prevention & control<br>MH - Asia, Southeastern/ethnology<br>MH - *Asian Americans<br>MH - California<br>MH - Community Networks/*organization & administration<br>MH - Community-Institutional Relations<br>MH - Consumer Participation/*methods<br>MH - Far East/ethnology<br>MH - Humans<br>MH - Interinstitutional Relations<br>MH - Longitudinal Studies<br>MH - Organizations, Nonprofit/*organization & administration<br>MH - Pacific Islands/ethnology<br>MH - Program Evaluation<br>MH - *Social Planning	not found	*Asians<br>California<br>*Communities<br>Community Assessment<br>Community Networks<br>Community Role<br>Focus Groups<br>*HIV Education<br>*HIV Infections / pc [Prevention and Control]<br>*Program Development<br>Questionnaires<br>Urban Areas	*Acquired Immune Deficiency Syndrome; *Health Education; *Community Organizations; *Oceanic Cultural Groups; *Program Implementation; *Program Evaluation; San Diego, California

**5. Worth, H Denholm, N Bannister, J. (2003).** *HIV/AIDS and the African refugee education program in New Zealand.* AIDS Education and Prevention, 15(4), 346-356.	MH - Adult<br>MH - Africa/ethnology<br>MH - Attitude to Health/*ethnology<br>MH - Community Health Services/*organization & administration<br>MH - Cultural Characteristics<br>MH - Emigration and Immigration<br>MH - Female<br>MH - HIV Seropositivity/*ethnology/*psychology/therapy<br>MH - Health Education/methods/*organization & administration<br>MH - Health Promotion/methods/organization & administration<br>MH - Heterosexuality/ethnology<br>MH - Humans<br>MH - Male<br>MH - New Zealand<br>MH - Qualitative Research<br>MH - Refugees/education/*psychology<br>MH - Safe Sex/ethnology<br>MH - Social Isolation<br>MH - Social Support	Subject Headings:<br>*AIDS<br>*AIDS Prevention<br>*Health Education<br>*HIV<br>*Refugees<br>Blacks<br><br>Key concepts<br>human immunodeficiency virus, acquired immune deficiency syndrome, education program, HIV positive refugees, National HIV/AIDS Refugee Health Education Program	Adult<br>Africa<br>*Attitude to Health / eh [Ethnology]<br>*Community Health Services / am [Administration]<br>Culture<br>Female<br>HIV Seropositivity / th [Therapy]<br>*HIV Seropositivity / eh [Ethnology]<br>Health Education / mt [Methods]<br>*Health Education / am [Administration]<br>Health Promotion / am [Administration]<br>Health Promotion / mt [Methods]<br>Heterosexuality / eh [Ethnology]<br>Interpersonal Relations<br>Male<br>Narratives<br>New Zealand<br>Qualitative Studies<br>Refugees / ed [Education]<br>*Refugees / pf [Psychosocial Factors]<br>Safe Sex / eh [Ethnology]<br>Social Isolation<br>Support, Psychosocial	*Acquired Immune Deficiency Syndrome; *New Zealand; *African Cultural Groups; *Refugees; *Health Education; Relocation; Public Health
**6. Martijn, C de Vries, NK Voorham, T Brandsma, J Medi, M Hospers, HJ. (2004).** *The effects of AIDS prevention programs by lay health advisors for migrants in The Netherlands.* Patient Education and Counselling, 53, 157-165.	MH - Acquired Immunodeficiency Syndrome/*prevention & control<br>MH - Adult<br>MH - Attitude to Health/ethnology<br>MH - Community Health Aides/*organization & administration<br>MH - Condoms/utilization<br>MH - Female<br>MH - Health Education/*organization & administration<br>MH - Health Knowledge, Attitudes, Practice<br>MH - Humans<br>MH - Iraq/ethnology<br>MH - Male<br>MH - Morocco/ethnology<br>MH - Motivation<br>MH - Needs Assessment<br>MH - Netherlands<br>MH - Professional Competence/standards<br>MH - Program Evaluation<br>MH - Questionnaires<br>MH - Safe Sex	Subject Headings:<br>*AIDS<br>*AIDS Prevention<br>*Health Attitudes<br>*Immigration<br>*Program Evaluation<br>Health Promotion<br><br>Key concepts<br>lay health advisors, AIDS prevention programs, Turkish & Moroccan migrants, program effectiveness, acquired immunodeficiency syndrome, program evaluation, professional health advisors, attitudes	*Acquired Immunodeficiency Syndrome / pc [Prevention and Control]<br>Adult<br>Analysis of Variance<br>Condoms / ut [Utilization]<br>Correlation Coefficient<br>Descriptive Statistics<br>Female<br>*HIV Education / mt [Methods]<br>Health Knowledge<br>*Health Personnel<br>*Immigrants<br>Iraq / eh [Ethnology]<br>Male<br>Middle Age<br>Morocco / eh [Ethnology]<br>Netherlands<br>Pretest-Posttest Design<br>Program Evaluation<br>Questionnaires<br>Regression<br>Summated Rating Scaling	not found

	MH - Sex Education/*organization & administration<br>MH - Transients and Migrants/*education/psychology<br>MH - Turkey/ethnology		Turkey / eh [Ethnology]	
**7. Dushay, RA Singer, M Weeks, MR Rohena, L Gruber, R. (2001).** *Lowering HIV risk among ethnic minority drug users: comparing culturally targeted intervention to a standard intervention.* American Journal of Drug and Alcohol Abuse, 27(3) 501-524.	MH - Acquired Immunodeficiency Syndrome/*prevention & control<br>MH - Adult<br>MH - African Americans/psychology<br>MH - Analysis of Variance<br>MH - Cultural Characteristics<br>MH - Female<br>MH - Health Education<br>MH - Hispanic Americans/psychology<br>MH - Humans<br>MH - Intervention Studies<br>MH - Male<br>MH - Middle Aged<br>MH - Minority Groups/*psychology<br>MH - Patient Dropouts/psychology<br>MH - Puerto Rico<br>MH - Risk-Taking<br>MH - Self Concept<br>MH - Self Efficacy<br>MH - Substance Abuse, Intravenous/*psycho	Subject Headings:<br>*Early Intervention<br>*HIV<br>*Intravenous Drug Usage<br>*Minority Groups<br>*Sexual Risk Taking<br>Drug Therapy<br>Prevention<br><br>Key concepts<br>culturally competent interventions, drug treatment, HIV risk, injection drug use, prevention, ethnic minorities		*Acquired Immune Deficiency Syndrome; *Risk; *Drug Abuse; *Prevention; *Intervention; *Black Americans; *Puerto Rican Americans; *Cultural Sensitivity; Connecticut
**8. Operario, D Nemoto, T Syed, J Mazarei, M. (2005).** *Conducting HIV interventions for Asian Pacific Islander men who have sex with men: Challenges and compromises in community collaborative research.* AIDS Education and Prevention, 17(4), 334-346.	MH - Adolescent<br>MH - Adult<br>MH - *Asian Americans<br>MH - California<br>MH - Community Health Centers/*organization & administration<br>MH - *Cooperative Behavior<br>MH - HIV Infections/*prevention & control<br>MH - *Homosexuality, Male<br>MH - Humans<br>MH - Male<br>MH - *Oceanic Ancestry Group<br>MH - Organizational Case Studies<br>MH - Organizational Culture	Subject Headings:<br>*AIDS Prevention<br>*Communities<br>*HIV<br>*Intervention<br>*Organizations<br>Asians<br>Cooperation<br>Experimentation<br>Human Males<br>Pacific Islanders<br>Key concepts<br>HIV prevention intervention, community based organization, Asian & Pacific Islander men, community collaborative research		*Acquired Immune Deficiency Syndrome; *Intervention; *Asia; *Males; *Sexual Behavior; *Community Services; *Evaluation Research

**9. Soskolne, V Shtarkshall, RA. (2002).** *Migration and HIV prevention programmes: linking structural factors, culture, and individual behaviour-an Israeli experience.* Social Science & Medicine, 55, 1297-1307.	MH - Adolescent<br>MH - Adult<br>MH - Attitude to Health/*ethnology<br>MH - Communicable Disease Control/*organization & administration<br>MH - Culture<br>MH - *Emigration and Immigration<br>MH - Ethiopia/ethnology<br>MH - HIV Infections/*ethnology/*prevention & control<br>MH - Humans<br>MH - Israel/epidemiology<br>MH - Middle Aged<br>MH - Power (Psychology)<br>MH - Preventive Health Services/*organization & administration<br>MH - Psychosocial Deprivation<br>MH - Risk-Taking<br>MH - Sexually Transmitted Diseases/*ethnology/*prevention & control<br>MH - Social Class<br>MH - USSR/ethnology	Subject Headings:<br>*AIDS Prevention<br>*Cross Cultural Differences<br>*HIV<br>*Immigration<br>*Sociocultural Factors<br><br>Key concepts<br>migration, HIV prevention programs, structural factors, culture, individual behaviors, former Soviet Union immigrants, Ethiopian immigrants	Culture<br>*Emigration and Immigration<br>*HIV Infections / pc [Prevention and Control]<br>Health Policy<br>Health Services Accessibility<br>Israel	*Migration; *Immigrants; *Acquired Immune Deficiency Syndrome; *Prevention; *Risk Factors; *Israel; Slavic Cultural Groups; African Cultural Groups
**10. Amaro, HA Raj, A Reed, E Cranston, K (2002).** *Implementation and long-term outcomes of two HIV intervention programs for Latinas.* Health Promotion Practice, 3(2), 245-254.	not found	not found	Adult<br>Collaboration<br>Community Health Services<br>Convenience Sample<br>Cultural Competence<br>Curriculum<br>Female<br>*HIV Education / mt [Methods]<br>HIV Infections / rf [Risk Factors]<br>*HIV Infections / eh [Ethnology]<br>*HIV Infections / pc [Prevention and Control]<br>*Hispanics<br>Intervention Trials<br>Interviews<br>Logistic Regression<br>*Outcomes (Health Care)<br>Patient Satisfaction<br>Process Assessment (Health Care)<br>*Program Implementation<br>Prospective Studies<br>Public Health Administration<br>Questionnaires<br>*Women's Health	not found

**11. Organista, KC Carrillo, H Ayala, G (2004).** *HIV prevention with Mexican migrants.* Journal of Acquired Immune Deficiency Syndrome, 37 (Suppl 4) S227-S239.	MH - Acculturation<br>MH - Condoms/utilization<br>MH - Female<br>MH - HIV Infections/*prevention & control<br>MH - Homosexuality, Male<br>MH - Humans<br>MH - Male<br>MH - Mexico/ethnology<br>MH - Prostitution<br>MH - Risk Factors<br>MH - Risk-Taking<br>MH - Sexual Partners<br>MH - Social Class<br>MH - *Transients and Migrants<br>MH - United States	not found		not found
**12. Wong, FY Lye Chng, C Lo, W. (1998).** *A profile of six community-based HIV prevention programs targeting Asian and Pacific Islander Americans.* AIDS Education and Prevention, 10 (Suppl A), 61-76.	MH - *Asian Americans<br>MH - *Community Health Services<br>MH - HIV Infections/*prevention & control<br>MH - Humans<br>MH - Male<br>MH - United States	Subject Headings:<br>*AIDS Prevention<br>*Community Services<br>*HIV<br>*Male Homosexuality<br>*Program Evaluation<br>Asians<br>Key concepts:<br>evaluation of community-based HIV prevention programs, Asian & Pacific Islander American males how have sex with males	*Asians<br>*Community Health Services<br>Descriptive Statistics<br>*HIV Infections / pc [Prevention and Control]<br>Male<br>United States	*Oceanic Cultural Groups; *Asian Americans; *Acquired Immune Deficiency Syndrome; *Homosexual Relationships; *Males; *Community Organizations; *Bisexuality; *Health Care Services; Hawaii; Los Angeles, California; San Francisco, California; Philadelphia, Pennsylvania; New York City, New York; Boston, Massachusetts
**13. Haour-Knipe, M Fleury, F Dubois-Archer, F. (1999).** *HIV/AIDS prevention for migrants and ethnic minorities: three phases of evaluation.* Social Science and Medicine, 49, 1357-1372.	MH - Acquired Immunodeficiency Syndrome/*prevention & control<br>MH - HIV Infections/*prevention & control<br>MH - Humans<br>MH - *Minority Groups<br>MH - Program Evaluation/*methods<br>MH - Switzerland<br>MH - *Transients and Migrants	Subject Headings:<br>*AIDS Prevention<br>*Cultural Sensitivity<br>*Immigration<br>*Racial and Ethnic Groups<br>Minority Groups<br>Key concepts<br>evaluation of HIV/AIDS prevention program, migrants & ethnic minorities, Switzerland		*Acquired Immune Deficiency Syndrome; *Prevention; *Migrants; *Ethnic Groups; *Minority Groups; *Program Evaluation; *Switzerland; *Community Involvement; *Ethnic Neighborhoods

**14. McMahon, T Fairley, C Donovan, B Quin, J Wan, L.** *Effects of an ethnic media campaign on patterns of HIV testing among people from CALD backgrounds in Australia.* Sexual Health 2 (1), 2004.	MH - Adult<br>MH - Australia<br>MH - Communications Media<br>MH - Cultural Diversity<br>MH - Ethnic Groups/*statistics & numerical data<br>MH - Female<br>MH - HIV Infections/*diagnosis<br>MH - Health Education/methods/*statistics & numerical data<br>MH - Humans<br>MH - Language<br>MH - Male<br>MH - Mass Media<br>MH - Mass Screening/*statistics & numerical data<br>MH - Pilot Projects	not found		not found
**15. Vinh-Thomas, P Bunch, MM Card, JJ. (2003).** *A research-based tool for identifying and strengthening culturally competent and evaluation-ready HIV/AIDS prevention programs.* AIDS Education and Prevention, 15 (6), 481-498.	MH - *Cultural Diversity<br>MH - *Evidence-Based Medicine<br>MH - HIV Infections/*prevention & control<br>MH - Health Services Research<br>MH - Humans<br>MH - Preventive Health Services/*organization & administration/standards<br>MH - Program Development<br>MH - Program Evaluation<br>MH - *Self Efficacy<br>MH - United States	Subject Headings:<br>*AIDS Prevention<br>*Cultural Sensitivity<br>*Health Care Delivery<br>*Program Development<br>*Program Evaluation<br>Health Care Services<br>HIV<br>Minority Groups<br><br>Key concepts<br>HIV/AIDS prevention programs, health service delivery, cultural competence, minority populations, cultural sensitivity, racial disparities, evaluation readiness	*Cultural Competence<br>*HIV Infections / pc [Prevention and Control]<br>Health Services Research<br>Instrument Construction<br>Interrater Reliability<br>Interviews<br>*Medical Practice, Evidence-Based<br>Pretest-Posttest Design<br>Preventive Health Care / st [Standards]<br>*Preventive Health Care / am [Administration]<br>Program Development<br>*Program Evaluation<br>Questionnaires<br>Random Assignment<br>United States	*Acquired Immune Deficiency Syndrome; *Cultural Sensitivity; *Prevention; *Program Evaluation

**16. Hovey, JD Booker, V Seligman, LD. (2007).** *Using theatrical presentations as a means of disseminating knowledge of HIV/AIDS risk factors to migrant farmworkers: An evaluation of the effectiveness of the Informate program.* Journal of Immigrant Health, 9, 147-156.	MH - Adolescent<br>MH - Adult<br>MH - *Agriculture<br>MH - California<br>MH - Child<br>MH - *Drama<br>MH - Female<br>MH - HIV Infections/*prevention & control/transmission<br>MH - *Health Knowledge, Attitudes, Practice<br>MH - Humans<br>MH - Male<br>MH - Program Evaluation<br>MH - Questionnaires<br>MH - Risk Factors<br>MH - *Transients and Migrants	not found		not found
**17. Kaplan, EH Soskolne, V Adler, B Leventhal, A Shtarkshall RA. (2002).** *A model-based evaluation of a cultural mediator outreach program for HIV positive Ethiopian immigrants in Israel.* Evaluation Review, 26(4), 382-394.	MH - *Community-Institutional Relations<br>MH - *Emigration and Immigration<br>MH - Ethiopia/ethnology<br>MH - Female<br>MH - HIV Infections/ethnology/*prevention & control/transmission<br>MH - Humans<br>MH - Israel/epidemiology<br>MH - Male<br>MH - Models, Statistical<br>MH - Pregnancy<br>MH - Pregnancy Complications, Infectious/ethnology/prevention & control<br>MH - Program Evaluation/*methods<br>MH - Safe Sex/*ethnology	not found		(ERIC)<br>Acquired Immune Deficiency Syndrome; Cultural Awareness; Cultural Differences; Foreign Countries; Health Education; Immigrants; Models; Outreach Programs; Program Evaluation; Sexuality

**18. Carballo-Dieguez, A Dolezal, C Leu, CS Nieves, L Diaz F Decena, C Balan I (2005).** *A randomized controlled trial to test an HIV-prevention intervention for Latino gay and bisexual men: lessons learned.* AIDS Care. 17(3), 314-28.	MH - Adolescent<br>MH - Adult<br>MH - Aged<br>MH - Bisexuality/ethnology/*psychology<br>MH - Female<br>MH - HIV Infections/ethnology/*prevention & control<br>MH - Hispanic Americans/*psychology<br>MH - Homosexuality, Male/ethnology/*psychology<br>MH - Humans<br>MH - Male<br>MH - Middle Aged<br>MH - New York City/ethnology<br>MH - Power (Psychology)<br>MH - Unsafe Sex/ethnology/*prevention & control/psychology<br>MH - Urban Health	Subject Headings:<br>*AIDS Prevention<br>*Bisexuality<br>*Hispanics<br>*Male Homosexuality<br>*Safe Sex<br>Empowerment<br>Intervention<br><br>Key concepts<br>Latino gay men, Latino bisexual men, HIV prevention intervention, unsafe sex reduction, empowerment theory	Adult<br>Anal Intercourse<br>*Bisexuality<br>Clinical Assessment Tools<br>Descriptive Statistics<br>*Empowerment<br>Funding Source<br>*Health Behavior<br>*Hispanics<br>*Homosexuality<br>Intervention Trials<br>Interviews<br>Male<br>Men's Health<br>Middle Age<br>New York<br>Prospective Studies<br>Questionnaires<br>Random Assignment<br>*Safe Sex<br>Secondary Analysis<br>Self Report<br>Translations	*Prevention; *Risk; *Males; *Acquired Immune Deficiency Syndrome; *Homosexuality; *Bisexuality; *Sexual Behavior; *Hispanic Americans
**19. Choi, KH Lew, S Vittinghoff, E Cattania, JA Barrett, DC Coates DJ (1996).** *The efficacy of brief group counseling in HIV risk reduction among homosexual Asian and Pacific Islander men.* AIDS. 10(1), 81-7.	MH - Adult<br>MH - *Asian Americans<br>MH - *Counseling<br>MH - HIV Infections/*ethnology/*prevention & control<br>MH - *Homosexuality, Male<br>MH - Humans<br>MH - Male<br>MH - Pacific Islands/ethnology<br>MH - Risk Factors<br>MH - San Francisco<br>MH - Self Concept<br>MH - Sex Education<br>MH - Sexual Behavior<br>MH - Sexual Partners	Subject Headings:<br>*AIDS Prevention<br>*Asians<br>*Cultural Sensitivity<br>*Group Counseling<br>*Male Homosexuality<br>Health Attitudes<br>Health Knowledge<br>HIV<br>Risk Taking<br>Self Concept<br>Social Support<br>Key concepts<br>culturally appropriate self identity & social support & safe sex & HIV knowledge & attitudes & risk reduction, HIV positive vs negative homosexual male Asians & Pacific Islanders		not found

**20. Nyamathi, AM, Flaskerud, J Bennett, C Leake, B Lewis C. (1994).** *Evaluation of two AIDS education programs for impoverished Latina women.* AIDS Education and Prevention 6(4), 296-309.	MH - Acquired Immunodeficiency Syndrome/*prevention & control/psychology/transmission<br>MH - Adolescent<br>MH - Adult<br>MH - Aged<br>MH - Female<br>MH - Follow-Up Studies<br>MH - *Health Education<br>MH - Health Knowledge, Attitudes, Practice<br>MH - Hispanic Americans/*education/psychology<br>MH - Homeless Persons/*education/psychology<br>MH - Humans<br>MH - Los Angeles<br>MH - Middle Aged<br>MH - *Poverty<br>MH - *Program Evaluation<br>MH - Social Support<br>MH - *Urban Population	Subject Headings:<br>*AIDS Prevention<br>*Health Education<br>*Hispanics<br>*Human Females<br>*Poverty<br>Drug Addiction<br>Homeless<br><br>Key concepts<br>culturally sensitive AIDS education programs, impoverished homeless or drug addicted Latina female 18-75 yr olds	*Acquired Immunodeficiency Syndrome / pc [Prevention and Control]<br>Adolescence<br>Adult<br>Analysis of Variance<br>California<br>Content Validity<br>Female<br>*Health Education<br>*Hispanics<br>*Homeless Persons / ed [Education]<br>Lazarus Theory of Stress and Coping<br>Loglinear Models<br>Middle Age<br>*Poverty<br>*Program Evaluation<br>Quasi-Experimental Studies<br>Questionnaires<br>Random Sample<br>Repeated Measures<br>Research Instruments<br>Scales<br>Substance Dependence<br>Urban Areas	*Acquired Immune Deficiency Syndrome; *Educational Programs; *Program Evaluation; *Latin American Cultural Groups; *Low Income Groups; *Womens Health Care; *Homelessness; *Drug Addiction; *Los Angeles, California
**TOTAL** (found on each database)	**19**	**14**	**11**	**11**
