# Supplementary material for: HIV among immigrants living in high-income countries: a realist review of evidence to guide targeted approaches to behavioural HIV prevention
Source: Syst Rev. 2012 Nov 20;1:56. doi: 10.1186/2046-4053-1-56 (PMC3534573; doi:10.1186/2046-4053-1-56)
Supplement: Additional file 2 — ‘Known set’ of views studies with controlled vocabulary terms. [file 2046-4053-1-56-S2.pdf]

'Known set' of views studies with controlled vocabulary terms

Article	PubMed	Psych Info	CINAHL	Sociological abstracts/ Social ServicesAbstracts/ PAIS /ERIC
**1. Anderson, J and Doyal, L (2004).** *Women from Africa living with HIV in London: a descriptive study.* AIDS Care, 16 (1), 95-105.	MH - Adult<br>MH - Africa/ethnology<br>MH - African Continental Ancestry Group/ ethnology<br>MH - Emigration and Immigration<br>MH - Female<br>MH - HIV Infections/ethnology/ *psychology<br>MH - Humans<br>MH - London/epidemiology<br>MH - Middle Aged<br>MH - Needs Assessment<br>MH - Patient Acceptance of Health Care/ psychology<br>MH - Prejudice<br>MH - Truth Disclosure	Subject Headings: *HIV<br>*Hospitals<br>*Patient History<br>*Public Health Services<br>Human Females<br>Patients<br>Key concepts:<br>Human Immunodeficiency Virus, HIV-positive African women, London hospitals, illness biographies, life history	Subject headings:<br>Adult<br>Africa<br>Blacks<br>Descriptive Research<br>Descriptive Statistics<br>Emigration and Immigration<br>England<br>Female<br>Funding Source<br>HIV Infections / eh [Ethnology]<br>*HIV Infections / pf [Psychosocial Factors]<br>Interviews<br>Middle Age<br>Needs Assessment<br>Patient Compliance<br>Prejudice<br>Purposive Sample<br>Structured Questionnaires<br>Truth Disclosure	Descriptors:<br>*London, England<br>*African Cultural Groups<br>*Females<br>*Acquired Immune Deficiency Syndrome<br>*Self Evaluation<br>*Stigma
**2. Godin, G Maticka-Tyndale, E, Adrien, A Singer SM, Willms, D Cappon, P, Bradet, R Daus, T, LeMay, G (1996).** *Understanding the use of condoms among Canadian*	MH - Acquired Immunodeficiency Syndrom *ethnology/*prevention & control<br>MH - Adolescent<br>MH - Adult<br>MH - Canada<br>MH - Condoms/*utilization<br>MH - Emigration and Immigration<br>MH - *Ethnic Groups<br>MH - Female<br>MH - Humans<br>MH - Male	Not found	Subject headings:<br>*Acquired Immunodeficiency Syndrome / eh [Ethnology]<br>*Acquired Immunodeficiency Syndrome / pc [Prevention and Control]<br>Adolescence<br>Adult<br>Canada<br>*Condoms / ut [Utilization]<br>Confidence Intervals<br>Emigration and Immigration	Not found

ethnocultural communities: Methods and main findings of the survey. Canadian Journal of Public Health, 87 (Suppl 1), S33-S37.	MH - Middle Aged<br>MH - Questionnaires<br>MH - Role<br>MH - Sampling Studies<br>MH - Sexual Behavior<br>MH - Social Behavior<br>MH - Time Factors		*Ethnic Groups<br>Ethnological Research<br>Female<br>Funding Source<br>Male<br>Middle Age<br>Questionnaires<br>Research Methodology<br>Risk Factors<br>*Risk Taking Behavior<br>Role<br>Sexuality<br>Summated Rating Scaling<br>Surveys	
**3.Dawson, MT Gifford, SM. (2001)** *Narratives, culture and sexual health: Personal life experiences of Salvadorean and Chilean women living in Melbourne, Australia.* Health, 5 (4), 403-423.	Not found	Subject Headings:<br>*Cross Cultural Differences<br>*Health Promotion<br>*Sexuality<br>*Sexually Transmitted Diseases<br>*Sociocultural Factors<br>HIV<br>Human Females<br>Human Migration<br>Human Sex Differences<br>Narratives<br>Prevention<br>Sex Roles<br>Strategies<br>Key concepts:<br>culture, gender, sexuality, women's health, narratives, STD prevention, prevention strategies, HIV	Not found	Descriptors:<br>*Sexual Behavior<br>*Health Education<br>*Acquired Immune Deficiency Syndrome<br>*Venereal Diseases<br>*Latin American Cultural Groups<br>*Immigrants<br>*Females<br>*Cultural sensitivity<br>*Melbourne, Australia<br>Sex roles<br>Sex role identity<br>Cultural identity

**4. Kwong-Lai Poon, M Trung-Thu Ho, P, Pui-Hing Wong, J, Wong, G & Lee, R. (2005).** *Psychosocial experiences of East and Southeast Asian men who use gay internet chatrooms in Toronto: an implication for HIV/AIDS prevention.* Ethnicity and Health , 10(2), 145-167.	MH - Asia, Southeastern/ethnology<br>MH - HIV Infections/*prevention & control<br>MH - *Homosexuality, Male<br>MH - Humans<br>MH - *Internet<br>MH - Male<br>MH - Ontario<br>MH - *Psychology	Subject Headings:<br>*AIDS Prevention<br>*Asians<br>*Internet<br>*Male Homosexuality<br>*Psychosocial Factors<br>Electronic Communication<br>Human Males<br>Southeast Asian Cultural Groups<br><br>Key concepts:<br>psychosocial experiences, East Asian men, Southeast Asian men, gay Internet chatrooms, Toronto, AIDS prevention strategies	Subject headings:<br>*Acquired Immunodeficiency Syndrome / pe [Prevention and Control]<br>*Acquired Immunodeficiency Syndrome / pf [Psychosocial Factors]<br>Adult<br>Asians<br>Attitude to Sexuality<br>Conceptual Framework<br>Descriptive Statistics<br>Grounded Theory<br>*Homosexuality<br>*Internet / ut [Utilization]<br>Interviews<br>Male<br>Ontario<br>Purposive Sample<br>Qualitative Studies<br>Snowball Sample<br>Social Isolation<br>Support, Psychosocial<br>Thematic Analysis	Descriptors:<br>*Homosexuality<br>*Internet<br>*Asian Cultural Groups<br>*Sexual Behavior<br>*Males<br>*Venereal Diseases<br>*Acquired Immune Deficiency Syndrome<br>*Prevention<br>*Psychosocial factors<br>Social isolation<br>Toronto, Ontario<br>Marginality
**5. McMunn, AM Mwanje, R Paine, K Pozniak, AL. (1998).** *Health service utilization in London's African migrant communitie: implications for HIV prevention.* AIDS Care, 10(4), 453-462.	MH - Adult<br>MH - Cross-Sectional Studies<br>MH - Family Practice/ statistics & numerical data<br>MH - Female<br>MH - HIV Infections/ethnology/ *prevention & control<br>MH - Health Promotion<br>MH - Humans<br>MH - London/epidemiology<br>MH - Male<br>MH - Middle Aged<br>MH - *Patient Acceptance of Health Care/ ethnology<br>MH - Patient Satisfaction<br>MH - Preventive Health Services/*utilization<br>MH - Sex Distribution<br>MH - Transients and Migrants<br>MH - Uganda/ethnology	Subject Headings:<br>*AIDS Prevention<br>*Health Attitudes<br>*Health Care Utilization<br>*Health Promotion<br>*HIV<br>Health Knowledge<br><br>Key concepts:<br>knowledge & attitudes & beliefs & practices of health service utilization & health promotion, Black Africans from Uganda residing in London, implications for HIV prevention	Subject headings:<br>Adult<br>Chi Square Test<br>Content Validity<br>Cross Sectional Studies<br>Descriptive Statistics<br>Female<br>HIV Education<br>*HIV Infections / pc [Prevention and Control]<br>Health Knowledge<br>*Health Resource Utilization<br>Male<br>Middle Age<br>Nonprobability Sample<br>Primary Health Care<br>Purposive Sample<br>Questionnaires<br>Semi-Structured Interview	Descriptors:<br>*Health Care Utilization<br>*London, England<br>*Southern African Cultural Groups<br>*Migrants<br>*Acquired Immune Deficiency Syndrome<br>*Prevention<br>*Health Education<br>*Client satisfaction

			Snowball Sample<br>*Transients and Migrants<br>Two-Tailed Test<br>Uganda / eh [Ethnology]<br>United Kingdom	
**6. Stolte, IG Gras, M Van Benthem, BHB Coutinho, RA Van Den Hoek, JAR. (2003).** *HIV testing behaviour among heterosexual migrants in Amsterdam.* AIDS Care, 15(4), 563-574.	MH - Adolescent<br>MH - Adult<br>MH - Cross-Sectional Studies<br>MH - Female<br>MH - HIV Infections/*diagnosis<br>MH - *Heterosexuality<br>MH - Humans<br>MH - Male<br>MH - Mass Screening/*methods<br>MH - Middle Aged<br>MH - Netherlands<br>MH - Sexual Behavior<br>MH - Sexual Partners<br>MH - *Transients and Migrants	Subject Headings:<br>*Heterosexuality<br>*HIV Testing<br>*Immigration<br>Human Sex Differences<br><br>Key concepts:<br>heterosexual migrant groups, HIV testing behavior, Amsterdam, sex differences	Not found	Not found
**7. Yoshioka, MR Schustack, A. (2001).** *Disclosure of HIV status: cultural issues of Asian patients .* AIDS Patient Care and STDs, 15(2), 77-82.	MH - Adult<br>MH - Asian Americans/*psychology<br>MH - China/ethnology<br>MH - Family<br>MH - HIV Infections/*psychology<br>MH - Homosexuality, Male/*psychology<br>MH - Humans<br>MH - Male<br>MH - Middle Aged<br>MH - *Self Disclosure<br>MH - Stress, Psychological/*etiology	Subject Headings:<br>*Asians<br>*Culture (Anthropological)<br>*Family Relations<br>*HIV<br>*Self Disclosure<br>Bisexuality<br>Family Members<br>Male Homosexuality<br>Social Values<br>Sociocultural Factors<br><br>Key concepts:<br>Asian cultural values, family members, disclosure, HIV status, Asian Americans, male homosexuality	Subject Headings:<br>*AIDS Patients / pf [Psychosocial Factors]<br>Adult<br>*Asians<br>Audiorecording<br>Cultural Values<br>Ethnological Research<br>Interviews<br>Male<br>Middle Age<br>New England<br>Qualitative Studies<br>Race Factors<br>Research Subject Recruitment<br>Thematic Analysis<br>*Truth Disclosure	Not found

**8. Chinouya, M O'Keefe, E. (2005**). *God will look after us: African, HIV and religion in Milton Keynes.* Diversity in Health and Social Care, 2,177-186.	Not found	Not found	Subject headings:<br>AIDS Serodiagnosis<br>Adult<br>Africa<br>Audiorecording<br>*Blacks<br>Christianity<br>Churches<br>Clergy<br>Communication<br>Coping<br>*Emigration and Immigration<br>England<br>Female<br>Focus Groups<br>Funding Source<br>HIV Infections / pc [Prevention and Control]<br>*HIV Seropositivity<br>Heterosexuality<br>Interviews<br>Islam<br>Male<br>Middle Age<br>Multimethod Studies<br>Prayer<br>Professional Recognition<br>Professional Role<br>*Religion and Religions<br>Self Disclosure<br>Social Isolation<br>Stigma<br>Thematic Analysis	Not found

**9. Marks, G Cantero, PJ Simoni, JM. (1998).** *Is acculturation associated with sexual risk behaviours? An investigation of HIV-positive Latino men and women.* AIDS Care, 10(3), 283-295.	MH - *Acculturation<br>MH - Adult<br>MH - Alcohol Drinking/ethnology<br>MH - Analysis of Variance<br>MH - Condoms/utilization<br>MH - Cross-Sectional Studies<br>MH - Female<br>MH - HIV Infections/*ethnology<br>MH - *Hispanic Americans<br>MH - Humans<br>MH - Los Angeles/epidemiology<br>MH - Male<br>MH - Prevalence<br>MH - Risk-Taking<br>MH - *Sexual Behavior<br>MH - Sexual Partners<br>MH - Substance-Related Disorders/ethnology	Subject Headings:<br>*Acculturation<br>*Drug Usage<br>*Hispanics<br>*HIV<br>*Sexual Risk Taking<br>Human Sex Differences<br><br>Key concepts:<br>association of acculturation & substance use prior to sex with sexual risk behaviors, HIV+ Hispanic males & females	Subject headings:<br>*Acculturation<br>Adult<br>Alcohol Drinking / eh [Ethnology]<br>Analysis of Variance<br>California<br>Chi Square Test<br>Condoms / ut [Utilization]<br>Cross Sectional Studies<br>Epidemiological Research<br>Female<br>Funding Source<br>*HIV Infections / eh [Ethnology]<br>*Hispanics<br>Male<br>Pearson's Correlation Coefficient<br>Prevalence<br>Questionnaires<br>Risk Taking Behavior<br>Sexual Partners<br>*Sexuality<br>Substance Use Disorders / eh [Ethnology]<br>United States	Descriptors:<br>*Sexual Behavior<br>*Acculturation<br>*Risk<br>*Acquired Immune Deficiency Syndrome<br>*Hispanic Americans<br>*Latin American Cultural Groups<br>*Substance Abuse<br>Los Angeles, California<br>Drug abuse
**10. Rosenthal, L Scott, DP Kelleta, Z Zikarge, A Momoh, M Lahai-Momoh, J Ross, MW Baker, A. (2003).** *Assessing the HIV/AIDS health services needs of African immigrants to Houston.* AIDS Education and Prevention, 15(6), 570-580.	MH - Africa/ethnology<br>MH - Condoms/utilization<br>MH - *Emigration and Immigration<br>MH - Female<br>MH - HIV Infections/*therapy<br>MH - Health Knowledge, Attitudes, Practice<br>MH - Health Services Needs and Demand/ *statistics & numerical data<br>MH - Humans<br>MH - Male<br>MH - Sexual Behavior<br>MH - Texas	Subject Headings:<br>*Health Care Utilization<br>*Health Knowledge<br>*Health Service Needs<br>*HIV<br>*Immigration<br>AIDS<br>Health Attitudes<br><br>Key concepts:<br>HIV, AIDS, health service needs, African immigrants, HIV/AIDS knowledge, risk behaviors, health service access	not found	Descriptors:<br>*Acquired Immune Deficiency Syndrome<br>*Risk<br>*Immigrants<br>*Southern African Cultural Groups<br>*Houston, Texas<br>*Stigma<br>*Health Care Services

**11. Castro-Vazquez, G Tarui, M (2006).** *'Pueblo chico, infierno grande': community support and HIV/AIDS among HIV-positive Latin Americans living in Japan.* Ethnicities, 6(1), 52-73.	Not found	Not found	Not found	Not found
**12. Fenton, KA Chinouya, M Davidson, O Copas, (2002).** *HIV testing and high risk sexual behaviour among London's migrant African communities: a participatory research study.* Sexually Transmitted Infections, 78, 241-245.	MH - Adolescent<br>MH - Adult<br>MH - Africa South of the Sahara/ethnology<br>MH - Age Factors<br>MH - Aged<br>MH - Chi-Square Distribution<br>MH - Condoms/utilization<br>MH - Cross-Sectional Studies<br>MH - Female<br>MH - HIV Infections/*diagnosis/ethnology<br>MH - Humans<br>MH - Logistic Models<br>MH - London/epidemiology<br>MH - Male<br>MH - Middle Aged<br>MH - Prevalence<br>MH - Regression Analysis<br>MH - *Risk-Taking<br>MH - Sex Factors<br>MH - Sexual Behavior/ *statistics & numerical data<br>MH - Sexual Partners	Not found	Not found	Not found

**13. Deren, S Kang, SY Colon, HM Andia, JF. (2003).** *Migration and HIV risk behaviours: Puerto Rican injectors in New York City and Puerto Rico.* American Journal of Public Health, 93 (5), pp812-816.	MH - Adolescent<br>MH - Adult<br>MH - Emigration and Immigration/ *statistics & numerical data<br>MH - Female<br>MH - HIV Infections/*ethnology/etiology<br>MH - Homeless Persons<br>MH - Humans<br>MH - Male<br>MH - Needle Sharing/adverse effects<br>MH - New York City/epidemiology<br>MH - Odds Ratio<br>MH - Puerto Rico/epidemiology/ethnology<br>MH - *Risk-Taking<br>MH - Street Drugs/classification<br>MH - Substance Abuse, Intravenous/ complications/*ethnology	Subject Headings:<br>*Drug Administration Methods<br>*HIV<br>*Human Migration<br>*Injections<br>*Risk Taking<br>At Risk Populations<br>Intervention<br>Urban Environments<br><br>Key concepts:<br>New York City, Puerto Rico, HIV risk behaviors, intervention services, Puerto Rican Drug Injectors, migration status, homelessness, risk level, injection drug users	Subject headings:<br>Adult<br>Comparative Studies<br>Confidence Intervals<br>Descriptive Statistics<br>Female<br>Focus Groups<br>Funding Source<br>HIV Education<br>*HIV Infections / pc [Prevention and Control]<br>*HIV Infections / rf [Risk Factors]<br>Health Services Accessibility<br>Homelessness<br>Interviews<br>*Intravenous Drug Users<br>*Intravenous Drug Users<br>Male<br>Middle Age<br>Multiple Logistic Regression<br>Multiple Regression<br>Needle Sharing<br>New York<br>Odds Ratio<br>P-Value<br>Puerto Rico<br>Research Subject Recruitment<br>*Risk Taking Behavior<br>*Substance Abuse, Intravenous<br>Substance Use Rehabilitation Programs / ut [Utilization]<br>Time Factors<br>*Transients and Migrants<br>Urinalysis	Not found

**14. Wiggers, L deWit, JBF Gras, MJ Coutinho, RA Van den Hoek, A.(2003).** *Risk behaviour and social –cognitive determinants of condom use among ethnic minority communities in Amsterdam.* AIDS Education and Prevention, 15 (5), 430-447.	MH - Adolescent<br>MH - Adult<br>MH - Africa/ethnology<br>MH - Chi-Square Distribution<br>MH - Condoms/*utilization<br>MH - Cross-Sectional Studies<br>MH - *Emigration and Immigration<br>MH - *Ethnic Groups<br>MH - Female<br>MH - HIV Seropositivity/diagnosis/ epidemiology<br>MH - Humans<br>MH - Logistic Models<br>MH - Male<br>MH - Netherlands/epidemiology<br>MH - Netherlands Antilles/ethnology<br>MH - Prevalence<br>MH - *Risk-Taking<br>MH - *Sexual Behavior<br>MH - Suriname/ethnology	Subject Headings:<br>*Condoms<br>*Minority Groups<br>*Sexual Attitudes<br>*Sexual Risk Taking<br>*Social Cognition<br>Psychosocial Factors<br>Racial and Ethnic Differences<br><br>Key concepts:<br>sexual risk behaviors, condom use, ethnic minorities social cognitive factors, Amsterdam	Not found	Not found
**15. Shedlin, MG Decena, CU Oliver-Velez, D. (2005).** *Initial acculturation and HIV risk among new Hispanic immigrants.* Journal of the National Medical Association, 97 (7), s32-s35.	MH - *Acculturation<br>MH - Attitude to Health/*ethnology<br>MH - Central America/ethnology<br>MH - *Emigration and Immigration<br>MH - Female<br>MH - Focus Groups<br>MH - HIV Infections/epidemiology/ *ethnology<br>MH - Health Behavior/*ethnology<br>MH - Hispanic Americans/ethnology/ *psychology<br>MH - Humans<br>MH - Interviews<br>MH - Male<br>MH - New York City/epidemiology<br>MH - Qualitative Research<br>MH - Risk Assessment<br>MH - Risk Factors<br>MH - Time Factors	Not found	Not found	Not found

**16. Lazarus, JV Himedan, HM Ostergaard, LR Liljestrand, J. (2006).** *HIV/AIDS knowledge and condom use among Somali and Sudanese immigrants in Denmark.* Scandanavian Journal of Public Health, 34, 92-99.	MH - Acquired Immunodeficiency Syndrome/epidemiology/prevention & control/*transmission<br>MH - Adolescent<br>MH - Adult<br>MH - *Condoms<br>MH - Denmark/epidemiology/ethnology<br>MH - *Emigration and Immigration<br>MH - Female<br>MH - HIV Infections/epidemiology/prevention & control/*transmission<br>MH - Health Education<br>MH - *Health Knowledge, Attitudes, Practice<br>MH - Humans<br>MH - Incidence<br>MH - Interviews<br>MH - Male<br>MH - Questionnaires<br>MH - Sexual Behavior<br>MH - Somalia/ethnology<br>MH - Sudan/ethnology	Not found	Subject headings:<br>Adult<br>Age Factors<br>*Attitude to AIDS<br>Birth Place<br>Chi Square Test<br>*Condoms / ut [Utilization]<br>Data Analysis Software<br>Denmark<br>Descriptive Statistics<br>Educational Status<br>Emigration and Immigration<br>Female<br>Fisher's Exact Test<br>Funding Source<br>*HIV Education<br>*HIV Infections / tm [Transmission]<br>Health Beliefs<br>*Health Knowledge / ev [Evaluation]<br>*Immigrants / pf [Psychosocial Factors]<br>Male<br>Middle Age<br>Purposive Sample<br>Questionnaires<br>Semi-Structured Interview<br>Sex Factors<br>Somalia<br>Sudan<br>Thematic Analysis	Not found

**17. Kang, E, Rapkin, BD, Springer, C Kim, JH. (2003).** *The 'Demon Plague' and access to care among Asian undocumented immigrants living with HIV diseases in New York City.* Journal of Immigrant Health, 5(2), 49-58.	MH - Adolescent<br>MH - Adult<br>MH - Asian Americans/*psychology<br>MH - Attitude to Health/*ethnology<br>MH - Child<br>MH - Child, Preschool<br>MH - *Emigration and Immigration<br>MH - Female<br>MH - HIV Infections/epidemiology/ *ethnology/therapy<br>MH - *Health Services Accessibility<br>MH - Health Services Needs and Demand<br>MH - Health Services Research<br>MH - Humans<br>MH - Male<br>MH - New York City/epidemiology<br>MH - Pacific Islands/ethnology<br>MH - Social Work	Not found	Subject headings:<br>Acquired Immunodeficiency Syndrome / pf [Psychosocial Factors]<br>Adult<br>*Asians<br>Attitude<br>Female<br>Focus Groups<br>Funding Source<br>*HIV Seropositivity / th [Therapy]<br>Health Behavior<br>*Health Services Accessibility<br>*Immigrants<br>Male<br>Medical Care / ut [Utilization]<br>New York<br>Perception<br>Qualitative Studies<br>Semi-Structured Interview<br>Thematic Analysis	Not found
**18. Poppen, PJ, Reisen, CA, Zea, MC Bianchi, FT Echeverry, JJ (2005).** *Serostatus disclosure, seroconcordance, partner relationship, and unprotected anal intercourse among HIV-positive Latino men who have sex with men.* AIDS Education and Prevention. 17(3), 227-237.	MH - Adult<br>MH - *Anal Canal<br>MH - *HIV Seropositivity<br>MH - Hispanic Americans/*psychology<br>MH - *Homosexuality, Male<br>MH - Humans<br>MH - Male<br>MH - Middle Aged<br>MH - *Self Disclosure<br>MH - United States<br>MH - *Unsafe Sex	Subject Headings:<br>*HIV<br>*Male Homosexuality<br>*Safe Sex<br>*Sexual Risk Taking<br>*Risk Assessment<br>Hispanics<br>Self Disclosure<br>Sexual Partners<br><br>Key concepts:<br>serostatus disclosure, seroconcordance, sexual partner relationship, unprotected anal intercourse, HIV positive Latino men, homosexual men	Not found	Descriptors:<br>*Human immunodeficiency virus--HIV<br>*Health risk assessment<br>*Sexual behavior<br>*Hispanic Americans<br>*Epidemics<br>*Gays & lesbians

**19. Korner, H (2007).** *'If I had my residency I wouldn't worry': Negotiating migration and HIV in Sydney, Australia.* Ethnicity and Health. 12(3), 205-225.	MH - Acculturation<br>MH - Adolescent<br>MH - Adult<br>MH - Emigration and Immigration/ *legislation & jurisprudence/trends<br>MH - Female<br>MH - HIV Seropositivity/ethnology/ *psychology<br>MH - *Health Policy<br>MH - *Health Services Accessibility<br>MH - Humans<br>MH - Interpersonal Relations<br>MH - Interviews<br>MH - Language<br>MH - Life Change Events<br>MH - Male<br>MH - Middle Aged<br>MH - Narration<br>MH - New South Wales/epidemiology<br>MH - Quality of Life/*psychology<br>MH - Social Support<br>MH - Uncertainty<br>MH - Urban Health Services/*utilization	Not found	Subject headings:<br>Adult<br>Attitude to AIDS<br>Audiorecording<br>Culture<br>Descriptive Statistics<br>*Emigration and Immigration<br>Female<br>Funding Source<br>*HIV Infections / pf [Psychosocial Factors]<br>Health Services Accessibility<br>*Hepatitis C / pf [Psychosocial Factors]<br>Male<br>Middle Age<br>New South Wales<br>Phenomenological Research<br>Semi-Structured Interview<br>Social Networks<br>Stigma<br>Support, Psychosocial<br>*Transients and Migrants / pf [Psychosocial Factors]	Descriptors:<br>*Migrants<br>*Immigration Policy<br>*Relocation<br>*Acquired Immune Deficiency Syndrome<br>*Australia<br>*Stigma

**20. Nemoto, T Iwanamoto, M Oh, HJ Wong, S Nguyen, H. (2005).** *Risk behaviours among Asian women who work at massage parlours in San Francisco: Perspectives from masseuses and owner/managers.* AIDS Education and Prevention 17(5), 444-456	MH - Asian Americans/*psychology<br>MH - Condoms/utilization<br>MH - Female<br>MH - Focus Groups<br>MH - HIV Infections/ethnology/transmission<br>MH - Humans<br>MH - Interviews<br>MH - Massage/*manpower<br>MH - Prostitution/*ethnology<br>MH - *Risk-Taking<br>MH - San Francisco/epidemiology<br>MH - Sexually Transmitted Diseases/ epidemiology/ethnology<br>MH - Substance-Related Disorders/epidemiology/ ethnology<br>MH - Thailand/ethnology<br>MH - Unsafe Sex/*ethnology/ statistics & numerical data<br>MH - Vietnam/ethnology	Subject Headings:<br>*AIDS Prevention<br>*Asians<br>*Human Females<br>*Sexual Risk Taking<br>*Sociocultural Factors<br>Business<br>HIV<br>Massage<br>Risk Factors<br>Sex<br><br>Key concepts:<br>risk behavior, Asian women, massage parlor workers, cognitive factors, cultural factors, contextual factors, sex work, masseuses, parlor owners & managers	Subject headings:<br>*Asians<br>*Asians / pf [Psychosocial Factors]<br>Audiorecording<br>Condoms / ut [Utilization]<br>Descriptive Statistics<br>Female<br>Focus Groups<br>Funding Source<br>HIV Infections / eh [Ethnology]<br>HIV Infections / ep [Epidemiology]<br>Interviews<br>*Massage<br>*Prostitution / eh [Ethnology]<br>*Risk Taking Behavior / eh [Ethnology]<br>Sexually Transmitted Diseases / eh [Ethnology]<br>Sexually Transmitted Diseases / ep [Epidemiology]<br>Socioeconomic Factors<br>Substance Use Disorders / eh [Ethnology]<br>Substance Use Disorders / ep [Epidemiology]<br>Thailand<br>United States	Descriptors:<br>*Prostitution<br>*Southeast Asian Cultural Groups<br>*Asian Americans<br>*Acquired Immune Deficiency Syndrome<br>*Sexual Behavior<br>*Risk Factors<br>*Females<br>San Francisco, California
**TOTAL** (found on each database)	**17**	**12**	**12**	**9**
