# Supplementary material for: HIV among immigrants living in high-income countries: a realist review of evidence to guide targeted approaches to behavioural HIV prevention
Source: Syst Rev. 2012 Nov 20;1:56. doi: 10.1186/2046-4053-1-56 (PMC3534573; doi:10.1186/2046-4053-1-56)
Supplement: Additional file 3 — Results: generating candidate mechanisms. [file 2046-4053-1-56-S3.pdf]

Results: generating candidate mechanisms

The initial explanatory framework of behavioural HIV prevention explored themes in the literature in relation to 'how' and 'why' interventions with immigrants were supposed to work and their limitations [1-3]. A dominant theme was one where 'cultural appropriateness' was assumed as a key principle for interventions in this area but where this principle was rarely defined or discussed in detail [4-9]. Even though 'culture' is a broader term, the term as used in this literature was narrower and more or less synonymous with ethnicity [6]. Another dominant theme was a strong critique of the cognitive and behavioural individualistic approaches to HIV prevention, mainly for their failure to account for the social and cultural influences, as well as the structural and environmental constraints which might impede behaviour change among immigrants [9-17]. In response to this pervasive critique there are a range of techniques of adapting interventions to 'suit' the 'culture' of the target group in the literature [8, 18-23].

Two key adaptation activities frequently cited in the literature were staffing the intervention with bicultural staff who were 'matched' to the target population, [6, 7, 9, 17-19, 22, 24-27] along with responding to the linguistic needs of the target population [5, 9, 16, 18, 22, 26-28]. A third adaptation activity related to adapting the intervention content to achieve congruence with the target audience in terms of values, norms, symbols or metaphors [6, 9, 14-20, 24, 26, 27]. Planning and implementing the intervention to respond specifically to the ethnicity of the target population was a fourth adaptation activity in the literature (e.g., targeting Mexican rather than Hispanic immigrants) [7, 10, 20, 24, 28, 29]. Delivering the intervention in settings and through structures familiar to immigrant populations such as places of worship, beauty salons, or through immigrant mass media was a fifth adaptation activity [5, 15-17, 20]. Securing the endorsement of the target population through consultation processes was cited as another potential adaptation activity to achieve 'cultural appropriateness' in interventions with immigrants [9, 13, 20, 27, 28, 30]. Another less cited adaptation activity centred on the degree to which the goals of the intervention 'matched' immigrant community expectations around the effectiveness of the intervention [7, 16, 23, 26, 31]. These broad adaptation activities were theorised to be across the dimensions of 'staffing', 'language', 'content', 'ethnic diversity', 'settings', 'community consultation' and 'priority setting' and led to the development of the initial program theories summarised below.

Results: adaptive mechanisms

These intervention adaptation activities – 'staffing', 'language', 'content', 'ethnic diversity', 'settings', 'community consultation' and 'priority setting' – were then further tested using methods [32, 33] against the literature from the 'known studies' and other studies to populate seven intervention 'chains', each made up of activities, mechanisms, response and resistances.

The 'staffing' and 'language' adaptation activities were widely reported in the literature on HIV prevention among immigrants in high-income countries [10, 16, 28, 34-49]. The responses of immigrant participants to the 'matching' of 'staffing' was that the intervention was 'for them' – as the staff delivering the intervention were 'like them' [10, 16, 28, 34-49]. The mechanism involved in 'staffing' was thus theorised to be around the '*authenticity*' of the intervention. The responses of immigrants to the 'matching' of 'language' was that, as the intervention was in their first language, it was possible for them to understand the intervention [10, 16, 28, 34-49] and the mechanism involved in 'language' was theorised as being around *'understanding'* the intervention. Using an example of an intervention with Latino immigrants in the USA, '*authenticity*' and '*understanding*' mechanisms were hypothesised to operate when, for example, Latino facilitators delivered a series of HIV education sessions to groups of Latino immigrants in Spanish.

In terms of the 'content' and 'ethnic diversity' adaptation activities, intervention participants responded positively to the 'matching' of the intervention 'content' as it was congruent with elements of their own culture [10, 16, 34, 35, 37, 38, 43, 44, 46, 47, 49, 50]. Thus the theorised mechanism involved in 'content' activities was around the '*consonance*' of the intervention. When interventions were targeted in terms of 'ethnic diversity' participants indicated that the intervention was specific to their ethnicity [10, 37, 40, 43, 47, 49]. The theorised mechanism involved in 'ethnic diversity' activities hinged on the '*specificity*' of the intervention. Sticking with the example of an intervention with Latino immigrants '*consonance*' was hypothesised to be operating when Latino cultural norms such as *machismo* were drawn on for the intervention content. '*Specificity*' was hypothesised to be operating when Mexicans, rather than Latinos, were specifically targeted by the intervention.

The 'settings', 'community consultation' and 'priority setting' adaptation activities were cited less frequently in the literature than other adaptation activities [10, 16, 34, 35, 37, 38, 40, 42, 43, 49, 51-

53]. Intervention participants were reported to respond positively to engaging with interventions in familiar 'settings' like cafes or mosques or through immigrant media [10, 16, 34, 37, 38, 40, 42, 43]. This pointed to a theorised mechanism involved in 'settings' activities that drew on the '*embeddedness*' of the intervention. 'Community consultation' activities were reported to result in greater support from immigrants for intervention strategies [16, 35, 37, 49, 51-53] which indicated that the underlying mechanism involved was one of '*endorsement*'. Finally, the 'priority setting' adaptation activity was reported to result in immigrant participants' expectations being more strongly aligned with the intervention goals and intended outcomes [10, 43] and could enhance the 'match' between intervention outcomes and immigrant expectations of effectiveness [31]. The mechanism theorised to be involved in 'priority setting' was around the '*framing*' of the intervention. Keeping with our example of an intervention with Latinos in the USA, '*embeddedness*' is hypothesised to operate in an intervention being delivered at farms where Mexican migrant day labourers work. The '*endorsement*' mechanism operates when an intervention consults extensively with Mexican immigrants to garner their support for intervention strategies. Finally, the '*framing*' mechanism is operating when an intervention for Mexican immigrants engages with a Mexican community organisation in 'priority setting' around intervention outcomes. These seven mechanisms were theorised as the key, rather than the only, interrelated mechanisms contributing to cultural appropriateness in interventions with immigrants. These mechanisms typically needed to be inferred from the literature as most reports and studies only referred to health promotion theories such as the Health Belief Model, Stages of Change Model or Social Cognitive Theory as the key theories underpinning interventions with immigrants.

The adaptation activities can also be resisted by participants pointing to why things 'went wrong'. At a staffing level intervention participants might resist 'matching' [54] staff along the lines of ethnicity and have a better response to 'experts' [25]. The strength of targeting using ethnicity may lead to stereotyping of immigrant communities and face resistance. Cultural community norms which are integrated into the content of an intervention may promote or impede HIV prevention efforts [55] or an intervention may be seen as too progressive by promoting the rights of women or gays [13, 55]. An extension of this kind of resistance in immigrant community settings and institutions could explain the sometimes hostile responses to HIV agencies which attempt to build alliances or gain endorsement for interventions [51]. For example, immigrant religious institutions may be particularly unwilling to engage given what they may perceive as a contradiction between their role as sources of compassion and guardians of morality [53].

References:

1. Pawson, R., et al., *Realist review-a new method of systematic review designed for complex policy interventions.* Journal of Health Services Research and Policy, 2005. **10**(Suppl 1): p. 21-34.
2. Pawson, R., et al., *Realist synthesis: an introduction*, in *Research Methods Paper 2*. 2004, ESRC, University of Manchester.
3. Pawson, R., *Evidence based policy: A realist perspective*. 2006, London: Sage.
4. Hoffman-Goetz, L. and D.B. Friedman, *A systematic review of culturally sensitive cancer prevention resources for ethnic minorities.* Ethnicity and Diseases, 2006. **16**: p. 971-977.
5. UNAIDS and International Organisation for Migration (IOM), *Migration and AIDS.* International Migration, 1998. **36**(4): p. 445-468.
6. Wilson, B.D.M. and R.L. Miller, *Examining strategies for culturally grounded HIV prevention: A review.* Aids Education and Prevention, 2003. **15**(2): p. 184-202.
7. Raj, A., H. Amaro, and E. Reed, *Culturally tailoring HIV/AIDS prevention programs: Why, When, and How*, in *Handbook of Cultural Health Psychology*, S. Kazarian and D. Evans, Editors. 2001. p. 195-239.
8. Vinh-Thomas, P., M.M. Bunch, and J.J. Card, *A research-based tool for identifying and strengthening culturally competent and evaluation-ready HIV/AIDS prevention programs.* Aids Education and Prevention, 2003. **15**(6): p. 481-498.
9. Kreuter, M.W., et al., *Achieving cultural appropriateness in health promotion programs: targeted and tailored approaches.* Health Education & Behavior, 2003. **30**(2): p. 133-146.
10. Organista, K.C., H. Carrillo, and G. Ayala, *HIV prevention with Mexican immigrants: Review, critique, and recommendations.* Journal of Acquired Immune Deficiency Syndrome, 2004. **37**(Suppl 4): p. s227-S239.
11. Chng, L.C., et al., *A model for understanding sexual health among Asian American/Pacific Islander Men who have Sex with Men (MSM) in the United States.* Aids Education and Prevention, 2003. **15**(Suppl A): p. 21-38.
12. Nemoto, T., et al., *HIV seroprevalence, risk behaviours, and cognitive factors among Asian and Pacific Islander Men who have Sex with Men (MSM): a summary and critque of empirical studies and methodological issues.* Aids Education and Prevention, 1998. **10**(Suppl A): p. 31-47.
13. Ramirez-Valles, J., *The quest for effective HIV prevention interventions for Latino gay men.* American Journal of Preventive Medicine, 2007. **32**(4S): p. S34-S35.
14. Herbst, J.H., et al., *A systematic review and meta-analysis of behavioral interventions to reduce HIV risk behaviors of Hispanics in the United States and Puerto Rico.* AIDS Behavior, 2007. **11**(1): p. 25-47.
15. Brooks, R.A., et al., *Preventing HIV among Latino and African American gay and bisexual men in a context of HIV-related stigma, discrimination, and homophobia: Perspectives of providers.* Aids Patient Care and Stds, 2005. **19**(11): p. 737-744.
16. Conner, R.F., S.I. Mishra, and R.J. Magana, *HIV prevention policies and programs: Perspectives from researchers, migrant workers and policymakers*, in *AIDS crossing borders: The spread of HIV among migrant Latinos*, S.I. Mishra, R.F. Conner, and R.J. Magana, Editors. 1996, Westfield Press: Boulder, Colorado. p. 186-214.
17. Peterson, J.L. and A. Carballo-Dieguez, *HIV prevention among African-American and Latino men who have sex with men*, in *Handbook of HIV Prevention*, J.L. Peterson and R. DiClemente, Editors. 2000, Kluwer Academic/Plenum Publishers: New York. p. 217-224.
18. Brach, C. and I. Fraser, *Can cultural competency reduce racial and ethnic health disparities? A review and conceptual model.* Medical Care Research and Review, 2000. **57**: p. 181-217.

19. Scott, K.D., A. Gilliam, and K. Branxton, *Culturally competent HIV prevention strategies from women of color in the United States.* Health Care for Women International, 2005. **26**: p. 17-45.
20. Grzywacz, J.G., et al., *Serving racial and ethnic populations with health promotion.* American Journal of Health Promotion, 2004. **18**(5): p. 8-12.
21. Chng, L.C. and J.R. Collins, *Providing culturally competent HIV prevention programs.* American Journal of Health Studies, 2000. **16**(1): p. 24-33.
22. Betancourt, J.R., et al., *Defining cultural competence: A practical framework for addressing racial/ethnic disparities in health and health care.* Public Health Reports, 2003. **118**: p. 293-301.
23. SenGupta, S., R. Hopson, and M. Thompson-Robinson, *Cultural competence in evaluation: An overview.* New Directions for Evaluation, 2004(102): p. 5-19.
24. Darbes, L., et al. *Systematic review of HIV behavioural prevention research in Asian Americans and Pacific Islanders*. 2002 [cited 2008 3 May]; Available from: http://hivinsite.ucsf.edu.
25. Durantini, M.R., et al., *Conceptualizing the influence of social agents of behaviour change: a meta-analysis of the effectiveness of HIV-prevention interventionists for different groups.* Psychological Bulletin, 2006. **132**(2): p. 212-248.
26. Marin, B.V., *HIV prevention in the Hispanic community: Sex, culture and empowerment.* Journal of Transcultural Nursing, 2003. **14**(3): p. 186-192.
27. Villarruel, A.M., L.S. Jemmott, and J.B. Jemmott, *Desiging a culturally based intervention to reduce HIV sexual risk for Latino adolescents.* Journal of the Association of Nurses in AIDS Care, 2005. **16**(2): p. 23-31.
28. Darbes, L., G. Kennedy, and G. Rutherford. *Systematic review of interventions to prevent HIV infection in MSM of color*. California Collaborations in HIV Prevention Research 2004 [cited 2007 11 November]; Available from: http://chrp.ucop.edu/ca_collaborations/modules/research_summary.html.
29. Darbes, L., G. Kennedy, and G. Rutherford. *Systematic review of behavioural prevention interventions to prevent HIV infection in communities of color*. 2005 [cited 2008 3 May]; Available from: http://hivinsite.ucsf.edu/.
30. Freudenberg, N. and U. Trinidad, *The role of community organisations in AIDS prevention in Latino communities in New York City.* Health Education Quarterly, 1992. **19**(2): p. 219-232.
31. Madison, A., *New Directions for Evaluation: Coverage of cultural issues and issues of significance to under-represented groups.* New Directions for Evaluation, 2007. **114**: p. 107-114.
32. Weiss, C.H., *Theory-based evaluation: Past, present and future.* New Directions for Evaluation, 1997. **76**: p. 68-81.
33. Weiss, C.H., *Which links in which theories shall we evaluate?* New Directions for Evaluation, 2000. **87**: p. 35-45.
34. Amaro, H., et al., *Implementation and long-term outcomes of two HIV intervention programs for Latinas.* Health Promotion Practice, 2002. **3**(2): p. 245-54.
35. Carballo-Dieguez, A., et al., *A randomized controlled trial to test an HIV-prevention intervention for Latino gay and bisexual men: Lessons learned.* AIDS Care, 2005. **17**(3): p. 314-328.
36. Dushay, R.A., et al., *Lowering HIV risk among ethnic minority drug users: Comparing culturally targeted intervention to a standard intervention.* American Journal of Drug & Alcohol Abuse, 2001. **27**(3): p. 501-524.
37. Haour-Knipe, M., F. Fleury, and F. Dubois-Arber, *HIV/AIDS prevention for migrants and ethnic minorities: three phases of evaluation.* Social Science & Medicine, 1999. **49**(10): p. 1357-1372.

38. Hovey, J.D., V. Booker, and L.D. Seligman, *Using theatrical presentations as a means of disseminating knowledge of HIV/AIDS risk factors to migrant farmworkers: an evaluation of the effectiveness of the Informate program.* Journal of Immigrant and Minority Health, 2007. **9**(2): p. 147-56.
39. Kaplan, E., et al., *A model-based evaluation of a cultural mediator outreach program for HIV-positive Ethiopian immigrants in Israel.* Evaluation Review, 2002. **26**(4): p. 382-394.
40. Kocken, P., et al., *Effects of peer-led AIDS education aimed at Turkish and Moroccan male immigrants in the Netherlands: A randomised controlled evaluation study.* European Journal of Public Health 2001. **11**(2): p. 153-159.
41. Martijn, C., et al., *The effects of AIDS prevention programs by lay health advisors for migrants in The Netherlands.* Patient Education and Counseling, 2004. **53**(2): p. 157-65.
42. McMahon, T., et al., *Evaluation of an ethnic media campaign on patterns of HIV testing among people from culturally and linguistically diverse backgrounds in Australia.* Sexual Health, 2004. **1**(2): p. 91-94.
43. McQuiston, C. and J.H. Flaskerud, *"If they don't ask about condoms, I just tell them": A descriptive case study of Latino lay health advisers' helping activities.* Health Education & Behavior, 2003. **30**(1): p. 79-96.
44. Nyamathi, A.M., et al., *Evaluation of two AIDS education programs for impoverished Latina women.* Aids Education and Prevention, 1994. **6**(4): p. 296-309.
45. Operario, D., et al., *Conducting HIV interventions for Asian and Pacific Islander men who have sex with men: challenges and compromises in community collaborative research.* Aids Education and Prevention, 2005. **17**(4): p. 334-336.
46. Peragallo, N., et al., *A randomized clinical trial of an HIV-risk-reduction intervention among low-income Latina women.* Nursing Research, 2005. **54**(2): p. 108-118.
47. Soskolne, V. and R. Shtarkshall, *Migration and HIV prevention programmes: linking structural factors, culture, and individual behaviour - an Israeli experience.* Social Science and Medicine, 2002. **55**: p. 1297-1307.
48. Wong, F.Y., L.C. Chng, and W. Lo, *A profile of six community-based HIV prevention programs targeting Asian and Pacific Islander Americans.* Aids Education and Prevention, 1998. **10**(Suppl A): p. 61-76.
49. Worth, H., N. Denholm, and J. Bannister, *HIV/AIDS and the African Refugee Education Program in New Zealand.* AIDS Education and Prevention, 2003. **15**(4): p. 346-356.
50. Choi, K.-H., et al., *The efficacy of brief group counseling in HIV risk reduction among homosexual Asian and Pacific Islander men.* AIDS, 1996. **10**(1): p. 81-87.
51. Chin, J.J., et al. (2008) *Paradigm shifters, professionals, and community sentinels: Immigrant community institutions' roles in shaping places and implications for stigmatized public health initiatives*. Health & Place **Volume**, 866-882
52. Loue, S., L. Lloyd, and E. Phoombour, *Organising Asian and Pacific Islanders in an urban community to reduced HIV risk: A case study.* AIDS Education and Prevention, 1996. **8**(5): p. 381-393.
53. Chin, J.J., et al., *Chinese and South Asian religious institutions and HIV prevention in New York City.* Aids Education and Prevention, 2005. **17**(5): p. 484-502.
54. Idali Torres, M. and G.P. Cernada, *Cultural landscapes and cultural brokers of sexual and reproductive health in US Latino and Latin American populations.* International Quarterly of Community Health Education, 2001/2002. **21**(2): p. 109-132.
55. Ortiz-Torres, B., I. Serrano-Garcia, and N. Torres-Burgos, *Subverting culture: Promoting HIV/AIDS among Puerto RIcan and Dominican women.* American Journal of Community Psychology, 2000. **28**(6): p. 859-879.
