# Supplementary material for: HIV among immigrants living in high-income countries: a realist review of evidence to guide targeted approaches to behavioural HIV prevention
Source: Syst Rev. 2012 Nov 20;1:56. doi: 10.1186/2046-4053-1-56 (PMC3534573; doi:10.1186/2046-4053-1-56)
Supplement: Additional file 4 — Collation of intervention studies included in the review. [file 2046-4053-1-56-S4.pdf]

Collation of intervention studies included in the review

First Author	Location	Intervention	Intervention participants	Intervention theory or model	Adaptation of intervention activities to increase cultural appropriateness	Key Results	Key Limitations	Study Design	Notes
Amaro (2002)	Boston, USA	Comparison of two interventions for immigrant women:<br>1. HIV-specific with participatory education;<br>2. women's health specific and didactic<br><br>Each intervention 12 group sessions & 16 hours of content with focus on sexual risk reduction	170 Latina women<br><br>Dominican 55%, Puerto Rican 13%<br><br>% of immigrants not stated but inferred to be > 65% due to participant recruitment methods	1- Social Cognitive Theory (SCT)<br><br>Empowerment Theory<br><br>2- SCT<br><br>Theory of Reasoned Action (TRA)<br><br>Health Belief Model (HBM)	Language (Spanish)<br><br>Bicultural Community Educators (BCEs) – to facilitate groups<br><br>Gender - of group facilitator<br><br>Content-use cultural elements in curriculum	Diminishing effects of intervention at 15- months when compared effects measured by Raj et al at 3-months (2001)<br><br>High satisfaction of program participants	Study Design<br><br>Difficulty of recruiting and retaining participants in a multi-session program<br><br>Did not focus on male partners of participants	Quasi-experimental	15 month follow-up of Raj et al (2001)
Carballo-Dieguez (2005)	New York City, USA	8 group sessions with a focus on sexual risk reduction for immigrant gay men	180 Latino gay and bisexual men<br><br>78% immigrants in experimental group (similar to control group)<br><br>HIV-positive and HIV-negative men	Empowerment Theory	Language (Spanish)<br><br>Bicultural Community Educators (BCEs) – gay BCEs developed content developed<br><br>Gender and sexuality - of group facilitator<br><br>Content – use of 'dichos' Spanish proverbs	No statistical difference in reduction in risk behaviours (two main outcome measures) between control and experimental group at 2, 6 and 12-months	Experimental group may have been prone to selection bias<br><br>Baseline assessment may have had an effect on experimental & control groups<br><br>Empowerment focus may have obscured resilience	RCT	

First Author	Location	Intervention	Intervention participants	Intervention theory or model	Adaptation of intervention activities to increase cultural appropriateness	Key Results	Key Limitations	Study Design	Notes
Chemtov (1993)	Israel	200 group sessions to raise awareness of HIV/AIDS in a newly-arrived immigrant community<br><br>A range of supporting activities (e.g. health promotion resources) to reach 24,000 newly arrived immigrants	24, 000 Ethiopian – born immigrants, men and women<br><br>100% immigrants (inferred)	Not stated	Language (Amharic)<br><br>Bicultural Community Educators (BCEs) – trained 34 "cultural mediators" to deliver a program in various ways –mainly education sessions<br><br>Content – use of popular proverbs<br><br>Settings- sessions were delivered in Ethiopian community settings (inferred)	Estimated (from post-intervention survey) that 60% of Ethiopian immigrants had been reached by intervention	Unknown if program has had an effect on behaviour<br><br>Intervention may have had limited appeal to HIV-positive people	Random post-intervention survey	Related to Shtarkshall (2000) & Soskolne (2002)
Choi (1996)	San Francisco, USA	Single group session with a focus on sexual risk reduction for immigrant and ethnic minority gay and bisexual men	329 Asian and Pacific Islander gay men<br><br>37% Chinese 34% Filipino<br><br>67% immigrants<br><br>HIV-positive and HIV-negative men	Empowerment Theory (inferred)	Content – exploration of positive ethnic and sexual identities<br><br>Gender and sexuality - of co-facilitator of group	Significantly reduced no. of sexual partners in intervention group<br><br>Significantly increased knowledge at 3 months in intervention group<br><br>No significant reduction in other outcome measures.	No effect in perceived sexual risk or sexual negotiation<br><br>Unknown longer-term effects<br><br>Effect strongest in Chinese & Filipino participants<br><br>Unable to address wider social contexts	RCT	

First Author	Location	Intervention	Intervention participants	Intervention theory or model	Adaptation of intervention activities to increase cultural appropriateness	Key Results	Key Limitations	Study Design	Notes
Conner (2004)	California, USA	Evaluation of two interventions<br><br>1. Tres Hombres sin Fronteras – a health promotion resource with a focus on sexual risk reduction for immigrant farmworkers<br><br>2. SOLAAR<br>- retreat for small groups with a focus on sexual risk reduction for immigrant gay men	1. Latino farm workers(mainly Mexican)<br><br>% immigrants – not stated but inferred to be >65%<br><br>2. Latino gay and bisexual men(mainly Mexican)<br><br>% immigrants – not stated but inferred to be >65%	1. Not stated<br><br>2. Stages of Change (SC) (inferred)<br><br>Social Marketing	1. Language (Spanish)<br><br>Content – developed a low-literacy comic book (*fotonovela)*<br><br>2. Language (Spanish)<br><br>Bicultural Community Educators (BCEs) – deliver the program<br><br>Gender and sexuality<br><br>Content-<br>primary goal of program is “clashing cultural norms” of immigrant cultures of participants and urban American culture<br><br>Partnership/capacity building - between research organisation and CBO (inferred)	1. Tres Hombres sin Fronteras – increased knowledge and improved attitudes as well as some reported behaviour change<br><br>2. SOLAAR –no results reported	1. No major limitation of intervention reported<br><br>2. Some methodological limitations reported in evaluation design	1. Tres Hombres sin Fronteras- Quasi-experimental<br><br>2. SOLAAR - Quasi-experimental	1. Related to Mishra (1996)<br><br>2. Related to Conner(2005)

First Author	Location	Intervention	Intervention participants	Intervention theory or model	Adaptation of intervention activities to increase cultural appropriateness	Key Results	Key Limitations	Study Design	Notes
Conner (2005)	California, USA	SOLAAR retreat for small groups with a focus on sexual risk reduction in immigrant gay men<br><br>Social Marketing<br><br>Various other activities including outreach and health promotion resources	Latino gay and bisexual men (mainly Mexican)<br><br>% immigrants – not stated but inferred to be >65%	Transtheoretical (Stages of Change)<br><br>Social Marketing	Language (Spanish)<br><br>Bicultural Community Educators (BCEs) – deliver the retreat program<br><br>Gender and sexuality<br><br>Content – primary goal of retreat program is "clashing cultural norms" of immigrant cultures of participants and urban American culture<br><br>Settings-advertising retreats Latino gay and bisexual publications in California<br><br>Partnership/capacity building - between research organisation and CBOs	Higher recruitment to retreats and increased potential for evaluation (no data)<br><br>Modification of program content	Some methodological limitations reported in evaluation design	Quasi-experimental<br><br>Participant evaluation surveys (pre/post)	Related to Conner (2004)

First Author	Location	Intervention	Intervention participants	Intervention theory or model	Adaptation of intervention activities to increase cultural appropriateness	Key Results	Key Limitations	Study Design	Notes
Darrow (2004)	Florida, USA	Reach 2010- Four key activities to raise awareness (not reported in detail)- outreach to residents and community leaders, mass media, Capacity building of CBOs using Abstinence, be Faithful, use Condoms (ABC) prevention messages with a focus on sexual risk reduction	African-American, Caribbean and Hispanic communities in 12 postal codes with elevated levels of HIV<br><br>Two telephone survey samples -each > 2000 respondents : Haitian (16-19%) Hispanic (25-29%) Caribbean (18-20%)<br><br>% immigrants – not stated but inferred to be >65%	PRECEDE/PROCEED model	Settings-targeting interventions to multiethnic Black and Hispanic neighbourhoods/communities informed by ethnographic assessments<br><br>Partnership/capacity building - between research organisation and three CBOs<br><br>Languages (Spanish and Creole) (inferred)<br><br>Content-focus groups and surveys with community groups to inform content of Community Action Plans	Between 2001 and 2002: significant increase in awareness of intervention activities, reported rates of HIV testing and reported participation in community prevention activities	No major limitation of interventions reported	Baseline and follow up random community telephone samples	Related to Hlaing (2006)
Diaz (1998)	San Francisco, USA	Hermanos de Luna Y Sol, a range of activities to recruit gay and bisexual immigrant men – with an emphasis on low-income, monolingual, unacculturated men- into small	122 Latino gay and bisexual gay men in small group educational activities<br><br>80% immigrants	Theory of self-regulation<br><br>Empowerment Theory<br><br>Psycho-cultural model of HIV risk (Diaz, 1998)	Bicultural Community Educators (BCEs) – to facilitate group activities<br><br>Language (Spanish)<br><br>Gender and sexuality – of group facilitators	Participants reported strongest effects of intervention activities in areas around HIV and safer sex and stronger perceived connectedness to	Not possible to assess effects of group activities post-intervention	Participant evaluation surveys	

		group education and peer support activities with a focus on sexual risk reduction			Content – incorporated dominant Latino cultural values on homosexuality in development of content<br><br>Settings-outreach and advertising to Latino gay and bisexual bars to recruit participants	Latino gay community		
Flaskerud (1988)	Los Angeles, USA	12-minute slide show with supporting health promotion resources with a focus HIV knowledge, attitudes and sexual risk reduction for immigrants (mainly refugees)	242 Vietnamese women and 74 men<br><br>98% immigrants	Not stated	Language (Vietnamese)<br><br>Bicultural Community Educators (BCEs) – Vietnamese educator delivered the session<br><br>Gender<br><br>Content- developed taking into account Vietnamese cultural values , Vietnamese imagery and characters<br><br>Settings-interventions delivered at a CBO targeting Indochinese refugees<br><br>Partnership/capacity building - between research organisation and refugee CBOs	No significant differences between the intervention and control groups in knowledge, attitudes or practices.<br><br>Significant differences in the intervention group in knowledge, attitudes or practices pre and post intervention.	Post-test surveys were immediately after the session<br><br>Possible mis-translation of some items on the pre/post questionnaire	Non-equivalent control groups design

First Author	Location	Intervention	Intervention participants	Intervention theory or model	Adaptation of intervention activities to increase cultural appropriateness	Key Results	Key Limitations	Study Design	Notes
Flaskerud (2000)	Los Angeles, USA	Various activities including - 1-1 counselling, group discussion, Spanish-language health promotion print and audio-visual resources - to reduce sexual and injecting drug use risks (not reported in detail) for low-income immigrant women	508 low-income Latina women (51 participated in qualitative evaluation of intervention)<br><br>% immigrants- not stated but inferred to be >65%	Cultural competence<br><br>Collaborative Inquiry	Language (Spanish)<br><br>Bicultural Community Educators (BCEs) – of health care workers who delivered the intervention in a women and children's health centre<br><br>Gender - of health care workers<br><br>Content- developed taking into account cultural values from focus groups and community consultations<br><br>Partnership/capacity building - between research organisation and women's health CBO	Qualitative results indicated that participants supported the use of using Latinos (same ethnicity and language) as a strategy to enhance the cultural competence of the intervention<br><br>Participants rated the intervention highly for:<br><br>clearing up misconceptions of HIV/AIDS & for resources developed and distributed as part of the intervention<br><br>the setting for the intervention (a women's health centre)<br><br>providing free HIV-antibody testing	Qualitative results indicated limitations which included:<br><br>having some mixed gender groups with male and female co-facilitators in some settings<br><br>using Latina women as health education 'messengers' to families was seen as problematic<br><br>Focus on CDC behavioural categories of HIV transmission for HIV education was seen as problematic by participants (e.g. the utility of condoms was in preventing pregnancy not HIV)	Qualitative data from focus groups	Related to Flaskerud (1997)

First Author	Location	Intervention	Intervention participants	Intervention theory or model	Adaptation of intervention activities to increase cultural appropriateness	Key Results	Key Limitations	Study Design	Notes
Flaskerud (1997)	Los Angeles, USA	Various activities including - 1-1 counselling, group discussion, Spanish-language health promotion print and audio-visual resources - to reduce sexual and injecting drug use risks (not reported in detail) for low-income immigrant women	508 low-income Latina women (191 participated in 12-month follow-up)<br><br>93% immigrants (44% Mexican-born)	Cultural competence	Language (Spanish)<br><br>Bicultural Community Educators (BCEs) – of health care workers who delivered the intervention in a women and children's health centre<br><br>Gender<br><br>Content- developed taking into account cultural values	Significant differences in the intervention group in knowledge, compared to control group at 12-months<br><br>Significant differences in the intervention group in condom use , compared to baseline at 12-months (not significant compared to control group)<br><br>Qualitative results indicated that participants supported the use of using Latinos (same ethnicity and language) as a strategy to enhance the cultural competence of the intervention	Qualitative results indicated limitations which included:<br><br>having some mixed gender groups with male and female co-facilitators in some settings<br><br>using Latina women as health education 'messengers' to families was seen as problematic<br><br>Focus on CDC behavioural categories of HIV transmission for HIV education was seen as problematic by participants (e.g. the utility of condoms was in preventing pregnancy not HIV)	Non-equivalent control groups design<br><br>Qualitative data from focus groups	Related to Flaskerud (2000)

First Author	Location	Intervention	Intervention participants	Intervention theory or model	Adaptation of intervention activities to increase cultural appropriateness	Key Results	Key Limitations	Study Design	Notes
Gomez (1999)	San Francisco, USA	Mujeres Unidas y Activas (MUA) - an intervention which aims to empower immigrant and refugee women and to assess effect on sexual risk reduction<br><br>MUA activities include support groups, workshops, HIV workshops and leadership training.<br><br>Participants at 6 months follow-up had attended a median of 9 MUA activities	94 low-income Latina women (74 participants in follow up)<br><br>100% immigrants	Empowerment Theory	Language (Spanish)<br><br>Bicultural Community Educators (BCEs) – of health care workers who delivered the intervention activities in a Latina CBO Gender - of health care workers<br><br>Content- developed taking into account cultural values (inferred)<br><br>Partnership/capacity building - between research organisation and Latina CBO	Significant differences at 6-months follow up in terms of :<br><br>increased comfort in sexual communication (with male partners)<br><br>a decrease in beliefs concerning traditional Latina gender roles<br><br>decreased coercive and violent behaviours by male partners	Some methodological limitations reported in evaluation design	Prospective study recruited over a 12-month period with follow-up at 3 and 6-months	

First Author	Location	Intervention	Intervention participants	Intervention theory or model	Adaptation of intervention activities to increase cultural appropriateness	Key Results	Key Limitations	Study Design	Notes
Haour-Knipe (1999)	Switzerland	Swiss Migrants Project –reporting on exploratory studies, process and outcome evaluations of three	Spanish, Portuguese and Turkish immigrants<br><br>980 outcome	Not stated	Languages (Spanish, Portuguese and Turkish)<br><br>Bicultural Community	Process evaluation: community “mediators” were successfully	Process Evaluation: very high turnover of community “mediators” due	Various qualitative and quantitative research methods	

	phases of the project<br><br>Various activities delivered – information dissemination to immigrant communities, HIV workshops, development of health promotion print and audio-visual resources, use of immigrant mass media, and some 1-1 support for PLWHA	evaluation surveys (441 Spanish, 385 Portuguese, 154 Turkish)<br><br>% of immigrants not stated but inferred to be >65%		Educators (BCEs) – three Community Co-ordinators and three teams of trained peer educators (“mediators”). Main activities of “mediators’ were: Workshops, HIV education integrated into their existing role (e.g. teaching English), supporting individual PLWHA<br><br>Content of activities- developed taking into account cultural values from exploratory needs analysis, and community consultations<br><br>Settings-delivering interventions via existing community structures in the Spanish, Portuguese and Turkish communities<br><br>Partnership/capacity building – building capacity of community “mediators” and existing community structures	engaging with the structures of each of the three communities to deliver a range of intervention activities<br><br>requests for activities from the three communities exceeded the capacity of the Community Co-ordinators and “mediators”<br><br>Outcome evaluation:<br><br>community “mediators” took on greater responsibilities<br><br>community “mediators” sometimes acted as community ‘change agents’<br><br>Knowledge, attitudes and protective behaviours were high and comparable to the general Swiss population	to migration Very high turnover of community “mediators” due to burnout associated with very high demand for activities from communities<br><br>Outcome evaluation:<br><br>poor definition of role and lack of standard remuneration of community “mediators”<br><br>Not enough horizontal contact between community “mediators”<br><br>Community “mediators” untrained and under-resourced to act a caregivers to PLWHA who were often extremely isolated in the community	including focus groups<br><br>Outcome evaluation included surveys respondents recruited via community ‘snowballing’ and clustering<br><br>Triangulation of survey data with other Swiss national data sets

First Author	Location	Intervention	Intervention participants	Intervention theory or model	Adaptation of intervention activities to increase cultural appropriateness	Key Results	Key Limitations	Study Design	Notes
Hlaing (2006)	Florida, USA	Evaluation of Reach 2010- Four key activities to raise awareness - outreach to residents and community leaders, mass media, capacity building of CBOs, using Abstinence, be Faithful, use Condoms (ABC) prevention messages in a multiethnic community	African-American, Caribbean and Hispanic communities in 12 postal codes with elevated levels of HIV<br><br>Three telephone survey samples -each > 1000 respondents : Haitian (14-19%) Hispanic (25-36%) Caribbean (17-20%)<br><br>% immigrants – not stated but inferred to be > 65%	PRECEDE/PROCEED model (inferred)	Settings-targeting interventions to multiethnic neighbourhoods/communities<br><br>Partnership/capacity building - between research organisation and three CBOs<br><br>Languages (Spanish and Creole)- in health promotion resources	Between 2001 and 2003: significant increase in perceptions of personal risk for HIV were reported in men (but not in women), abstinence from sexual activity declined, condom use increased	No major limitation of interventions reported	Baseline and follow up random community telephone samples	Related to Darrow (2004)
Hovey (2007)	Michigan, USA	*Informate* theatrical presentation to young immigrants in 7 migrant farmworker camps	71 Latino migrant farmworkers (mostly Mexican)<br><br>% immigrants – not stated but inferred to be > 65%	Not stated	Language (Spanish)<br><br>Bicultural Community Educators (BCEs) – Male/ female young theatre playersfrom the target groups and called “Teen Health Aides” (THAa)	Increased HIV/AIDS knowledge in pre-post surveys	Short-term knowledge gain may not have been sustained in participants	Pre/post survey with audience members	

					Content- THAs developed the script taking into account cultural values<br><br>Settings-delivered theatrical presentation at 7 farmworker migrant camps to enhance accessibility				
Kim (2006)	Chicago, USA	Project SEPA, 6 group sessions with a focus on sexual risk reduction for low-income immigrant women<br><br>Study examined intervention participants compared to dropouts to assess predictors of participation	214 Latina women participants (intervention group)<br><br>141 Latina women dropouts (control group)<br><br>Mexican 87% (intervention group), Mexican 72% (control group)<br><br>% of immigrants not stated but inferred to be > 65%	SCT (inferred)	Language (Spanish)<br><br>Bicultural Community Educators (BCEs) – group facilitators<br><br>Gender - of group facilitators<br><br>Content- of education sessions (inferred)<br><br>Settings-used a variety of methods to recruit participants including Spanish-language press, radio and television	Successful program completion was related to Mexican (rather than Puerto Rican) ethnicity, age (older), unemployment, level of depression and risk reduction intention, less drug use.<br><br>Dropout tended to occur before the intervention began, with a very high rate of retention among those who actually attended a session	Some methodological limitations reported in evaluation design	Cross sectional cohort study	Related to Peragallo (2005)

First Author	Location	Intervention	Intervention participants	Intervention theory or model	Adaptation of intervention activities to increase cultural appropriateness	Key Results	Key Limitations	Study Design	Notes
Kocken (2001)	The Netherlands	Single education session for male immigrants with a focus on HIV prevention and condom use	293 Turkish and Moroccan male immigrants<br><br>% of immigrants not stated but inferred to >65%	HBM	Language (Turkish and Arabic)<br><br>Bicultural Community Educators (BCEs) – peer educators were trained "lay heath advisors"<br><br>Gender - of "lay heath advisors"<br><br>Settings-used a variety to deliver education sessions incl. coffee houses, cafes and mosques	Decreased misunderstandings of HIV transmission and risk appraisal for HIV infection/improved<br><br>Between 60-70% of the intervention and control groups rated the importance of HIV education by a peer in their native language	Poorly educated immigrants were under-represented	RCT	
Loue (1996)	San Diego, USA	Establishment of a prevention program and organization to address HIV/AIDS among Asian and Pacific Islander immigrants- Project HAPI	1916 participants reached at outreach events<br><br>% of immigrants not stated but inferred to >65%	Model of locality development, social planning and social action [after Rothmann (1970)]	Content - developed taking into account cultural values from qualitative and quantitative needs analysis<br><br>Settings-use of "gatekeepers" to access communities<br><br>Partnership/capacity building – of community agencies in program planning and "political" meetings	Established a dedicated non-profit agency for Asian and Pacific Islanders to address HIV/AIDS<br><br>Network density analysis revealed a gradual increase in joint program development, co-ordination and mutual client referral	None reported	Descriptive	

First Author	Location	Intervention	Intervention participants	Intervention theory or model	Adaptation of intervention activities to increase cultural appropriateness	Key Results	Key Limitations	Study Design	Notes
Marahaj (1996)	UK	Assessment of a range of HIV prevention interventions with refugees and asylum seekers. Four summary case studies of interventions at group and community levels:<br>• African Communities HIV Project (ACHP)<br>• African Health Football Project<br>• Ugandan AIDS Action Fund Play<br>• Refugee Council's HIV and AIDS Development Work	All four case studies targeted African-born refugees and asylum seekers<br><br>Number of people reached varied for each intervention<br><br>% of immigrants not stated but inferred > 65% for all four interventions	No formal models stated	• 1. African Communities HIV Project<br>Partnership/capacity building – building alliances to create co-ordinated HIV prevention activities with African communities<br><br>• 2. African Health Football Project<br><br>Settings -integrated HIV health promotion into a soccer tournament<br><br>Bilingual Community Educators (BCEs) – Volunteer peer educators from 'Black' HIV services used to distribute HIV plan and staff the intervention	• African Communities HIV Project<br>Partnership was successful in attracting large numbers of African community members to health promotion events. Partnership was successful in bringing together prevention and care agencies.<br><br>• African Health Football Project<br>Reached 3,000 different people who attended the tournament, especially young men<br><br>Tournament helped to address taboo of discussing HIV/AIDS	• African Communities HIV Project<br>Diversity of African communities was a major challenge. Community 'politics' and personal and professional differences impacted on project sustainability.<br><br>• African Health Football Project<br>Securing funding from a range of local London authorities was an administrative challenge	Descriptive	

				Partnership/capacity building –tournament players and managers and ‘Black” volunteers planned the intervention	among African communities and address fears of racist reactions<br><br>Tournament developed into a self –sustaining institution and helped in the formation of ethnic-specific African agencies		
				• 3. Ugandan AIDS Action Fund Play<br><br>Bilingual Community Educators (BCEs) – commissioned a Ugandan theatre group to develop a play around HIV/AIDS<br><br>Content – commissioned a Ugandan theatre group to develop a play on HIV/AIDS<br><br>Settings- used a culturally appropriate and popular medium of communication to discuss HIV/AIDS & a variety of community settings for performance of the play	• Ugandan AIDS Action Fund Play<br>Over 300 people attended the performance of the play<br><br>The audience evaluation indicated that the play was well received	• Ugandan AIDS Action Fund Play<br>None reported	

				• 4. Refugee Council's HIV and AIDS Development Work<br><br>Language – HIV prevention resources developed and distributed in 8 refugee languages<br><br>Content – of training and seminars for HIV and refuges service providers designed to reflect HIV and refugee issues<br><br>Partnership/capacity building –providing organization support to refugee service providers for HIV work and linking refugee service providers with HIV service providers	• Refugee Council's HIV and AIDS Development Work<br><br>Between 15 and 50 attendees at workshops and seminars for service providers and very high satisfaction among attendees	• Refugee Council's HIV and AIDS Development Work<br>Difficulty in attracting refugee groups to workshops and seminars	

First Author	Location	Intervention	Intervention participants	Intervention theory or model	Adaptation of intervention activities to increase cultural appropriateness	Key Results	Key Limitations	Study Design	Notes
Martijn (2004)	Rotterdam, The Netherlands	Two studies of a single education session for male immigrants with a focus on HIV prevention and condom use<br><br>Study 1 – delivered by "lay health advisors"<br><br>Study 2- delivered by "lay health advisors" and "professional health advisors" (aided by an interpreter)	Study 1 – 75 mainly Turkish and Moroccan male and female immigrants<br><br>100% immigrants<br><br>Study 2- 36 Iraqi male refugees<br><br>100% immigrants	Not stated	Language (Turkish and Arabic)<br><br>Bicultural Community Educators (BCEs) – educators were trained "lay heath advisors" in both studies and interpreter was used in Study 2 with "professional health advisor"<br><br>Gender - of "lay heath advisors" and "professional health advisors"<br><br>Settings-used two settings. Study 1 followed a Newcomer Integration Project and Study 2 was delivered at a refugee reception centre	Study 1<br>Sig. increase pre/post in HIV knowledge and condom use intentions<br><br>Effect on social norms strongest among newly-arrived immigrants<br><br>Study 2<br>Sig. increase in HIV knowledge, stronger in "professional health advisor" participants, significant increase condom use intentions stronger in "lay health advisor" participants<br><br>Overall suggests that "lay" and "professional" health advisors "instigate different processes of change"	Study 2 Unable to recruit any female participants<br><br>Very small sample size	Study 1 Pre (2 weeks prior) and post (2 weeks after) surveys<br><br>Study 2 Similar survey pre/post but also "lay" or "professional" health advisors were compared	

First Author	Location	Intervention	Intervention participants	Intervention theory or model	Adaptation of intervention activities to increase cultural appropriateness	Key Results	Key Limitations	Study Design	Notes
Martin (2005)	Chicago, USA	Centro San Bonifacio HIV Prevention Program Education sessions carried out in the community for male and female immigrants with a focus on HIV knowledge and awareness	704 male and female Latino participants over 3 years:<br><br>Mexican 76% Central/South American 10%<br><br>% of immigrants not stated but inferred to be > 65%	Transtheoretical (Stages of Change)	Language (Spanish)<br><br>Bicultural Community Educators (BCEs) – "promotoras" were used to recruit and deliver education sessions<br><br>Settings-used a variety of settings to deliver education session including people's homes, schools, Church groups	Significant increase pre/post in HIV knowledge assoc. with being Central/South American and lower education score<br><br>Significant increase pre/post in and self-perceived risk for HIV<br><br>concern about AIDS in intervention group at 3-month follow-up	"Promotoras" are likely to have recruited participants through established referral networks<br><br>Many participants only partially completed the pre/post surveys which may have related to literacy, time constraints, or sensitivity around the questions	Pre/post surveys at education sessions	
Maxwell (2002)	Los Angeles, USA	Single education session to young immigrant attendees at a HIV clinic with a focus on condom use and sexual negotiation	106 immigrants (< 25yo) at baseline (54 attendees, 52 non-attendees): Central American 47% Mexican 40%<br><br>% of immigrants not stated but inferred to be > 65%	Not stated	Language (Spanish)	Significant increase in the number of session attendees reporting carrying condoms at 4 weeks follow up when compared to non-attendees	Participants were recruited at a HIV testing site	Pre/post surveys at 4 weeks among education session attendees and non-attendees	

First Author	Location	Intervention	Intervention participants	Intervention theory or model	Adaptation of intervention activities to increase cultural appropriateness	Key Results	Key Limitations	Study Design	Notes
Mc Mahon (2005)	Sydney and Melbourne, Australia	Pilot ethnic media advertising and publicity campaign targeting immigrants to promote HIV testing and access to treatment and care by PLWHA at public sexual health clinics	The readership/listenership of 14 language media were the target audiences<br><br>1067 attendees at 3 sexual health clinics<br><br>100% immigrants (HIV testing analysed against immigrant 'country of birth' data)	Social Marketing (inferred)	Languages (multiple)<br><br>Bicultural Community Educators (BCEs) – as media spokespeople on radio (inferred)<br><br>Content – campaign themes generated from a national needs assessment and refined with focus groups of BCEs<br><br>Settings-advertising and publicity used media accessed by immigrant populations	Increased number of HIV tests in the target audience clinic attendees pre/post campaign but not a statistically significant increase in the proportion of HIV tests carried out in the target audience	Campaign budget was small, HIV testing which may have occurred at other public or private clinics was not measured, there was a relatively short duration of the pre/post-campaign period of 3 weeks	Pre/post campaign using datasets on clinic attendees in 2000 and compared to a previous comparable period in 1999	
Mishra (1996)	California, USA	Tres Hombres sin Fronteras – two health promotion resources with a focus on sexual risk reduction for immigrant male farmworkers	150 Latino male farmworkers at baseline in experimental and control groups and 89 at 1-month follow-up<br><br>100% immigrants (all Mexican-born)	Not stated	Language (Spanish)<br><br>Content – developed a low-literacy comic book (*fotonovela)* and low-health literacy radio segments *(radionovela)*<br><br>Settings-outreach to farm camps to distribute resources	Increased knowledge and improved attitudes and some reported behaviour change<br><br>Low-literacy style of *fotonovela* appeared to be well understood by participants	*radionovela* did not reach participants despite distributing radios<br><br>Attrition of cohort, very small sample sizes & potential of response bias in self-reported condom use	Quasi experimental	Related to Conner (2004)

First Author	Location	Intervention	Intervention participants	Intervention theory or model	Adaptation of intervention activities to increase cultural appropriateness	Key Results	Key Limitations	Study Design	Notes
Nakyonyi (1993)	Toronto, Canada	Africans United to Control AIDS (AUCA)<br><br>Various activities described including information dissemination to African immigrants, HIV workshops, outreach to African community organisations, use of immigrant mass media	African immigrant communities (mainly refugees)<br><br>% immigrants not stated but inferred to be >65%	Community Development (inferred)	Languages (multiple)<br><br>Bilingual Community Educators (BCEs) – African volunteers recruited and trained as community educators/outreach workers<br><br>Content – activities are developed in partnership with community groups (inferred)<br><br>Settings-use of community events and venues and use of media accessed by African immigrant populations	Various types of activities described as being effective<br><br>High acceptability of most activities among African immigrants despite high levels of stigma associated with the mainstream Canadian media portrayal of HIV/AIDS as an "African disease"	No evaluation data reported<br><br>African immigrants reported to be more concerned with "struggling for survival"<br><br>Heterogeneity of African immigrants in terms of ethnicity, country of origin, religion and other beliefs/attitudes	Descriptive	
Organista (2006)	Mexico border area, USA	Two pilot education sessions for Latino Migrant Day Labourers with a focus on sexual risk reduction, condom use and reduced alcohol and substance use	23 Latino men (mostly Mexican)<br><br>70% Mexican 26% Central American<br><br>% immigrants not stated but inferred to be > 65%	HBM<br><br>Freire's participatory education model	Language (inferred) (Spanish)<br><br>Content- education sessions developed from formative research, use of *Lotteria cards*, *fotonovelas*, comic-style books, and *radionovelas* radio soap operas	Contextual issues dominated participants' risk reduction concerns such as migration-related stressors which hindered migration goals (sending money to support family in Mexico)	Very small sample size (only 12 participants completed post survey)	Pre-post education session (4 weeks)<br><br>Qualitative analysis of participants responses to the second education session	

						Participants' concerns about HIV risks mainly related to the use of female sex workers in the USA vs wives/girlfriends in Mexico<br><br>Some gains in condom use intentions and knowledge of condom use at 4 weeks follow-up			
Peragallo (2005)	Chicago, USA	Project SEPA, 6 group sessions with a focus on sexual risk reduction for low-income immigrant women	404 Latina women<br><br>Mexican 89%, Puerto Rican 10% in intervention group<br><br>% of immigrants not stated but inferred to be > 65%	SCT	Language (Spanish)<br><br>Bicultural Community Educators (BCEs) – group facilitators<br><br>Gender - of group facilitators<br><br>Content- of education sessions (inferred)<br><br>Settings-used a variety of methods to recruit participants including Spanish-language press, radio and television	Significant differences at 3-months and 6-months follow-up was highest for HIV knowledge<br><br>Significant differences at 6-months follow up including: partner communication<br><br>risk reduction intentions<br><br>perceived barriers to condom use and reported condom use	Some methodological limitations reported in evaluation design	RCT	Related to Kim (2006)

First Author	Location	Intervention	Intervention participants	Intervention theory or model	Adaptation of intervention activities to increase cultural appropriateness	Key Results	Key Limitations	Study Design	Notes
Raj (2001)	Boston, USA	Comparison of two interventions to reach low-income immigrant women:<br><br>1. HIV-specific with participatory education;<br>2. women's health specific and didactic<br><br>Each intervention 12 group sessions & 16 hours of content- with a focus on sexual risk reduction	170 Latina women<br><br>89% immigrants<br><br>Dominican 55%, Puerto Rican 13%, Mexican and Central American 13%	Intervention 1- Social Cognitive Theory (SCT)<br><br>Empowerment Theory<br><br>Intervention 2<br><br>SCT<br><br>Theory of Reasoned Action (TRA)<br><br>Health Belief Model (HBM)	Language (Spanish)<br><br>Bicultural Community Educators (BCEs) – to facilitate groups<br><br>Content- of education sessions (inferred)<br><br>Gender - of group facilitator	Increased reported use of, and intentions to use, condoms compared to control<br><br>Increased sexual negotiation in HIV group only<br><br>Increased HIV testing in women's health group only	Study Design<br><br>Only cost-effective if intervention did prevent HIV transmission<br><br>Did not focus on male partners of participants	Quasi-experimental	3& 15 month follow up in Amaro (2002)
Shtarkshall (2000)	Israel	Overview of model for the development of HIV/AIDS programs for immigrants using two programs for immigrants as case studies:<br><br>1. Immigrant youth from the former Soviet Union and Ethiopia<br><br>2. Ethiopian immigrants	Immigrant youth – principally from the former Soviet Union and Ethiopia<br><br>Ethiopian immigrants<br><br>% of immigrants not stated in each case study but inferred to be >65%	Model is proposed which has many related facets to develop a HIV/AIDS program for immigrants	Model proposes an iterative approach to program development including:'old-country' and 'new-country' values; cultural transitions of immigration; employing immigrant 'cultural insighters'; harnessing leadership from the immigrant community; developing culturally specific messages; training of health care workers	No formal results of immigrant youth program reported<br><br>No formal results of Ethiopian immigrant program reported	No budget for evaluation of immigrant youth program<br><br>No time to plan effectively for Ethiopian immigrant program as they had been brought to Israel at very short notice due to a political crisis	Not a formal study rather a best practice report	Related to Chemtov (1993) & Soskolne (2002)

First Author	Location	Intervention	Intervention participants	Intervention theory or model	Adaptation of intervention activities to increase cultural appropriateness	Key Results	Key Limitations	Study Design	Notes
Somerville (2006)	California and Texas, USA	Training popular opinion leaders to carry out HIV outreach among immigrant men who have sex with men	37 Young Latino Promotores (YPLs) - outreach workers<br><br>766 Latino male respondents completed Year 1 and year 2 of evaluation surveys<br><br>% of immigrants not stated but inferred to be > 65%	Theory of diffusion<br><br>Popular Opinion Leader-after Kelly (1986)	Popular Opinion Leader intervention adapted after a community needs analysis in terms of:<br><br>Language (Spanish) – of training materials and evaluation surveys<br><br>Bicultural Community Educators (BCEs) – YPLs to conduct outreach and Latino program co-ordinators at the two sites<br><br>Gender and sexuality - of YPLs (male and female)<br><br>Content – of intervention training of YPLs modified to:<br>• build on Latino cultural understandings of *promotoras*<br>• address higher HIV information needs of Latino men who have	YPLs reported 2,376 educational contacts over two years<br><br>766 pre and post surveys completed over two years<br><br>In general no changes IN HIV knowledge reported pre and post<br><br>Statistically significant changes reported post intervention in terms of one HIV social norm measure<br><br>Statistically significant changes reported post intervention in terms of use of condoms if receptive partner	Evaluation findings may have been confounded by other HIV interventions with the target group<br><br>The target groups mobility was a challenge for YPLs<br><br>Theory of diffusion suggests that to achieve population change 15% of the population need to trained as YPLs	Cross – sectional convenience sample (evaluation surveys) pre and post (2 months) of YPLs training over two years	

					sex with men<br>• culturally appropriate communication styles<br>• place of sexual orientation in YPLs lives<br>• referral skills<br><br>Settings-implemented intervention in locales known to have high numbers of Latino agricultural workers				
Soskolne (2002)	Israel	Overview of model for the development of HIV/AIDS programs for immigrants using several programs examples as case studies:<br><br>1. Immigrant youth from the former Soviet Union and Ethiopia<br><br>2. Ethiopian immigrants	Immigrant youth – principally from the former Soviet Union and Ethiopia<br><br>Ethiopian immigrants<br><br>% of immigrants not stated in each case study but inferred to be >65%	Model is proposed which has many related components to develop a HIV/AIDS program for immigrants which includes macro, intermediate, and individual levels	Model proposes an iterative approach to program development, which involves immigrants as 'cultural insighters' and mediators, to develop programs which were specific to immigrant youth and the general population of Ethiopian immigrants.	Structural changes in several key health policies<br><br>Other initiatives' outcomes reported descriptively in brief summaries	Structural interventions were unable to address higher social determinants of health among immigrants<br><br>Intervention to reach the general population of Ethiopian immigrants failed to address stigmatization by the Israeli public of Ethiopians as HIV 'carriers'	Not a formal study rather a best practice model	Related to Chemtov (1993) & Shtarkshall (2000)

First Author	Location	Intervention	Intervention participants	Intervention theory or model	Adaptation of intervention activities to increase cultural appropriateness	Key Results	Key Limitations	Study Design	Notes
Van Servellen (2003)	Los Angeles, USA	Evaluation of a small group intervention –*Es por la vida* -over 5 weeks to improve health literacy and treatment adherence among low-income Spanish-speaking HIV-positive Latinos	Predominantly male HIV-positive groups-41 intervention participants and 40 comparison group participants<br><br>>90% of groups were immigrants	Not stated	Language (Spanish)<br><br>Bicultural Community Educators (BCEs) – group facilitators<br><br>Content- of education sessions using Latino cultural values	At 6-week follow-up intervention group had higher HIV knowledge but no difference in reported adherence to treatment between the intervention and comparison group	Small sample size and short duration of follow-up at 6 weeks<br>No changes in self-reported adherence	Quasi-experimental	
Wolfers (2007)	Rotterdam, The Netherlands	Study of the development of STI/HIV interventions for heterosexual immigrant men to increase condom use with casual and new partners	Afro-Caribbean men<br><br>Turkish and Moroccan unmarried men<br><br>% of immigrants not stated but inferred to be >65%	Intervention Mapping (to develop intervention)<br><br>Intervention theories were: SCT Implementation Intentions Theory Inoculation Theory	Bicultural Community Educators (BCEs) – in intervention development group and recruiters for focus groups of needs analysis and focus testing of intervention<br><br>Content- of intervention responded to different socio-cultural determinants around condom use, intervention plan and resources were focus-tested with Afro-Caribbean, and Turkish and Moroccan men	Two different culturally grounded interventions were developed for heterosexual immigrant men:<br><br>a 1-1 outreach intervention for Afro Caribbean men with a playing cards resource<br><br>a small group intervention for Turkish and Moroccan men with posters and content for groups	The needs analysis phase only used focus-groups to gather data and recruitment was problematic<br><br>The Intervention Mapping protocol was somewhat time-consuming and had to be adapted<br><br>Interventions have only been designed, not implemented	Intervention Mapping	

First Author	Location	Intervention	Intervention participants	Intervention theory or model	Adaptation of intervention activities to increase cultural appropriateness	Key Results	Key Limitations	Study Design	Notes
Worth (2003)	New Zealand	National HIV/AIDS Refugee Health Education Program which comprised training BCEs , developing community-specific action plans, workshops, resource development, support for HIV-positive people	Sub-Saharan African male and female refugees<br><br>100% immigrants	Tuelimishane model- "lets learn together"	Language – various African languages used in workshops<br><br>Bicultural Community Educators (BCEs) – from a range of Sub-Saharan African backgrounds<br><br>Content- of education workshops and health promotion resources- used ABC<br><br>Settings-used a variety of community settings for workshops and developed community HIV/AIDS health promotion action plans	137 BCEs trained<br><br>32 community HIV/AIDS health promotion action plans developed<br><br>HIV/AIDS health promotion module, poster and health promotion manual<br><br>Workshops delivered and evaluated (inferred)<br><br>Some "support cells" developed for Sub-Saharan African HIV-positive refugees	Program focused on modifying individual level factors for HIV prevention and so could not address wider structural or environmental factors<br><br>Poor uptake of support mechanisms for HIV-positive people due to reluctance to participate in groups	Descriptive study of a program	

Adapted from (Darbes, Kennedy, & Rutherford, 2005)

Darbes, L., Kennedy, G., & Rutherford, G. (2005). *Systematic review of behavioural prevention interventions to prevent HIV infection in communities of color*. Retrieved 3 May, 2008, from http://hivinsite.ucsf.edu/
