# Supplementary material for: HIV among immigrants living in high-income countries: a realist review of evidence to guide targeted approaches to behavioural HIV prevention
Source: Syst Rev. 2012 Nov 20;1:56. doi: 10.1186/2046-4053-1-56 (PMC3534573; doi:10.1186/2046-4053-1-56)
Supplement: Additional file 5 — Collation of views studies included in the review. (PDF 342 kb) [file 2046-4053-1-56-S5.pdf]

Collation of views studies included in the review

First Author	Location	Study Aims	Study participants	Study Design – sampling and data collection methods	Study Design –data analysis methods	Sensitivity and appropriateness of study methods to allow participants to express their views	Notes
Anderson (2004)	London, UK	Explore the lived experiences of immigrant women with HIV	62 African-born women<br><br>100% immigrants	Purposive sample<br>Brief quantitative survey<br>followed by 1-1 in-depth interviews	Thematic analysis	Female interviewers<br>Interviews in French or English<br>Survey administered orally if required	Related to Doyal (2003), Doyal (2005), Doyal (2006)
Apostolopoulos (2006)	Arizona & South Carolina, USA	Examine intertwined individual and environmental factors in relation to HIV/STI risks among male –gay and straight- immigrant farm workers	23 Mexican migrant farm workers<br><br>100% immigrants	Snowball sample<br>1-1 in-depth interviews	Thematic analysis	Interviews in Spanish<br>No tape recording or written consent due to sensitivity of undocumented immigration status of some participants	
Ayala (2001)	Miami, New York City and Los Angeles, USA	Analyze the relationship between race, class, and unprotected sex among immigrant gay men	293 Latino gay men<br><br>75% immigrants	Convenience sample in 3 cities<br>28 focus groups followed by brief quantitative survey	Thematic analysis	Participants offered choice of focus groups in English or Spanish	Related to Diaz (1999)<br>Diaz (2000)
Bhattacharya (2004)	New York City, USA	Explore social capital resources and influences on HIV risk behaviours among immigrant heterosexual men	17 immigrant Asian Indian immigrant heterosexual men<br><br>100% immigrants	Purposive sample<br>1-1 in-depth interviews	Grounded theory	Not stated	

First Author	Location	Study Aims	Study participants	Study Design – sampling and data collection methods	Study Design –data analysis methods	Sensitivity and appropriateness of study methods to allow participants to express their views	Notes
Castro-Vazquez (2006)	Japan	Explore the relationship between HIV/AIDS and 'community' support for immigrants with HIV	20 HIV-positive Latin Americans- mostly Nikkejin of Japanese ancestry<br><br>100% immigrants	Snowball sampling<br><br>1-1 in-depth interviews	Systemic networks analysis	Interviews in Spanish and Portuguese	
Chin (1999)	Los Angeles, USA	Explore how immigrant women assess HIV risk in social interactions	21 Asian and Pacific Islander women<br><br>75% immigrants	Purposive sampling<br><br>1-1 in-depth interviews	Grounded theory	Interviews were conducted by a female researcher from an Asian and Pacific Islander background	
Chin (2007)	New York City, USA	Explore the experiences and needs of immigrants living with HIV/AIDS	35 HIV-positive Asian and Pacific Islanders- qualitative arm<br><br>89 HIV-positive Asian and Pacific Islanders- survey<br><br>> 90% immigrants	Focus Groups<br><br>1-1 in-depth interviews<br><br>Surveys	Thematic analysis	Interviews and some focus groups were conducted in community languages<br><br>Interpreters were offered/used	
Chinouya (2006)	Milton Keynes, UK	Explore the role of faith, family life, service utilization and coping among immigrants living with HIV	22 heterosexual HIV-positive Africans -17 females and 5 males<br><br>20 faith leaders<br><br>100% immigrants	Purposive sampling (inferred)<br><br>1-1 in-depth interviews<br><br>Focus groups<br><br>Quantitative survey (not reported)	Thematic analysis	1-1 interviews enhanced confidentiality of HIV-positive participants	

First Author	Location	Study Aims	Study participants	Study Design – sampling and data collection methods	Study Design –data analysis methods	Sensitivity and appropriateness of study methods to allow participants to express their views	Notes
Dawson (2001)	Melbourne, Australia	Explore immigrant women's gender and sexual identities in relation to HIV and sexual health	50 women-25 born in Chile and 25 born in El Salvador<br><br>100% immigrants	Snowball sampling<br><br>1-1 in-depth interviews	Grounded theory	Most interviews in Spanish	Related to Dawson (2003)
Dawson (2003)	Melbourne, Australia and Santiago, Chile	Explore past and present social changes on the life experiences related to HIV/AIDS among immigrant women and women in the 'home' country	25 immigrant women born in Chile<br><br>16 women in Chile<br><br>100% immigrants (Melbourne)	50 women-25 born in Chile and 25 born in El Salvador<br><br>100% immigrants	Grounded theory	Not stated	Related to Dawson (2001)
Diaz (1999)	New York, Los Angeles & Miami, USA	Explore high rates of unprotected anal intercourse among immigrant gay and bisexual men	293 Latino gay and bisexual men<br><br>75% immigrants	24 focus groups	Thematic analysis (inferred)	Participants were stratified according to age and preferred language	Related to Diaz (2000) Ayala (2001)
Diaz (2000)	New York, Los Angeles & Miami, USA	Explore poverty, racism and homophobia and their impact on sexual risks, substance use and psychological distress among immigrant gay and bisexual men	912 Latino gay men<br><br>73% immigrants	Survey based on extensive qualitative research	Not stated	Not stated	Related to Diaz (1999) Ayala (2001)

First Author	Location	Study Aims	Study participants	Study Design – sampling and data collection methods	Study Design –data analysis methods	Sensitivity and appropriateness of study methods to allow participants to express their views	Notes
Dodds (2006)	UK	Explore social discourses around HIV-related stigma and discrimination among a range of people including immigrants living with HIV	125 participants - 44 gay/bisexual men, 34 heterosexual African men & 41 heterosexual African women<br><br>100% immigrants (African participants)	20 'mixed' focus groups	Thematic analysis	Used African-born facilitators in groups that were 'mixed'	Related to Dodds (2004)
Dodds (2004)	UK	Explore social discourses around HIV-related stigma and discrimination among a range of people including immigrants living with HIV	125 participants - 44 gay/bisexual men, 34 heterosexual African men, 41 heterosexual African women 100% immigrants (African participants)	20 'mixed' focus groups	Thematic analysis	Used African-born facilitators in groups that were 'mixed'	Related to Dodds (2006)
Doyal (2003)	London, UK	Explore the lived experiences of immigrant women with HIV	62 African-born women<br><br>100% immigrants	Purposive sample Brief survey followed by 1-1 in-depth interviews	Thematic analysis (inferred)	Not stated	Related to Anderson (2004), Doyal (2005), Doyal (2006)
Doyal (2005)	London, UK	Explore the lived experiences of immigrant women with HIV	62 African-born women<br><br>100% immigrants	Purposive sample Brief survey followed by 1-1 in-depth interviews	Thematic analysis (inferred)	Female interviewers Interviews in French or English Survey administered orally if required	Related to Doyal (2003), Anderson (2004), Doyal (2006)
Doyal (2006)	London, UK	Explore the lived experiences of immigrant women with HIV	62 African-born women<br><br>100% immigrants	Purposive sample(inferred) Brief survey followed by 1-1 in-depth interviews	Thematic analysis (inferred)	Not stated	Related to Doyal (2003), Doyal (2005), Anderson (2004)

First Author	Location	Study Aims	Study participants	Study Design – sampling and data collection methods	Study Design –data analysis methods	Sensitivity and appropriateness of study methods to allow participants to express their views	Notes
Flaskerud (1991)	Los Angeles, USA	Explore HIV/AIDS health beliefs, and their relationship to 'traditional' beliefs, among low-income immigrant women	59 low-income Latinas<br><br>70% immigrants	Convenience sampling<br><br>17 focus groups	Content analysis	Bilingual facilitators in focus groups	
Flaskerud (1996)	Los Angeles, USA	Explore sexual practices, attitudes and knowledge related to HIV transmission among low-income immigrant women	508 low-income Latinas(surveys)<br><br>55 low-income Latinas (focus groups)<br><br>91% immigrants (surveys)<br>% immigrants in focus groups not stated but inferred to be >70%	Convenience sampling<br><br>Surveys carried out followed by focus groups	Content analysis<br><br>Surveys analysed and focus groups used to clarify preliminary quantitative findings	Spanish and English surveys<br><br>Bilingual facilitators in focus groups	
Gifford (1998)	Melbourne, Australia	Explore ways in which immigrant women understand risks to sexual health including HIV	60 women - 20 Chilean, 20 Turkish, 20 second generation Greek<br><br>90% immigrants(Chilean and Turkish participants)	Purposive, snowball sampling<br><br>1-1 in-depth interviews	Content analysis	Interviews conducted by ethnically matched bilingual interviewers mostly in people's homes	

First Author	Location	Study Aims	Study participants	Study Design – sampling and data collection methods	Study Design –data analysis methods	Sensitivity and appropriateness of study methods to allow participants to express their views	Notes
Hirsch (2002)	Atlanta, USA and Mexico	Explore social constructions of HIV risks among immigrant women and women in the 'home' country	26 Mexican women -13 in Atlanta & 13 in Mexico<br><br>100% immigrants(Atlanta)	Sampling not stated<br><br>1-1 in-depth interviews	Thematic analysis	Not stated	
Jemmott (1999)	Penn., USA	Explore perceptions of HIV risks and culturally appropriate considerations for HIV prevention among immigrant women	30 Asian and Pacific Islander women – 22 community members and 8 service providers<br><br>100% immigrants	Snowball sampling<br><br>3 focus groups	Thematic analysis	Interpreters available in focus groups	
Kang (2003)	New York City, USA	Explore cultural attitudes, and HIV-related behaviours and perceptions among undocumented immigrants living with HIV	16 HIV-positive Asian and Pacific Islander immigrants<br><br>100% immigrants	Convenience sampling<br><br>1-1 in-depth interviews	Thematic analysis	Focus group with undocumented immigrants living with HIV was used to develop 1-1 interview guide<br><br>Interviews conducted in multiple Asian languages	
Korner (2007a)	Sydney, Australia	Explore the lived experience of immigrants living with HIV/AIDS – residency issues	29 participants living with HIV (men and women)<br><br>96% immigrants	Convenience sampling<br><br>1-1 in-depth interviews	Thematic analysis	Interviews conducted in participant's language of preference<br><br>Interpreters and/or bilingual support workers were present	Related to Korner (2007b) Korner (2007c) Korner (2005)
Korner (2007b)	Sydney, Australia	Explore the lived experience of immigrants living with HIV/AIDS –late HIV diagnosis	29 participants living with HIV (men and women)<br><br>96% immigrants	Convenience sampling<br><br>1-1 in-depth interviews	Thematic analysis	Interviews conducted in participant's language of preference<br>Interpreters and/or bilingual support workers were present	Related to Korner (2007a) Korner (2007c) Korner (2005)

First Author	Location	Study Aims	Study participants	Study Design – sampling and data collection methods	Study Design – data analysis methods	Sensitivity and appropriateness of study methods to allow participants to express their views	Notes
Korner (2007c)	Sydney, Australia	Explore the lived experience of immigrants living with HIV/AIDS – disclosure issues	29 participants living with HIV (men and women)<br><br>96% immigrants	Convenience sampling<br><br>1-1 in-depth interviews	Thematic analysis	Interviews conducted in participant's language of preference<br><br>Interpreters and/or bilingual support workers were present	Related to Korner (2007a) Korner (2007b) Korner (2005)
Korner (2005)	Sydney, Australia	Explore the lived experience of immigrants living with HIV/AIDS	29 participants living with HIV (men and women)<br><br>96% immigrants	Convenience sampling<br><br>1-1 in-depth interviews	Thematic analysis	Interviews conducted in participant's language of preference<br><br>Interpreters and/or bilingual support workers were present	Related to Korner (2007a) Korner (2007b) Korner (2007c)
McQuiston (1998)	North Carolina, USA	Explore the preventive practices for HIV and STIs of newly arrived immigrants	31 Hispanic participants (men and women)<br><br>100% immigrants	Convenience sampling<br><br>3 focus groups (men)<br>3 focus groups(women)	Content analysis	Bilingual facilitators in focus groups<br><br>Facilitators in focus groups matched by gender	Related to McQuiston (2000)
McQuiston (2000)	North Carolina, USA	Explore the preventive practices for HIV of newly arrived immigrants	31 Hispanic participants (men and women)<br><br>100% immigrants	Convenience sampling<br><br>3 focus groups (men)<br>3 focus groups(women)	Content analysis	Bilingual facilitators in focus groups<br><br>Facilitators in focus groups matched by gender<br><br>Credibility of study findings checked in informal interviews with community members	Related to McQuiston (1998)

First Author	Location	Study Aims	Study participants	Study Design – sampling and data collection methods	Study Design – data analysis methods	Sensitivity and appropriateness of study methods to allow participants to express their views	Notes
Moreno (2007)	New York City, USA	Explore the contextual risks of HIV-positive status and domestic violence against immigrant women	32 Latina women living with HIV<br><br>65% immigrants	Convenience sampling<br><br>3 focus groups<br>5 1-1 in-depth interviews	Thematic analysis	Bilingual facilitator in focus groups<br><br>Facilitators in focus groups matched by gender<br><br>Preliminary themes presented to community members and peers to validate findings	
Nemoto (2005)	San Francisco, USA	Explore the cognitive, cultural and contextual factors that influence HIV-related risk behaviours among female immigrants who engage in sex work at massage parlours and owners/managers	43 masseuses – mostly Thai and Vietnamese<br><br>8 parlour owners/managers<br><br>100% immigrants (masseuses and owners/managers)	Convenience sampling<br><br>8 focus groups(masseuses)<br><br>1-1 in-depth interviews (parlour owners/managers)	Thematic analysis	Mapping of suburbs where massage parlours were located by outreach workers<br><br>Focus groups stratified by ethnicity and language<br><br>Focus groups carried out in Thai or Vietnamese<br><br>Parlour owners/managers also interviewed in participant's language of preference	
Poon (2002)	Toronto, Canada	Explore the cultural and social barriers which influence HIV-related risks among gay, lesbian, and bisexual young immigrants	15 Asian young people<br><br>14 immigrant participants (95%)	Convenience sampling<br><br>3 focus groups<br><br>Some 1-1 in-depth interviews	Thematic analysis	Pilot focus group conducted prior to the study<br><br>Preliminary findings sent to an advisory group of community members for feedback	
Rhodes (2007)	North Carolina, USA	Explore cultural and social determinants of HIV risk and identify potential interventions among immigrant men	70 Latino men<br><br>100% immigrants- mostly Mexican	Convenience sampling<br><br>7 focus groups	Thematic analysis	Bilingual facilitators in focus groups<br>Interview guide developed iteratively in Spanish<br>Recruitment of participants through a Latino soccer club	

First Author	Location	Study Aims	Study participants	Study Design – sampling and data collection methods	Study Design – data analysis methods	Sensitivity and appropriateness of study methods to allow participants to express their views	Notes
Shedlin (2005)	New York State, USA	Explore social and behavioural factors influencing HIV risk among newly arrived (<1 year) immigrants	86 Latinos participated in focus groups<br><br>51 Latinos participated in 1-1 depth interviews<br><br>100% recent immigrants	Snowball sampling<br><br>11 focus groups<br><br>1-1 in-depth interviews	Thematic analysis	Research team conducted filed activities prior to study to ally fear of immigration authorities<br><br>Focus groups were stratified by nationality, sex and site<br><br>One key informant focus group and interviews were conducted	
Shedlin (2002)	New York City, USA	Explore predominant cultural influences social regarding sex work, drug use and HIV/AIDS among immigrants with very high risks for HIV/AIDS	20 Dominicans involved in drug-related or sex work-related activities<br><br>% immigrants not stated but inferred to be >70%	Purposive sampling<br><br>1-1 in-depth interviews	Thematic analysis	Interviews conducted in participant's language of preference –all Spanish<br><br>Emerging themes complemented findings from quantitative data	A qualitative arm following a larger quantitative survey in the same community
Shedlin (2004)	New York City, USA	Explore perceptions, beliefs experiences and knowledge of care issues for immigrants –most of whom were living with HIV	57 Latino men and women - mainly PLWHA<br><br>100 % immigrants	Convenience sampling<br><br>Focus groups	Thematic analysis	Focus groups were stratified by ethnicity and sex	

First Author	Location	Study Aims	Study participants	Study Design – sampling and data collection methods	Study Design –data analysis methods	Sensitivity and appropriateness of study methods to allow participants to express their views	Notes
Steel (2003)	Sweden	Explore the relationship of pre-migrations trauma on HIV risk behaviour among refugees post-settlement and explore the development of HIV prevention interventions	122 Sub-Saharan African refugees<br><br>100% immigrants	Snowball sampling<br><br>Focus groups<br><br>1-1 in-depth interviews	Thematic analysis	Participant's had the opportunity to choose the language, ethnicity and gender of the interviewer	
Wilson (2004)	New York City, USA	Explore experiences of, and responses to, social discrimination among gay immigrants	23 Asian and Pacific Islander gay men whose narratives of 166 episodes of discrimination were analysed<br><br>91% immigrants	Purposive sampling<br><br>1-1 in-depth interviews	Thematic analysis	None stated	
Yoshikawa (2003)	Northeastern city, USA	Explore the experiences of immigrant peer educators to develop culturally anchored theories of HIV prevention among their immigrant target populations	35 Asian and Pacific Islander peer educators<br><br>89% immigrants	Focus groups	Grounded Theory	Focus groups were stratified by target groups (e.g. youth, gay men, heterosexual men) that they worked with	

First Author	Location	Study Aims	Study participants	Study Design – sampling and data collection methods	Study Design –data analysis methods	Sensitivity and appropriateness of study methods to allow participants to express their views	Notes
Zuniga (2006)	San Diego/ Tijuana, USA/Mexico border	Explore barriers and facilitators to service utilisation by HIV-positive immigrants	9 HIV-positive Latino gay men<br>9 HIV-positive Latina women<br><br>100% immigrants (inferred)	Focus groups	Content analysis	Bilingual facilitators in focus groups<br><br>Focus groups conducted in preferred first language (Spanish)<br><br>Coding of transcripts carried out in first language before being translated into English	

Adapted from (Rees et al., 2004) and (Darbes, Kennedy, & Rutherford, 2005)

Darbes, L., Kennedy, G., & Rutherford, G. (2005). *Systematic review of behavioural prevention interventions to prevent HIV infection in communities of color*. Retrieved 3 May, 2008, from http://hivinsite.ucsf.edu/

Rees, R., Kavanagh, J., Burchett, H., Sheperd, J., Brunton, G., Harden, A., et al. (2004). *HIV health promotion and men who have sex with men: A systematic review of research relevant to the development and implementation of effective and appropriate interventions*. Retrieved 15 October, 2006, from www.eppi.ioe.ac.uk
