# Supplementary material for: HIV among immigrants living in high-income countries: a realist review of evidence to guide targeted approaches to behavioural HIV prevention
Source: Syst Rev. 2012 Nov 20;1:56. doi: 10.1186/2046-4053-1-56 (PMC3534573; doi:10.1186/2046-4053-1-56)
Supplement: Additional file 6 — Annotation of intervention studies: Summary against adaptive mechanisms. [file 2046-4053-1-56-S6.pdf]

Annotation of intervention studies: Summary against adaptive mechanisms

Key: sound (+++), moderate (++), partial (+) or no evidence (X)

First Author & Location	Intervention	Adaptation of intervention activities to increase cultural appropriateness	Mechanisms of adaptation 'authenticity'	Mechanisms of adaptation 'understanding '	Mechanisms of adaptation 'consonance'	Mechanisms of adaptation 'specificity '	Mechanisms of adaptation 'embeddedness'	Mechanisms of adaptation 'endorsement '	Mechanisms of adaptation 'framing'
**Amaro (2002) Boston, USA**	Comparison of two interventions for immigrant women:<br>1. HIV-specific with participatory education;<br>2. women's health specific and didactic<br><br>Each intervention 12 group sessions & 16 hours of content - focus on sexual risk reduction	Language (Spanish)<br><br>Bilingual Community Educators (BCEs) – to facilitate groups<br><br>Gender - of facilitator<br><br>Content-used 'cultural elements' in curriculum	1. +++<br><br>2. +++	1. +++<br><br>2. +++	1. +++<br><br>2. +++	1.++<br><br>2. ++	1. ++<br><br>2.+	1.X<br><br>2. X	1. X<br><br>2. X
**Carballo -Dieguez (2005)**<br><br>**New York City, USA**	8 group sessions with a focus on sexual risk reduction for immigrant gay men	Language (Spanish)<br>Bilingual Community Educators (BCEs)<br>Gender & sexuality - of facilitator<br>Content – use of 'dichos' Spanish proverbs	+++	+++	+++	++	++	++	+

First Author & Location	Intervention	Adaptation of intervention activities to increase cultural appropriateness	Mechanisms of adaptation 'authenticity'	Mechanisms of adaptation 'understanding'	Mechanisms of adaptation 'consonance'	Mechanisms of adaptation 'specificity'	Mechanisms of adaptation 'embeddedness'	Mechanisms of adaptation 'endorsement'	Mechanisms of adaptation 'framing'
**Chemtov (1993) Israel**	200 group sessions to raise awareness of HIV/AIDS in a newly arrived immigrant community<br><br>A range of supporting activities (e.g. health promotion resources) to reach 24,000 newly arrived immigrants	Language (Amharic)<br><br>Bilingual Community Educators (BCEs) – trained 34 'cultural mediators' to deliver a program in various ways –mainly education sessions<br><br>Content – use of popular proverbs<br><br>Settings- sessions were delivered mainly in Ethiopian community settings (inferred)	+++	+++	+++	+++	++	X	+
**Choi (1996) San Francisco, USA**	Single group session with a focus on sexual risk reduction for immigrant and ethnic minority gay and bisexual men	Content – exploration of positive ethnic and sexual identities<br><br>Gender and sexuality - of co-facilitator of group	+++	++	+++	+	++	X	X

First Author & Location	Intervention	Adaptation of intervention activities to increase cultural appropriateness	Mechanisms of adaptation 'authenticity'	Mechanisms of adaptation 'understanding'	Mechanisms of adaptation 'consonance'	Mechanisms of adaptation 'specificity'	Mechanisms of adaptation 'embeddedness'	Mechanisms of adaptation 'endorsement'	Mechanisms of adaptation 'framing'
**Conner (2004) California, USA**	Case studies of evaluation of two interventions<br><br>1. Tres Hombres sin Fronteras – a health promotion resource with a focus on sexual risk reduction for immigrant farmworkers	1. Language (Spanish)<br><br>Content – developed a low-literacy comic book (f*otonovela)*	1. +++	1. +++	1. +++	1. +++	1. ++	1. +++	1. +++
	2. SOLAAR - retreat for small groups with a focus on sexual risk reduction for immigrant gay men	2. Language (Spanish)<br><br>Bilingual Community Educators (BCEs) – deliver the retreat program<br><br>Gender and sexuality<br><br>Content- primary goal of retreat is 'clashing cultural norms' of immigrant cultures and urban American culture<br><br>Partnership/capacity building - between researchers and CBO (inferred)	2. +++	2. +++	2. +++	2. ++	2. +++	2. ++	2. +++

First Author & Location	Intervention	Adaptation of intervention activities to increase cultural appropriateness	Mechanisms of adaptation 'authenticity'	Mechanisms of adaptation 'understanding'	Mechanisms of adaptation 'consonance'	Mechanisms of adaptation 'specificity'	Mechanisms of adaptation 'embeddedness'	Mechanisms of adaptation 'endorsement'	Mechanisms of adaptation 'framing'
**Conner (2005)**<br><br>**California, USA**	SOLAAR retreat for small groups with a focus on sexual risk reduction for immigrant gay men<br><br>Social Marketing<br><br>Various other activities including outreach and health promotion resources	Language (Spanish)<br><br>Bilingual Community Educators (BCEs) – deliver the retreat program<br><br>Gender and sexuality<br><br>Content – primary goal of retreat program is 'clashing cultural norms' of immigrant cultures of participants and urban American culture<br><br>Settings-advertising retreats Latino gay and bisexual publications in California<br><br>Partnership/capacity building - between research organisation and CBOs	+++	+++	+++	+++	+++	++	+++

First Author & Location	Intervention	Adaptation of intervention activities to increase cultural appropriateness	Mechanisms of adaptation 'authenticity'	Mechanisms of adaptation 'understanding'	Mechanisms of adaptation 'consonance'	Mechanisms of adaptation 'specificity'	Mechanisms of adaptation 'embeddedness'	Mechanisms of adaptation 'endorsement'	Mechanisms of adaptation 'framing'
**Darrow (2004)**<br><br>**Florida, USA**	Reach 2010-<br>Four key activities to raise awareness (not reported in detail)-<br>outreach to residents and community leaders, mass media, Capacity building of CBOs using Abstinence, be Faithful, use Condoms (ABC) prevention messages with a focus on sexual risk reduction	Settings-targeting interventions to multiethnic Black and Hispanic neighbourhoods/communities informed by ethnographic assessments<br><br>Partnership/capacity building - between research organisation and three CBOs<br><br>Languages (Spanish and Creole) (inferred)<br><br>Content-focus groups & surveys with community groups to inform Community Action Plan	+	+++	+++	+	+++	+++	+++

First Author & Location	Intervention	Adaptation of intervention activities to increase cultural appropriateness	Mechanisms of adaptation ‘authenticity’	Mechanisms of adaptation ‘understanding’	Mechanisms of adaptation ‘consonance’	Mechanisms of adaptation ‘specificity’	Mechanisms of adaptation ‘embeddedness’	Mechanisms of adaptation ‘endorsement’	Mechanisms of adaptation ‘framing’
**Diaz (1998) San Francisco, USA**	Hermanos de Luna Y Sol, a range of activities to recruit gay and bisexual immigrant men – with an emphasis on low-income, monolingual, unacculturated men- into small group education and peer support activities with a focus on sexual risk reduction	Bilingual Community Educators (BCEs) – to facilitate group activities<br><br>Language (Spanish)<br><br>Gender and sexuality – of group facilitators<br><br>Content – incorporated dominant Latino cultural values on homosexuality in development of content<br><br>Settings-outreach and advertising to Latino gay and bisexual bars to recruit participants	+++	+++	+++	++	+++	++	++

First Author & Location	Intervention	Adaptation of intervention activities to increase cultural appropriateness	Mechanisms of adaptation 'authenticity'	Mechanisms of adaptation 'understanding'	Mechanisms of adaptation 'consonance'	Mechanisms of adaptation 'specificity'	Mechanisms of adaptation 'embeddedness'	Mechanisms of adaptation 'endorsement'	Mechanisms of adaptation 'framing'
**Flaskerud (1988) Los Angeles, USA**	12-minute slide show with supporting health promotion resources with a focus HIV knowledge, attitudes and sexual risk reduction for immigrants (mainly refugees)	Language (Vietnamese)<br><br>Bilingual Community Educators (BCEs) – Vietnamese educators<br><br>Gender<br><br>Content- developed taking into account Vietnamese cultural values , used Vietnamese imagery and characters<br><br>Settings-interventions delivered at a CBO targeting Indochinese refugees<br><br>Partnership/capacity building - between research organisation and refugee CBOs	+++	+++	+++	+++	+++	X	+

First Author & Location	Intervention	Adaptation of intervention activities to increase cultural appropriateness	Mechanisms of adaptation 'authenticity'	Mechanisms of adaptation 'understanding'	Mechanisms of adaptation 'consonance'	Mechanisms of adaptation 'specificity'	Mechanisms of adaptation 'embeddedness'	Mechanisms of adaptation 'endorsement'	Mechanisms of adaptation 'framing'
**Flaskerud (2000) Los Angeles, USA**	Various activities including - 1-1 counselling, group discussion, Spanish-language health promotion print and audio-visual resources - to reduce sexual and injecting drug use risks (not reported in detail) for low-income immigrant women	Language(Spanish)<br><br>Bilingual Community Educators (BCEs) – of health care workers who delivered the intervention in a women and children's health centre<br><br>Gender - of health care workers<br><br>Content- developed taking into account cultural values from focus groups and community consultations<br><br>Partnership/capacity building - between research organisation and women's health CBO	+++	++	+++	+++	+++	+++	+++

First Author & Location	Intervention	Adaptation of intervention activities to increase cultural appropriateness	Mechanisms of adaptation 'authenticity'	Mechanisms of adaptation 'understanding'	Mechanisms of adaptation 'consonance'	Mechanisms of adaptation 'specificity'	Mechanisms of adaptation 'embeddedness'	Mechanisms of adaptation 'endorsement'	Mechanisms of adaptation 'framing'
**Flaskerud (1997)**<br><br>**Los Angeles, USA**	Various activities including - 1-1 counselling, group discussion, Spanish-language health promotion print and audio-visual resources - to reduce sexual and injecting drug use risks (not reported in detail) for low-income immigrant women	Language(Spanish)<br><br>Bilingual Community Educators (BCEs) – of health care workers who delivered the intervention in a women and children's health centre<br><br>Gender<br><br>Content- developed taking into account cultural values	+++	++	+++	+++	+++	++	+++

First Author & Location	Intervention	Adaptation of intervention activities to increase cultural appropriateness	Mechanisms of adaptation 'authenticity'	Mechanisms of adaptation 'understanding'	Mechanisms of adaptation 'consonance'	Mechanisms of adaptation 'specificity'	Mechanisms of adaptation 'embeddedness'	Mechanisms of adaptation 'endorsement'	Mechanisms of adaptation 'framing'
**Gomez (1999) San Francisco USA**	Mujeres Unidas y Activas (MUA) - an intervention which aims to empower immigrant and refugee women and to assess effect on sexual risk reduction<br><br>MUA activities include support groups, workshops, HIV workshops and leadership training.<br><br>Participants at 6 months follow-up had attended a median of 9 MUA activities	Language(Spanish)<br><br>Bilingual Community Educators (BCEs) – of health care workers who delivered the intervention activities in a Latina CBO Gender - of health care workers<br><br>Content- developed taking into account cultural values (inferred)<br><br>Partnership & capacity building - between research organisation and Latina CBO	+++	+++	+++	+++	+++	+++	+++

First Author & Location	Intervention	Adaptation of intervention activities to increase cultural appropriateness	Mechanisms of adaptation 'authenticity'	Mechanisms of adaptation 'understanding'	Mechanisms of adaptation 'consonance'	Mechanisms of adaptation 'specificity'	Mechanisms of adaptation 'embeddedness'	Mechanisms of adaptation 'endorsement'	Mechanisms of adaptation 'framing'
**Haour-Knipe (1999) Switzerland**	Swiss Migrants Project – reporting on exploratory studies, process evaluation and outcome evaluations of three phases of the project<br><br>Various activities delivered – information dissemination to immigrant communities, HIV workshops, development of health promotion print and audio-visual resources, use of immigrant mass media, and some 1-1 support for PLWHA	Languages (Spanish, Portuguese and Turkish)<br><br>Bilingual Community Educators (BCEs) – Community Co-ordinators with teams of peer educators 'mediators'<br><br>Content - developed taking into account cultural values from exploratory needs analysis and community consultations<br><br>Settings-delivering interventions via existing structures in the three communities<br><br>Partnership/capacity building – of community 'mediators' and existing community structures	+++	+++	+++	+++	+++	+++	+++

First Author & Location	Intervention	Adaptation of intervention activities to increase cultural appropriateness	Mechanisms of adaptation ‘authenticity’	Mechanisms of adaptation ‘understanding’	Mechanisms of adaptation ‘consonance’	Mechanisms of adaptation ‘specificity’	Mechanisms of adaptation ‘embeddedness’	Mechanisms of adaptation ‘endorsement’	Mechanisms of adaptation ‘framing’
**Hlaing (2006) Florida, USA**	Evaluation of Reach 2010- Four key activities to raise awareness - outreach to residents and community leaders, mass media, capacity building of CBOs, using Abstinence, be Faithful, use Condoms prevention messages in a multiethnic community	Settings-targeting interventions to multiethnic neighbourhoods/communities<br><br>Partnership/capacity building - between research organisation and three CBOs<br><br>Language (Spanish and Creole)- in health promotion resources	+	++	X	+	++	X	+

First Author & Location	Intervention	Adaptation of intervention activities to increase cultural appropriateness	Mechanisms of adaptation 'authenticity'	Mechanisms of adaptation 'understanding'	Mechanisms of adaptation 'consonance'	Mechanisms of adaptation 'specificity'	Mechanisms of adaptation 'embeddedness'	Mechanisms of adaptation 'endorsement'	Mechanisms of adaptation 'framing'
**Hovey (2007)**<br><br>**Michigan, USA**	*Informate* theatrical presentation to young immigrants in 7 migrant farmworker camps	Language (Spanish)<br><br>Bilingual Community Educators (BCEs) – male and female young theatre players were from the target groups and called 'Teen Health Aides' (THAa)<br><br>Content- THAs developed the script taking into account cultural values<br><br>Settings-delivered theatrical presentation at 7 farmworker migrant camps to enhance accessibility	+++	+++	++	+++	+++	++	X

First Author & Location	Intervention	Adaptation of intervention activities to increase cultural appropriateness	Mechanisms of adaptation 'authenticity'	Mechanisms of adaptation 'understanding'	Mechanisms of adaptation 'consonance'	Mechanisms of adaptation 'specificity'	Mechanisms of adaptation 'embeddedness'	Mechanisms of adaptation 'endorsement'	Mechanisms of adaptation 'framing'
**Kim (2006) Chicago, USA**	Project SEPA, 6 group sessions with a focus on sexual risk reduction for low-income immigrant women<br><br>Study examined intervention participants compared to dropouts to assess predictors of participation	Language(Spanish)<br><br>Bilingual Community Educators (BCEs) – group facilitators<br><br>Gender - of facilitators<br><br>Content - of education sessions (inferred)<br><br>Settings - variety of methods to recruit participants including Spanish-language media	+++	+++	+++	++	++	X	X
**Kocken (2001) The Netherlands**	Single education session for male immigrants with a focus on HIV prevention and condom use	Language (Turkish and Arabic)<br><br>Bilingual Community Educators (BCEs) – 'lay heath advisors'<br><br>Gender - of 'lay heath advisors'<br><br>Settings- cafes and mosques to deliver education sessions	+++	+++	++	+++	+++	X	+++

First Author & Location	Intervention	Adaptation of intervention activities to increase cultural appropriateness	Mechanisms of adaptation 'authenticity'	Mechanisms of adaptation 'understanding'	Mechanisms of adaptation 'consonance'	Mechanisms of adaptation 'specificity'	Mechanisms of adaptation 'embeddedness'	Mechanisms of adaptation 'endorsement'	Mechanisms of adaptation 'framing'
**Loue (1996)**<br><br>**San Diego, USA**	Establishment of a prevention program and organization to address HIV/AIDS among Asian and Pacific Islanders-Project HAPI	Content of HIV prevention program developed taking into account cultural values from qualitative and quantitative needs analysis<br><br>Settings-use of 'gatekeepers' to access Asian and Pacific Islander communities<br><br>Partnership/capacity building – of a range community agencies in working groups, program planning and 'political' meetings	+++	+++	+++	+	+++	+++	+

First Author & Location	Intervention	Adaptation of intervention activities to increase cultural appropriateness	Mechanisms of adaptation 'authenticity'	Mechanisms of adaptation 'understanding'	Mechanisms of adaptation 'consonance'	Mechanisms of adaptation 'specificity'	Mechanisms of adaptation 'embeddedness'	Mechanisms of adaptation 'endorsement'	Mechanisms of adaptation 'framing'
**Marahaj (1999)**<br><br>**UK**	Assessment of a range of HIV prevention interventions with refugees and asylum seekers. Four summary case studies of interventions at group and community levels:<br>1. African Communities HIV Project (ACHP)<br><br>2. African Health Football Project	• 1. African Communities HIV Project<br><br>Partnership/capacity building – building alliances to create co-ordinated HIV prevention activities with African communities<br><br>• 2. African Health Football Project<br><br>Settings -integrated HIV health promotion into an African soccer tournament<br><br>Bilingual Community Educators (BCEs) – volunteer peer educators from 'Black' HIV services were used to distribute HIV information and staff the intervention<br><br>Partnership/capacity building –tournament players and managers	1. +<br><br>2. +++	1. +<br><br>2. +	1. +<br><br>2. +++	1. ++<br><br>2. +++	1. +++<br><br>2. +++	1. +++<br><br>2. +++	1. X<br><br>2. ++

		and 'Black' volunteers planned the intervention							
	3. Ugandan AIDS Action Play	• 3. Ugandan AIDS Action Play<br><br>Bilingual Community Educators (BCEs) – commissioned a Ugandan theatre group to develop a HIV/AIDS play<br><br>Content – commissioned a Ugandan theatre group to develop a play around HIV/AIDS<br><br>Settings- used a culturally appropriate and popular medium of communication to discuss HIV/AIDS and a variety of community settings to deliver education sessions and perform the play	3. +++	3. +	3. +++	3. +++	3. +++	3. +++	3. +++

	4. Refugee Council's HIV and AIDS Development Work	• 4. Refugee Council's HIV and AIDS Development Work<br><br>Language – HIV prevention resources in 8 languages<br><br>Content – of training for HIV and refugee service providers reflected HIV <u>and</u> refugee issues<br><br>Partnership/capacity building - support to refugee service providers for HIV work, linking refugee and HIV service providers	4. +	4. +	4. +	4. X	4. +	4. X	4. X

First Author & Location	Intervention	Adaptation of intervention activities to increase cultural appropriateness	Mechanisms of adaptation 'authenticity'	Mechanisms of adaptation 'understanding'	Mechanisms of adaptation 'consonance'	Mechanisms of adaptation 'specificity'	Mechanisms of adaptation 'embeddedness'	Mechanisms of adaptation 'endorsement'	Mechanisms of adaptation 'framing'
**Martijn (2004)**<br><br>**Rotterdam, The Netherlands**	Two studies of a single education session for male immigrants focus on HIV prevention and condom use<br><br>Study 1 – delivered by 'lay health advisors'<br><br>Study 2- delivered by 'lay health advisors' and 'professional health advisors' (with an interpreter)	Language (Turkish and Arabic)<br><br>Bilingual Community Educators (BCEs) – 'lay health advisors' in both studies. Interpreter was used in Study 2 with 'professional health advisor'<br><br>Gender - 'lay health advisors' & 'professional health advisors'<br><br>Settings-used two settings. Study 1 at a Newcomer Integration Project and Study 2 at a refugee reception centre	1. +++<br><br>2. ++	1. +++<br><br>2. ++	1. ++<br><br>2. +	1. ++<br><br>2. ++	1. +<br><br>2. +	1. X<br><br>2. X	1. X<br><br>2. X

First Author & Location	Intervention	Adaptation of intervention activities to increase cultural appropriateness	Mechanisms of adaptation 'authenticity'	Mechanisms of adaptation 'understanding'	Mechanisms of adaptation 'consonance'	Mechanisms of adaptation 'specificity'	Mechanisms of adaptation 'embeddedness'	Mechanisms of adaptation 'endorsement'	Mechanisms of adaptation 'framing'
**Martin (2005)**<br><br>**Chicago, USA**	Centro San Bonifacio HIV Prevention Program Education sessions carried out in the community for male and female immigrants with a focus on HIV knowledge and awareness	Language (Spanish)<br><br>Bilingual Community Educators (BCEs) – 'promotoras' were used to recruit and deliver education sessions<br>Settings-used a variety of settings to deliver education session including people's homes, schools, Church groups	+++	+++	+	++	+++	X	+
**Maxwell (2002)**<br><br>**Los Angeles, USA**	Single education session to young immigrant attendees at a HIV clinic with a focus on condom use and sexual negotiation	Language (Spanish)	+	+	X	X	X	X	X

First Author & Location	Intervention	Adaptation of intervention activities to increase cultural appropriateness	Mechanisms of adaptation 'authenticity'	Mechanisms of adaptation 'understanding'	Mechanisms of adaptation 'consonance'	Mechanisms of adaptation 'specificity'	Mechanisms of adaptation 'embeddedness'	Mechanisms of adaptation 'endorsement'	Mechanisms of adaptation 'framing'
**Mc Mahon (2005)**<br><br>**Sydney & Melbourne, Australia**	Pilot ethnic media advertising and publicity campaign targeting immigrants to promote HIV testing and access to treatment and care by PLWHA at public sexual health clinics	Language(multiple)<br><br>Bilingual Community Educators (BCEs) – as media spokespeople on radio (inferred)<br><br>Content – campaign themes generated from a needs assessment, refined with focus groups<br><br>Settings-advertising and publicity used media accessed by immigrants	+++	+++	++	+	+++	+	X
**Mishra (1996)**<br><br>**California, USA**	Tres Hombres sin Fronteras – two health promotion resources with a focus on sexual risk reduction for immigrant male farmworkers	Language (Spanish)<br><br>Content – developed a low-literacy comic book (f*otonovela)* and low-health literacy radio segments *(radionovela)*<br><br>Settings-outreach to farm camps to distribute resources	++	+++	++	+++	+++	+	+

First Author & Location	Intervention	Adaptation of intervention activities to increase cultural appropriateness	Mechanisms of adaptation 'authenticity'	Mechanisms of adaptation 'understanding'	Mechanisms of adaptation 'consonance'	Mechanisms of adaptation 'specificity'	Mechanisms of adaptation 'embeddedness'	Mechanisms of adaptation 'endorsement'	Mechanisms of adaptation 'framing'
**Nakyonyi (1993)**<br><br>**Toronto, Canada**	Africans United to Control AIDS (AUCA)<br><br>Various activities described including information dissemination to African immigrants, HIV workshops, outreach to African community organisations, use of immigrant mass media	Languages (multiple)<br><br>Bilingual Community Educators (BCEs) – African volunteers recruited and trained as community educators/outreach workers<br><br>Content – activities are developed in partnership with community groups (inferred)<br><br>Settings-use of community events and venues and use of media accessed by African immigrants	+++	++	++	++	+++	+++	++

First Author & Location	Intervention	Adaptation of intervention activities to increase cultural appropriateness	Mechanisms of adaptation 'authenticity'	Mechanisms of adaptation 'understanding'	Mechanisms of adaptation 'consonance'	Mechanisms of adaptation 'specificity'	Mechanisms of adaptation 'embeddedness'	Mechanisms of adaptation 'endorsement'	Mechanisms of adaptation 'framing'
**Organista (2006) Mexico border area, USA**	Two pilot education sessions for Latino Migrant Day with a focus on sexual risk reduction, condom use and reduced alcohol and substance use	Language (inferred) (Spanish)<br><br>Content- of education sessions developed from formative quant and qual research, use of *Lotteria* cards, *fotonovelas*, *c*omic-style books, and *radionovelas*	++	++	+++	++	++	+	++
**Peragallo (2005)<br><br>Chicago, USA**	Project SEPA, 6 group sessions with a focus on sexual risk reduction for low-income immigrant women	Language (Spanish)<br><br>Bilingual Community Educators (BCEs) – group facilitators<br><br>Gender - of group facilitators<br><br>Content- of education sessions (inferred)<br><br>Settings- a variety of methods to recruit participants including Spanish-language media	+++	+++	++	+++	+++	X	+

**First Author & Location**	**Intervention**	**Adaptation of intervention activities to increase cultural appropriateness**	**Mechanisms of adaptation 'authenticity'**	**Mechanisms of adaptation 'understanding'**	**Mechanisms of adaptation 'consonance'**	**Mechanisms of adaptation 'specificity'**	**Mechanisms of adaptation 'embeddedness'**	**Mechanisms of adaptation 'endorsement'**	**Mechanisms of adaptation 'framing'**
**Raj (2001)**<br><br>**Boston, USA**	Comparison of two interventions to reach low-income immigrant women:<br>1. HIV-specific with participatory education;<br>2. women's health specific and didactic<br><br>Each intervention 12 group sessions & 16 hours of content- with a focus on sexual risk reduction	Language (Spanish)<br><br>Bilingual Community Educators (BCEs) – to facilitate groups<br><br>Content- of education sessions (inferred)<br><br>Gender - of group facilitator	1. +++<br><br>2. +++	1. +++<br><br>2. +++	1. +++<br><br>2. +++	1. ++<br><br>2. ++	1. ++<br><br>2. +	1. X<br><br>2. X	1. X<br><br>2. X

First Author & Location	Intervention	Adaptation of intervention activities to increase cultural appropriateness	Mechanisms of adaptation 'authenticity'	Mechanisms of adaptation 'understanding'	Mechanisms of adaptation 'consonance'	Mechanisms of adaptation 'specificity'	Mechanisms of adaptation 'embeddedness'	Mechanisms of adaptation 'endorsement'	Mechanisms of adaptation 'framing'
**Shtarkshall (2000)**<br><br>**Israel**	Overview of model for the development of HIV/AIDS programs for immigrants using two programs for immigrants as case studies:<br>1. Immigrant youth from the former Soviet Union and Ethiopia<br><br>2. Ethiopian immigrants	Model proposes an iterative approach to program development including considering: 'old country' and 'new country' values; the cultural transitions inherent in immigration; employing immigrants as 'cultural insighters'; harnessing leadership from the immigrant community; developing culturally specific messages; workforce development of host country health care workers	1. +<br><br>2. +++	1. X<br><br>2. +++	1. ++<br><br>2. +++	1. +<br><br>2. +++	1. X<br><br>2. X	1. X<br><br>2. X	1. ++<br><br>2. ++

First Author & Location	Intervention	Adaptation of intervention activities to increase cultural appropriateness	Mechanisms of adaptation 'authenticity'	Mechanisms of adaptation 'understanding'	Mechanisms of adaptation 'consonance'	Mechanisms of adaptation 'specificity'	Mechanisms of adaptation 'embeddedness'	Mechanisms of adaptation 'endorsement'	Mechanisms of adaptation 'framing'
**Somerville (2006)**<br><br>**California and Texas, USA**	Training popular opinion leaders to carry out HIV outreach among migrant men who have sex with men	Popular Opinion Leader intervention adapted after needs analysis<br><br>Language (Spanish) – of training and evaluation material<br><br>Bilingual Community Educators (BCEs) – YPLs outreach<br><br>Gender - of YPLs<br><br>Content – of YPL training adapted to:<br>• build on Latino cultural understanding of *promotoras*<br>• address higher HIV info needs of Latino MSM<br>• place of sexual orientation in YPLs lives<br>• referral skills<br><br>Settings - in locales known to have high nos. of Latino farm workers	+++	+++	+++	++	+++	++	++

First Author & Location	Intervention	Adaptation of intervention activities to increase cultural appropriateness	Mechanisms of adaptation 'authenticity'	Mechanisms of adaptation 'understanding'	Mechanisms of adaptation 'consonance'	Mechanisms of adaptation 'specificity'	Mechanisms of adaptation 'embeddedness'	Mechanisms of adaptation 'endorsement'	Mechanisms of adaptation 'framing'
**Soskolne (2002)**<br><br>**Israel**	Overview of model for the development of HIV/AIDS programs for immigrants using several programs examples as case studies:	Model proposes an iterative approach to program development, involving immigrants as 'cultural insighters' for programs specific to immigrant youth and Ethiopian immigrants.							
	1. Immigrant youth from the former Soviet Union and Ethiopia		1. ++	1. +	1. ++	1. +	1. X	1. X	1. X
	2. Ethiopian immigrants		2. +	2. X	2. ++	2. ++	2. X	2. X	2. X

First Author & Location	Intervention	Adaptation of intervention activities to increase cultural appropriateness	Mechanisms of adaptation 'authenticity'	Mechanisms of adaptation 'understanding'	Mechanisms of adaptation 'consonance'	Mechanisms of adaptation 'specificity'	Mechanisms of adaptation 'embeddedness'	Mechanisms of adaptation 'endorsement'	Mechanisms of adaptation 'framing'
**Worth (2003)**<br><br>**New Zealand**	National HIV/AIDS Refugee Health Education Program which comprised training BCEs , developing community-specific action plans, workshops, resource development, support for	Language – various African languages used in workshops<br><br>Bilingual Community Educators (BCEs) – from a range of Sub-Saharan African backgrounds<br><br>Content- of education workshops and health promotion resources- used ABC<br><br>Settings-used a variety of community settings for workshops and developed community HIV/AIDS health promotion action plans	+++	+++	++	+++	+++	++	+++
