# Supplementary material for: HIV among immigrants living in high-income countries: a realist review of evidence to guide targeted approaches to behavioural HIV prevention
Source: Syst Rev. 2012 Nov 20;1:56. doi: 10.1186/2046-4053-1-56 (PMC3534573; doi:10.1186/2046-4053-1-56)
Supplement: Additional file 7 — Annotation of views studies: Summary against adaptive mechanisms. [file 2046-4053-1-56-S7.pdf]

Annotation of views studies: Summary against adaptive mechanisms

Key: sound (+++), moderate (++), partial (+) or no evidence (X)

First Author & Location	Study aims	Relevance and cultural appropriateness of 'authenticity'	Relevance and cultural appropriateness of 'understanding'	Relevance and cultural appropriateness of 'consonance'	Relevance and cultural appropriateness of 'specificity'	Relevance and cultural appropriateness of 'embeddedness'	Relevance and cultural appropriateness of 'endorsement'	Relevance and cultural appropriateness of 'framing'
**Anderson (2004) London, UK**	Explore the lived experiences of immigrant women with HIV	X	X	+++	+	+	++	+++
**Apostolopoulos (2006) Arizona & South Carolina, USA**	Examine intertwined individual and environmental factors in relation to HIV/STI risks among male –gay and straight-immigrant farm workers	X	+	++	+	++	X	+++
**Ayala (2001) Miami, New York City, Los Angeles, USA**	Analyze the relationship between race, class, and unprotected sex among gay men	+	+	++	+	+++	+	+++

First Author & Location	Study aims	Relevance and cultural appropriateness of 'authenticity'	Relevance and cultural appropriateness of 'understanding'	Relevance and cultural appropriateness of 'consonance'	Relevance and cultural appropriateness of 'specificity'	Relevance and cultural appropriateness of 'embeddedness'	Relevance and cultural appropriateness of 'endorsement'	Relevance and cultural appropriateness of 'framing'
**Bhattacharya (2004) New York City, USA**	Explore social capital resources and influences on HIV risk behaviours among immigrant heterosexual men	X	X	++	++	++	++	++
**Castro-Vazquez (2006) Japan**	Explore the relationship between HIV/AIDS and 'community' support for immigrants with HIV	X	++	+++	+++	+	+	+++
**Chin (1999) Los Angeles, USA**	Explore how immigrant women assess HIV risk in social interactions	X	X	++	X	X	X	+
**Chin (2007) New York City, USA**	Explore the experiences and needs of immigrants living with HIV/AIDS	++	+++	++	++	+++	+++	+++

First Author & Location	Study aims	Relevance and cultural appropriateness of 'authenticity'	Relevance and cultural appropriateness of 'understanding'	Relevance and cultural appropriateness of 'consonance'	Relevance and cultural appropriateness of 'specificity'	Relevance and cultural appropriateness of 'embeddedness'	Relevance and cultural appropriateness of 'endorsement'	Relevance and cultural appropriateness of 'framing'
**Chinouya (2006) Milton Keynes, UK**	Explore the role of faith, family life, service utilization and coping among immigrants living with HIV	+	X	+++	X	+++	+++	+++
**Dawson (2001) Melbourne, Australia**	Explore immigrant women's gender and sexual identities in relation to HIV and sexual health	X	X	+++	+++	+	++	+++
**Dawson (2003) Melbourne, Australia & Santiago, Chile**	Explore past/ present social changes on life experiences related to HIV/AIDS among immigrant women and women in the 'home' country	X	+	++	+	X	X	++

First Author & Location	Study aims	Relevance and cultural appropriateness of 'authenticity'	Relevance and cultural appropriateness of 'understanding'	Relevance and cultural appropriateness of 'consonance'	Relevance and cultural appropriateness of 'specificity'	Relevance and cultural appropriateness of 'embeddedness'	Relevance and cultural appropriateness of 'endorsement'	Relevance and cultural appropriateness of 'framing'
**Diaz (1999) Miami, New York City, Los Angeles, USA**	Explore high rates of unprotected anal intercourse among immigrant gay and bisexual men	X	+	+++	++	X	X	++
**Diaz (2000) Miami, New York City, Los Angeles, USA**	Explore impact of poverty, racism and homophobia on sexual risks, substance use & psych. distress among immigrant gay and bisexual men	X	X	+++	+	X	X	+++
**Dodds (2006) UK**	Explore social discourses on HIV-related stigma among people incl. HIV-positive immigrants	++	X	+++	++	+++	+	+++

First Author & Location	Study aims	Relevance and cultural appropriateness of 'authenticity'	Relevance and cultural appropriateness of 'understanding'	Relevance and cultural appropriateness of 'consonance'	Relevance and cultural appropriateness of 'specificity'	Relevance and cultural appropriateness of 'embeddedness'	Relevance and cultural appropriateness of 'endorsement'	Relevance and cultural appropriateness of 'framing'
**Dodds (2004) UK**	Explore social discourses around HIV-related stigma and discrimination among a range of people including immigrants living with HIV	X	X	++	+	+++	+	+++
**Doyal (2003) London, UK**	Explore the lived experiences of immigrant women with HIV	X	X	+++	++	++	++	+++
**Doyal (2005) London, UK**	Explore the lived experiences of immigrant women with HIV	X	X	+++	X	++	X	+++

First Author & Location	Study Aims	Relevance and cultural appropriateness of 'authenticity'	Relevance and cultural appropriateness of 'understanding'	Relevance and cultural appropriateness of 'consonance'	Relevance and cultural appropriateness of 'specificity'	Relevance and cultural appropriateness of 'embeddedness'	Relevance and cultural appropriateness of 'endorsement'	Relevance and cultural appropriateness of 'framing'
**Doyal (2006) London, UK**	Explore the lived experiences of immigrant women with HIV	X	X	++	X	+	X	++
**Flaskerud (1991) Los Angeles, USA**	Explore HIV/AIDS health beliefs, and their relationship to 'traditional' beliefs, among low-income immigrant women	X	++	++	+++	X	X	++
**Flaskerud (1996) Los Angeles, USA**	Explore sexual practices, attitudes and knowledge related to HIV transmission among low-income immigrant women	X	+	+++	X	X	X	+++

First Author & Location	Study aims	Relevance and cultural appropriateness of 'authenticity'	Relevance and cultural appropriateness of 'understanding'	Relevance and cultural appropriateness of 'consonance'	Relevance and cultural appropriateness of 'specificity'	Relevance and cultural appropriateness of 'embeddedness'	Relevance and cultural appropriateness of 'endorsement'	Relevance and cultural appropriateness of 'framing'
**Gifford (1998) Melbourne, Australia**	Explore ways in which immigrant women understand risks to sexual health including HIV	X	++	+++	+++	+++	+	+++
**Hirsch (2002) Atlanta, USA and Mexico**	Explore social constructions of HIV risks among immigrant women and women in the 'home' country	X	+++	++	++	+	X	+++
**Jemmott (1999) New York City, USA**	Explore perceptions of HIV risks and culturally appropriate consideration s for HIV prevention among immigrant women	X	+++	++	+++	++	+	+++

First Author & Location	Study aims	Relevance and cultural appropriateness of 'authenticity'	Relevance and cultural appropriateness of 'understanding'	Relevance and cultural appropriateness of 'consonance'	Relevance and cultural appropriateness of 'specificity'	Relevance and cultural appropriateness of 'embeddedness'	Relevance and cultural appropriateness of 'endorsement'	Relevance and cultural appropriateness of 'framing'
**Kang (2003) New York City, USA**	Explore cultural attitudes, and HIV-related behaviours and perceptions among undocumented immigrants living with HIV	+++	+++	+++	++	+	X	+++
**Korner (2007a) Sydney, Australia**	Explore the lived experience of immigrants living with HIV/AIDS – residency issues	+++	+++	++	++	+	X	+++
**Korner (2007b) Sydney, Australia**	Explore the lived experience of immigrants living with HIV/AIDS – late HIV diagnosis	X	+	+++	+	+	X	+++

First Author & Location	Study aims	Relevance and cultural appropriateness of 'authenticity'	Relevance and cultural appropriateness of 'understanding'	Relevance and cultural appropriateness of 'consonance'	Relevance and cultural appropriateness of 'specificity'	Relevance and cultural appropriateness of 'embeddedness'	Relevance and cultural appropriateness of 'endorsement'	Relevance and cultural appropriateness of 'framing'
**Korner (2007c) Sydney, Australia**	Explore the lived experience of immigrants living with HIV/AIDS – disclosure issues	X	+++	+++	+	+	X	+++
**Korner (2005) Sydney, Australia**	Explore the lived experience of immigrants living with HIV/AIDS	+++	+++	+++	X	X	X	+++
**McQuiston (1998) North Carolina, USA**	Explore the preventive practices for HIV and STIs of newly arrived immigrants	X	+++	+++	+++	++	X	+++
**McQuiston (2000) North Carolina, USA**	Explore the preventive practices for HIV of newly arrived immigrants	X	+	++	+++	+	X	+++

First Author & Location	Study Aims	Relevance and cultural appropriateness of 'authenticity'	Relevance and cultural appropriateness of 'understanding'	Relevance and cultural appropriateness of 'consonance'	Relevance and cultural appropriateness of 'specificity'	Relevance and cultural appropriateness of 'embeddedness'	Relevance and cultural appropriateness of 'endorsement'	Relevance and cultural appropriateness of 'framing'
**Moreno (2007) New York City, USA**	Explore the contextual risks of HIV-positive status and domestic violence against immigrant women	+	+++	+++	+	+	X	+++
**Nemoto (2005) San Francisco, USA**	Explore the cognitive, cultural and contextual factors that influence HIV-related risk behaviours among female immigrants who engage in sex work at massage parlours and owners/ Managers	+	+++	+++	X	++	X	+++

First Author & Location	Study aims	Relevance and cultural appropriateness of 'authenticity'	Relevance and cultural appropriateness of 'understanding'	Relevance and cultural appropriateness of 'consonance'	Relevance and cultural appropriateness of 'specificity'	Relevance and cultural appropriateness of 'embeddedness'	Relevance and cultural appropriateness of 'endorsement'	Relevance and cultural appropriateness of 'framing'
**Poon (2002) Toronto, Canada**	Explore cultural and social barriers influencing HIV-related risks among gay, lesbian, and bisexual young immigrants	++	X	++	X	++	X	+++
**Rhodes (2007) North Carolina, USA**	Explore cultural and social determinants of HIV risk and identify potential intervention approaches among immigrant men	+	++	++	X	++	X	+++
**Shedlin (2005) New York State, USA**	Explore social and behavioural factors influencing HIV risk among newly arrived immigrants	+	++	+++	X	+++	X	+++

First Author & Location	Study aims	Relevance and cultural appropriateness of 'authenticity'	Relevance and cultural appropriateness of 'understanding'	Relevance and cultural appropriateness of 'consonance'	Relevance and cultural appropriateness of 'specificity'	Relevance and cultural appropriateness of 'embeddedness'	Relevance and cultural appropriateness of 'endorsement'	Relevance and cultural appropriateness of 'framing'
**Shedlin (2002) New York City, USA**	Explore predominant cultural influences social regarding sex work, drug use and HIV/AIDS among immigrants with very high risks for HIV/AIDS	X	+	+++	+++	+++	++	+++
**Shedlin (2004) New York City, USA**	Explore perceptions, beliefs experiences and knowledge of care issues for immigrants most of whom were living with HIV	X	+++	+++	++	+	X	+++

First Author & Location	Study aims	Relevance and cultural appropriateness of 'authenticity'	Relevance and cultural appropriateness of 'understanding'	Relevance and cultural appropriateness of 'consonance'	Relevance and cultural appropriateness of 'specificity'	Relevance and cultural appropriateness of 'embeddedness'	Relevance and cultural appropriateness of 'endorsement'	Relevance and cultural appropriateness of 'framing'
**Steel (2003) Sweden**	Explore the impact of pre-migration trauma on HIV risk behaviour among refugees post-settlement (relevant to HIV prevention)	X	X	+	X	X	X	++
**Wilson (2004) New York City, USA**	Explore experiences of, and responses to, social discrimination among gay immigrants	+	X	+	+	X	X	+++
**Yoshikawa (2003) Northeastern city, USA**	Explore the experiences of immigrant educators to implement culturally anchored HIV prevention among immigrants	++	++	+++	++	+++	++	+++

First Author & Location	Study aims	Relevance and cultural appropriateness of 'authenticity'	Relevance and cultural appropriateness of 'understanding'	Relevance and cultural appropriateness of 'consonance'	Relevance and cultural appropriateness of 'specificity'	Relevance and cultural appropriateness of 'embeddedness'	Relevance and cultural appropriateness of 'endorsement'	Relevance and cultural appropriateness of 'framing'
**Zuniga (2006), San Diego/Tijuana, USA/Mexico**	Explore barriers and facilitators to service utilisation by HIV-positive immigrants	X	+++	+	+	+	X	++
