# Supplementary material for: HIV among immigrants living in high-income countries: a realist review of evidence to guide targeted approaches to behavioural HIV prevention
Source: Syst Rev. 2012 Nov 20;1:56. doi: 10.1186/2046-4053-1-56 (PMC3534573; doi:10.1186/2046-4053-1-56)
Supplement: Additional file 9 — PRISMA flow diagram. [file 2046-4053-1-56-S9.pdf]

PRISMA 2009 Flow Diagram

Intervention studies

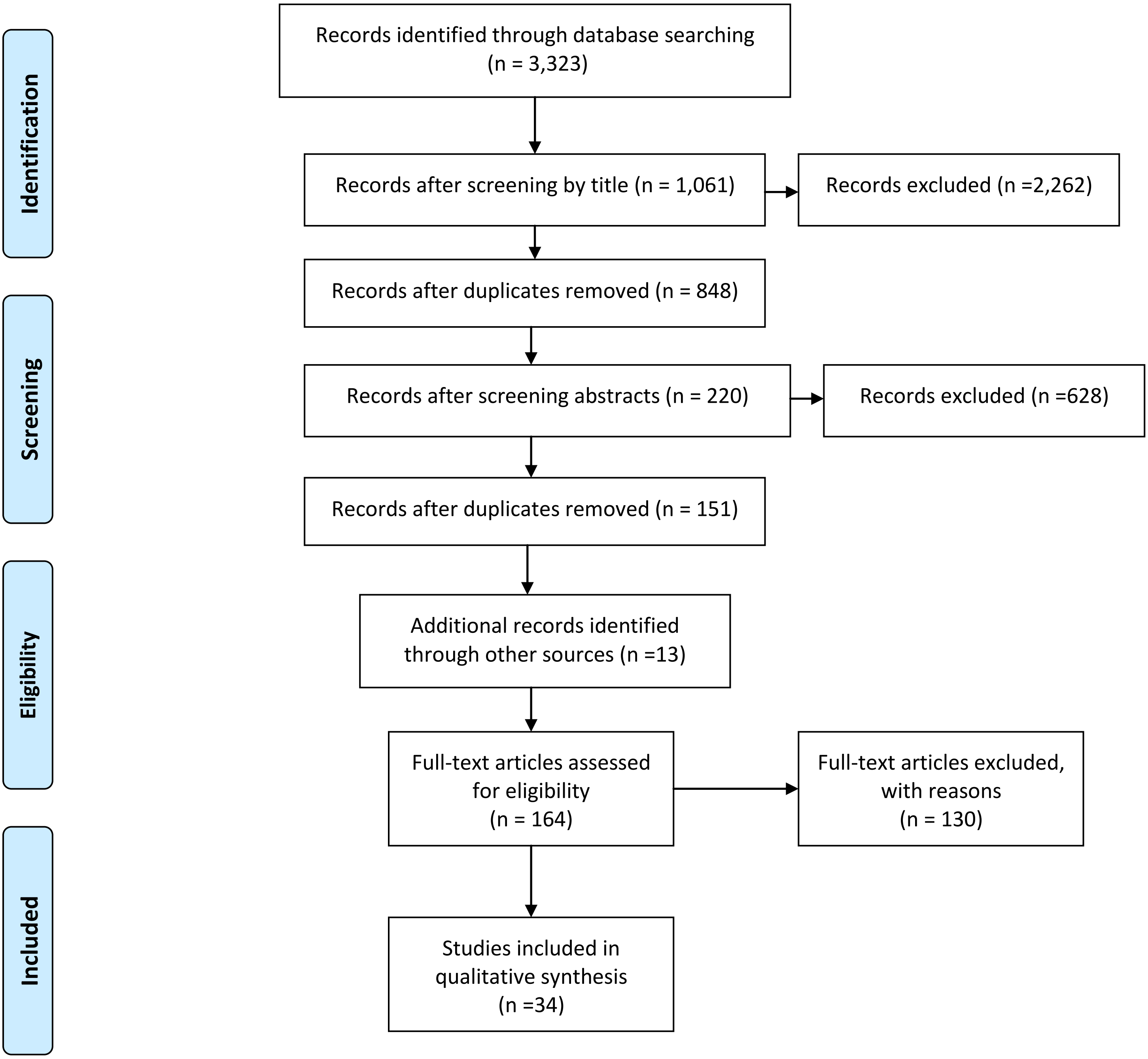

Adapted from Moher, D., et al., *Preferred reporting items for systematic reviews and meta-analyses: The PRISMA Statement.* PLoS Medicine, 2009. **6**(7).

Views studies

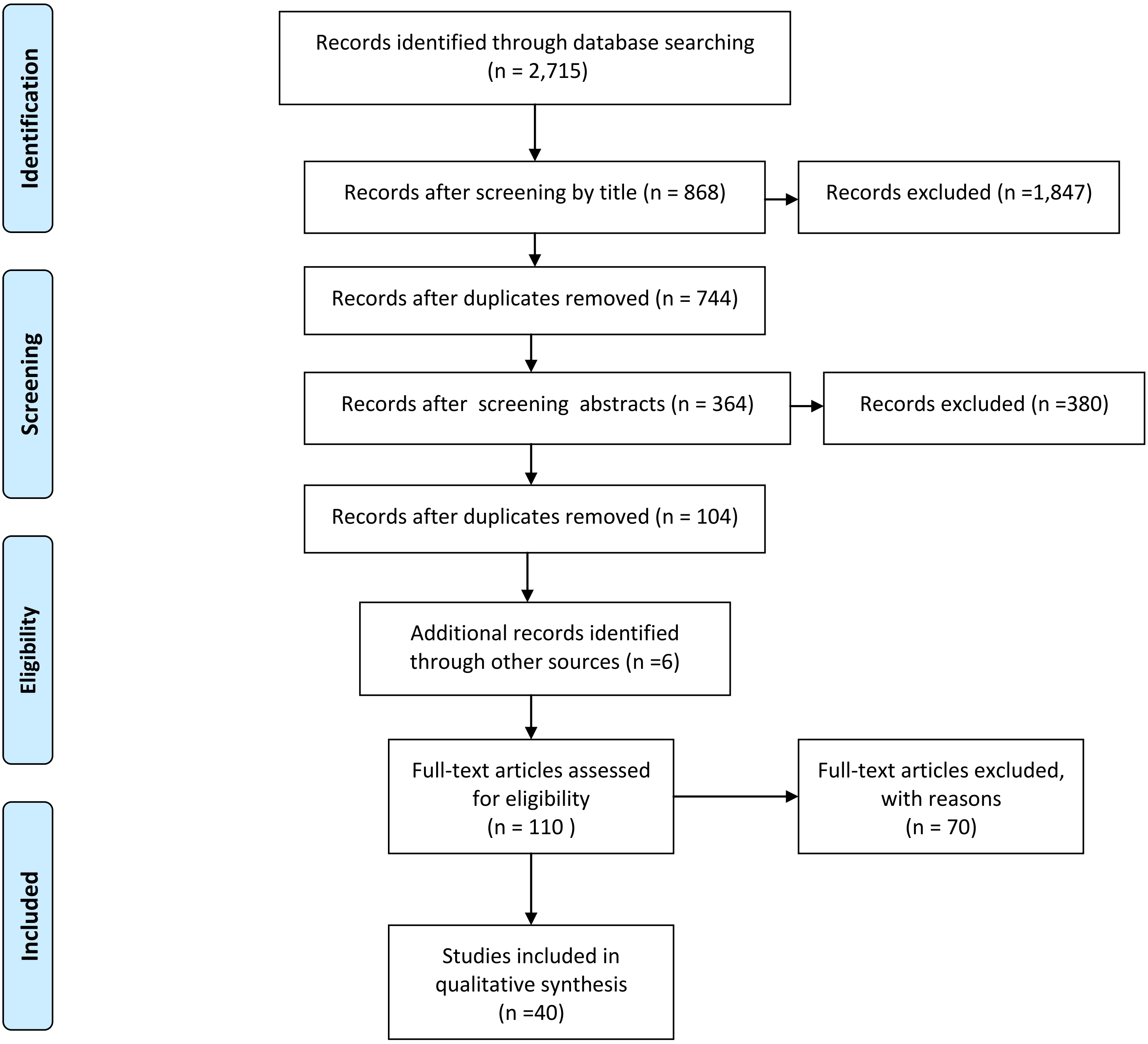

Adapted from Moher, D., et al., *Preferred reporting items for systematic reviews and meta-analyses: The PRISMA Statement.* PLoS Medicine, 2009. **6**(7).
