# Supplementary material for: HIV among immigrants living in high-income countries: a realist review of evidence to guide targeted approaches to behavioural HIV prevention
Source: Syst Rev. 2012 Nov 20;1:56. doi: 10.1186/2046-4053-1-56 (PMC3534573; doi:10.1186/2046-4053-1-56)
Supplement: Additional file 10 — Supplementary results: evidence synthesis and discussion. [file 2046-4053-1-56-S10.pdf]

Supplementary results: evidence synthesis and discussion

'Authenticity'

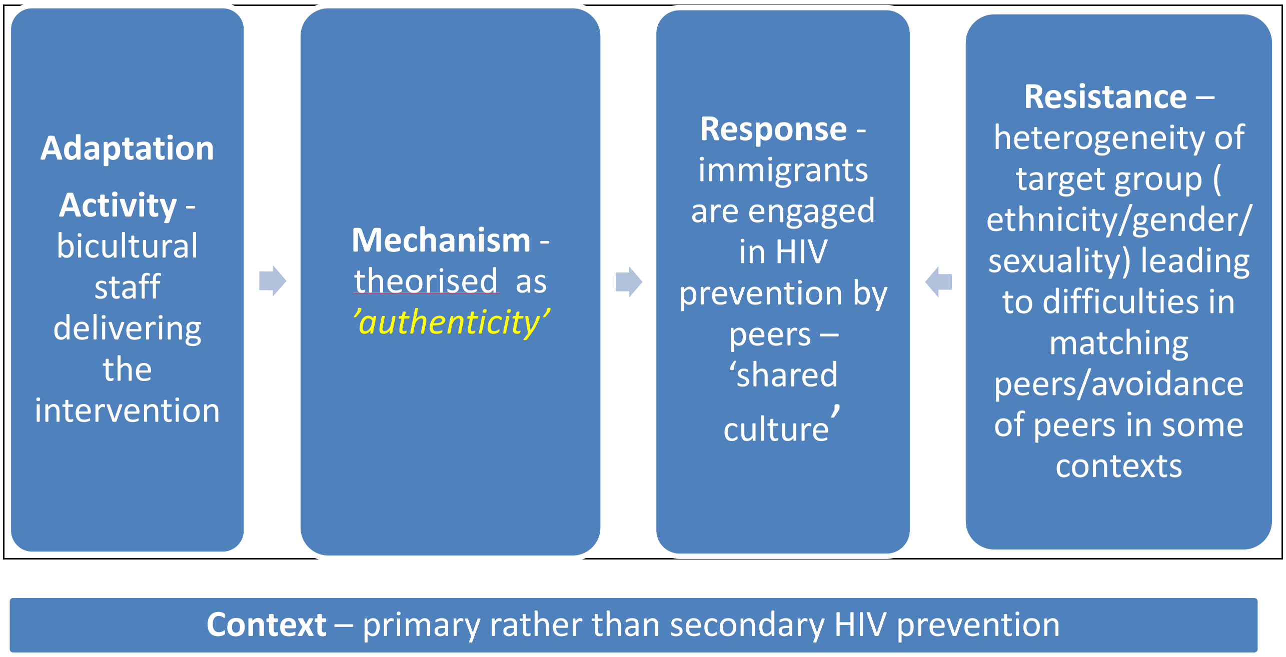

The evidence around '*authenticity*' mechanisms

Activity

The evidence from the intervention studies strongly supported this mechanism. Thirty-seven interventions reported sound or moderate evidence indicating a strong role of this theorised mechanism in culturally appropriate HIV prevention with immigrants. This mechanism was found to be operationalised mainly through the use of peers, usually bicultural community educators in the design, delivery and dissemination of the intervention [1-31]. Several interventions refined this mechanism through matching staff in terms of the gender and/or sexuality [1-7, 10, 14, 19, 21, 22, 24, 25, 27]. Indeed in some contexts using bicultural women as *promotoras* (outreach health educators) is a culturally recognised way in Latino communities to disseminate health information through families and social networks [7, 25].

In contrast, sound or moderate evidence to support this mechanism was reported in only seven views studies indicating that this mechanism was not widely supported by immigrants themselves to enhance cultural appropriate HIV prevention [32-37]. There was partial evidence reported in seven studies for refining the match of bicultural peers in terms of gender or sexuality [38-44]. No intervention studies or view studies reported evidence to support engaging HIV-positive people as peer educators. The sole exception came from a sample of HIV-positive African-born heterosexual men and women in the UK who indicated a desire for peer support from other HIV-positive Africans [37]. Twenty-six of the views studies did not report any evidence to support the '*authenticity*' mechanism in HIV prevention.

Response and resistance

The theorised response of immigrants to this mechanism is that they perceive the intervention is 'for them' as the staff delivering it are peers and, therefore, 'like them' in terms of a 'shared culture'. In general intervention studies did not report on the feedback of program participants on this specific mechanism but there was positive feedback reported in two studies of an intervention with Latina immigrant women in the USA, [7, 25] in the evaluation of a national program to reach immigrants in Switzerland, [20] and in an intervention with Turkish and Moroccan immigrants in the Netherlands [22]. There were reports of general satisfaction or improved recruitment to intervention activities among participants in four studies that employed this mechanism [2, 5, 12, 18] though this was not attributed specifically to the use of bicultural peers. Sound evidence for the '*authenticity*' mechanism was found in two views studies of HIV-positive people which reported on the merits of staff and peers from the same culture/ethnicity [34, 35].

There were few explicit reported resistances from intervention participants to this mechanism. Some resistance could be inferred from the heterogeneity – in terms of ethnicity and countries of origin – which was reported as a barrier to staffing interventions with African immigrants in Canada

and in the UK [12, 18]. In terms of gender, one intervention using this mechanism was unable to recruit female Iraqi participants [10] and one group intervention reported resistance to female peer *promotoras* as it was perceived that they would not be effective in disseminating prevention messages to either young or older men [7, 25]. This perception was also supported in a views study [45] where Latino male farm workers in the USA indicated the importance of using male peers to overcome the inherent sensitivities in HIV prevention [40]. Two interventions employing this mechanism reported a perception of limited appeal of the interventions to HIV-positive people [3, 17] but the evidence is inconclusive as to whether the perceived lack of reach of programs to HIV-positive people was related to this mechanism or other program issues. In view studies, HIV-positive immigrants overwhelmingly expressed a strong desire for anonymity, secrecy and confidentiality around their HIV status when accessing health services and especially avoiding people from the same ethnicity [46]. This suggests a resistance to peers (and this mechanism) from HIV-positive immigrants who also generally rated the staff of HIV services (who were never reported to be from the same ethnicity) and the care they received from these health services highly [47-52]. There was also some evidence of resistance to this mechanism from Latino gay men in the USA [38] who reported that peers might be a source of prejudice against Latino HIV-positive or Latino gay men.

Context

The evidence to support this adaptive mechanism came primarily from intervention studies with the evidence from views studies largely pointing in the opposite direction. This mixed evidence may be explained in part by the differences between the intervention studies which were mainly concerned with primary prevention of HIV – and consequently aiming to reach uninfected immigrants in the 'general' community or among sub-groups who were not HIV-positive – and the views studies – almost half of which were studies made up entirely of immigrants who were HIV-positive. Viewed in this way, the evidence around '*authenticity*' mechanisms points towards its importance in contexts

where primary HIV prevention with immigrants is being carried out and away from its utility in prevention interventions which are seeking to engage people living with HIV. The evidence to support this mechanism from intervention and views studies was largely consistent with how '*authenticity*' had originally been theorised. However, the evidence deepened the insights into how '*authenticity*' is potentially enacted, and resisted, in real-world interventions.

Discussion of 'a*uthenticity*' mechanisms

A central premise of '*authenticity*' mechanisms is that 'closeness' between what Durantini et al call the "agents of change" [53(p. 212)] (those implementing the intervention) and the intervention participants will positively influence the intervention goals in terms of HIV prevention. The strengths of this peer-based approach are widely cited in the literature in HIV prevention with immigrants [53-60]. However, this central premise of 'closeness' in peer-based approaches has rarely been empirically tested to determine if it contributes to the effectiveness of HIV interventions. Where it has, the results have been uneven. Some reviews have found that matching staff demographically (e.g., ethnicity) or behaviourally (e.g., drug use) in HIV prevention to be limited but, nonetheless, important in contributing to behaviour change [53]. However, the premise of 'closeness' also forms a central part of broader theoretical approaches to HIV prevention such as the diffusion model of change which emphasises the benefits of homophily as an important basis for trust and credibility among intervention participants [61]. Others have stressed the effectiveness of using peers in HIV prevention especially in high-risk populations who are often alienated from service providers including sex workers, male transport workers and high school students [62].

An important dilemma that arises in the use of '*authenticity*' in HIV prevention with immigrants is to assess which 'culture' of the participants should be used in 'matching'. For example, should an

intervention with Puerto Rican injecting drug users match intervention staff primarily along the lines of the 'ethnic culture' or the 'drug culture' of participants? [55] In most cases it would appear that for HIV prevention with immigrants 'culture' is often synonymous with 'ethnic culture' [60]. In contexts where the approach is to match staff along 'ethnic culture' lines, the heterogeneity in terms of ethnicity of populations can present another dilemma to the utility of '*authenticity*' mechanisms. It may be hard to define which ethnicities to prioritise in terms of staffing, while staffing an intervention with multi-ethnic staff can contribute significant costs, which may be hard to justify – even if there were compelling evidence that this would generate positive program outcomes. Some interventions overcame this 'multi-ethnic dilemma' through employing staff from a diversity of ethnicities – often as volunteers or casual staff [11, 12, 20] and in some cases having prioritised the ethnicities using routine HIV surveillance data which indicated elevated risks of HIV [11]. Some key limitations in the implementation of this theorised mechanism come from a national program in Switzerland which reported a very high turnover of multi-ethnic staff (mainly due to migration) and a poorly defined professional role of these multi-ethnic bicultural community educators contributing to challenges in the consistent implementation of the intervention [20]. The evidence to refine '*authenticity*' further through gender was contradictory in this review which diverges from studies which suggest that employing ethnically-matched female immigrants to carry out interventions to reach female immigrants contributes to cultural appropriateness [59]. There was very little evidence to support refining this mechanism along sexuality or religious affiliation lines in the studies reviewed.

The evidence around '*framing*' mechanisms

'Framing'

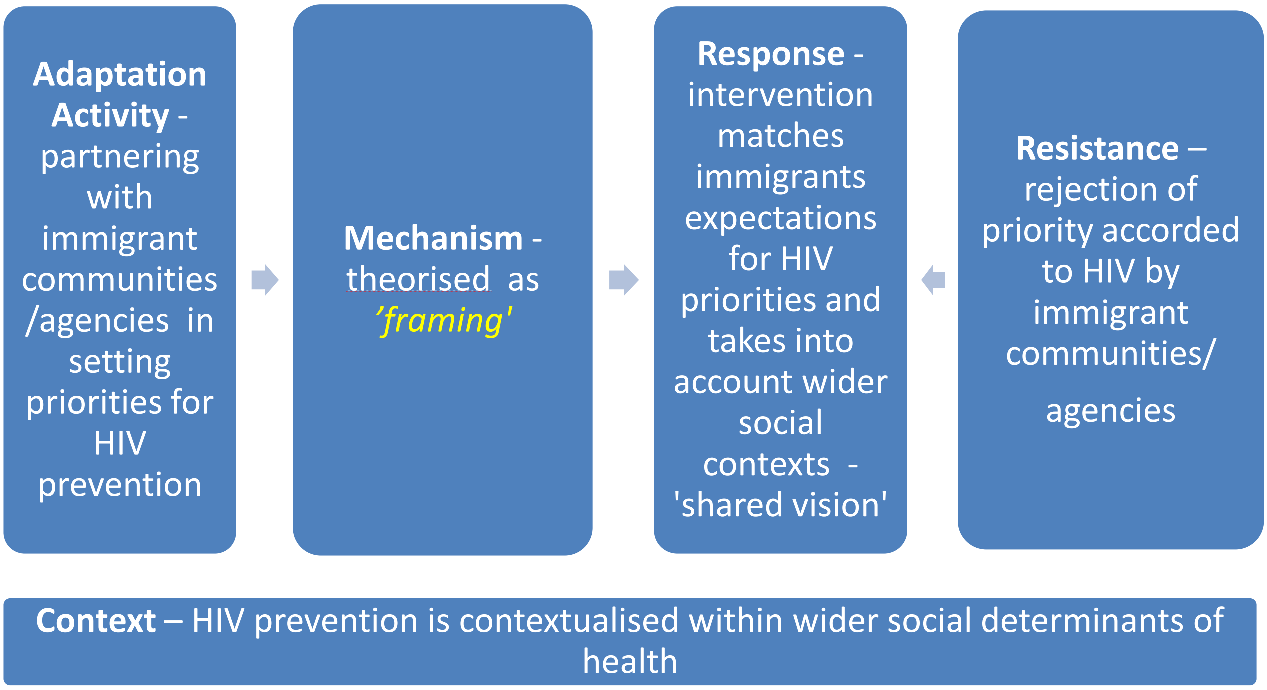

Activity

The evidence around this mechanism was spread across the intervention studies between sound, moderate and partial evidence or no evidence. Eighteen studies reported sound or moderate evidence to support the importance of this mechanism while 23 studies reported partial or no evidence for the adaptation mechanism – a mechanism where the HIV prevention interventions are 'framed' within the wider contexts and priorities of immigrants' lives. This suggests a mixed role for this theorised mechanism in contributing to culturally appropriate behavioural HIV prevention. Among interventions which reported strong or moderate evidence of this mechanism it was found to be operationalised in diverse ways including: interventions being implemented and led by strong coalitions within immigrant communities [4, 5, 7, 12, 17, 18, 20, 25, 26, 63]; framing the HIV

intervention within wider contexts such as racism and homophobia for Latino gay men [6] and gender roles for women at risk of domestic violence [26]; evidence of adjusting the program implementation or program evaluation protocols to address issues which immigrant participants had raised [5, 13, 14, 16, 22]; evidence of consideration of the competing priorities (other than HIV) faced by immigrants in high-income countries [12]; and evidence of the sequencing of priorities in the framing of HIV as a 'general immigrant community' problem in the first phase of a national program in Switzerland to avoid the potentially more difficult issues of homosexuality, injecting drug use and sex work until the partnership with immigrant communities was more established [20]. This intervention from Switzerland illustrates a partnership process with immigrant community institutions which is integral to this theorised mechanism. Similarly, Conner et al [5] describe the development of effective partnerships as a three-way process from "positive initial contacts, experiences (initially low-pressure ones) that develop the partnership, and sufficient time to develop a relationship before a serious challenge arises" (p. 373).

In contrast there was overwhelming evidence to support this mechanism across the views studies with 32 studies reporting sound evidence and a further seven studies reporting moderate evidence indicating that '*framing*' mechanisms were seen as crucial by immigrants themselves in terms of culturally appropriate HIV prevention. There were a range of interrelated themes in the views studies which pointed to broad commonalities across ethnicities, countries of origin, and experiences of migration which impacted on the '*framing*' of interventions and the priority accorded to HIV prevention efforts in immigrants' lives. These could be categorised under two broad themes: migration-related stressors and notions of individual responsibility. Firstly, the interrelated themes of migration-related stressors included migration goals – which generally involved some form of 'escape', or at the very least, a desire for 'betterment' – which were often thwarted in high-income countries through harsh living conditions – particularly in struggles for employment, housing and coping with social isolation [33, 35, 40, 44, 47, 48, 64, 65]. For HIV-positive immigrants the 'escape'

was also paradoxically a form of 'imprisonment' due to the potential difficulties of cross-border travel and the deeper anxiety associated with not being able to return 'home' to live with the support of family as at 'home' they might not be able to afford anti-retroviral treatments or access good HIV medical care [32, 34, 44, 48, 50-52]. Migration-related stressors were further exacerbated among those who were 'undocumented', who were seeking asylum or who did not have permanent residency in the high-income country [33-35, 40, 44, 47, 50, 51, 66]. The struggle to 'get ahead' was also compounded by the need to send remittances to countries of birth to support families [42, 43] and/or pay off people smugglers [33]. For some gay and bisexual male immigrants the 'escape' of migration offered hopes of living without the social stigma of homosexuality in their countries of birth [35, 37, 67] only to be confronted with racism and the impacts of class and poverty in the destination country [38, 39, 68-70]. Pre-migration trauma and post-migration mental health issues [33, 49, 71] were reported as another migration-related stressor in the destination country.

A second broad theme was the importance of notions of collectivism as opposed to individualism for immigrants in framing HIV prevention. This collectivist framing of an immigrant's identity was seen to impact on many areas of HIV prevention [44, 50, 72-76] and pointed to the limits of cognitive and individualistic notions of responsibility for immigrants who live within frames of collective responsibility [73]. Related to the notion of responsibility was the fatalism or lack of individual agency expressed in two studies of HIV-positive immigrants encapsulated in the term *'la suerte'*(or luck) by Latinos in the USA and 'the will of God' by Africans in the UK [41, 44]. In this world view people are powerless [44] and lack any agency against the "immutable, external force" (p. 349) of *'la suerte',* "one either has or does not have good luck" (p. 349).

Response and resistance

The theorised response of immigrants to this mechanism is that the intervention matches the immigrant communities' expectations for priorities in HIV prevention. As with many other

mechanisms, the intervention studies did not directly report the feedback of participants on the theorised '*framing*' of the intervention though the overall effect of many interventions can be inferred, at least in part, to '*framing*' where immigrants themselves influenced the planning, delivery and evaluation of the intervention and were thus able to contextualise HIV prevention within wider social determinants like migration-related stressors, racism and homophobia. Some resistances to the '*framing*' mechanism were reported. These resistances defy easy categorisation and included: resistance to empowerment models of HIV prevention which participants saw as negating their resilience as immigrants [21]; resistance by participants in evaluation processes to HIV-related research [24]; resistance to HIV prevention efforts with regular sexual partners and abstinence messages [2, 22, 77]; and even resistances to the Centers for Disease Control and Prevention categories of HIV transmission risks [25].

Context

In a sense this mechanism is an extension of the '*endorsement*' mechanism (reported below) to deepen the engagement of immigrants in sharing the power to plan, implement and evaluate behavioural HIV prevention interventions. This evidence deviated somewhat from the initial implementation 'chain' for '*framing*'. As originally hypothesised this adaptive mechanism was focused on the evaluation of interventions matching immigrant community expectations for effectiveness (see Additional file 3). The review of evidence suggested that we broaden this adaptation activity to include other aspects of decision making in interventions. This decision making can facilitate framing the intervention within the wider social determinants of health for immigrants and is more responsive to their priorities. The strongest evidence to support this mechanism came from the views studies which may be reflective of the predominance of qualitative research methods in the views studies included in the review – research methods which explicitly attempt to share power in allowing participants to frame insights relevant to HIV. Thus the review of evidence

clarified how '*framing*' was enacted, and potentially resisted, in real-world HIV interventions with immigrants.

Discussion of '*framing*' mechanisms

The mixed evidence to support the importance of this mechanism from intervention and views studies may in part be a reflection of the stage of development that HIV prevention interventions with immigrants have reached. Others have characterised early HIV prevention efforts in high-income countries as being reliant on communication to change individual behaviour followed by a gradual evolution towards community-level interventions and sociopolitical interventions that can enhance and sustain behaviour change – so that the focus is less on communication to 'persuade' and more on environments that 'enable' people to change [78]. In this, '*framing*' mechanisms can be situated within the central movement in public health which sees health promotion as enabling people and communities to take control of their health [79, 80]. In addition, '*framing*' mechanisms are consistent with contemporary thinking on effective behavioural HIV prevention and the need to mobilise communities and build collective action [62, 81] and the need for shared decision-making in HIV prevention interventions with immigrants [58, 82-85].

The views studies suggested a range of wider contexts that could be used to frame interventions under two broad themes of migration-related stressors and framing interventions within notions of collective identity and responsibility. Migration-related stressors could include issues of access to employment, education or housing, and coping with social isolation, which were further exacerbated among those who were not permanent residents. Diaz argues forcefully for understanding these stressors as immediate and current rather than distal stressors in terms of HIV prevention [6]. Similarly, notions of individual agency may be weaker in immigrant communities and HIV prevention may need to be framed within a notion of collective identity and responsibility. For example, in Mao

et al's study of Asian gay men in Sydney, [86] aspects of a gay identity and an Asian identity were seen as being conflicted for the participants with much of the tension around the notion of collectivism, which participants strongly valued as a part of their Asian cultures, and individualism which was perceived to be strongly valued in gay Australian communities. This dovetails with critiques of choice-focused behaviour change models for being based on individualistic cultures and failing to address wider social determinants of health in HIV prevention [81].

The evidence around '*endorsement*' mechanisms

'*Endorsement*'

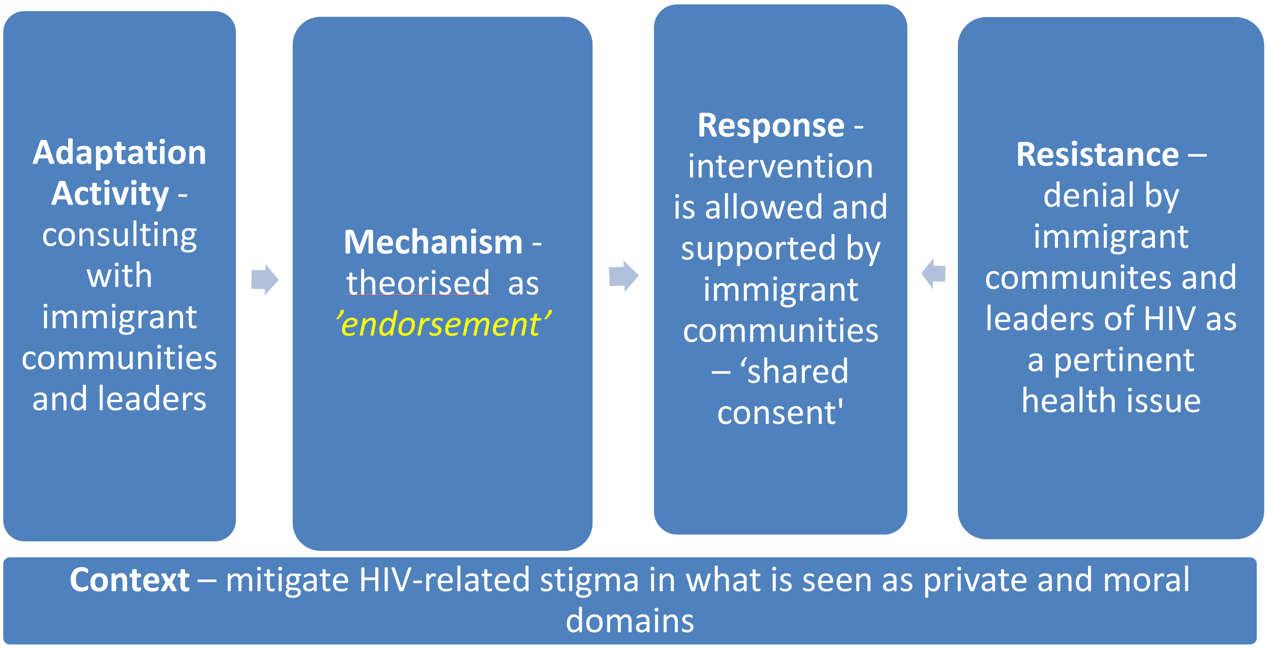

Activity

The evidence for this mechanism from the intervention studies was weak with 24 interventions reporting no evidence and or only partial evidence for this adaptation in culturally appropriate HIV prevention. Against this, 17 interventions reported strong or moderate evidence for '*endorsement*' by immigrant communities and leaders. Among these interventions the mechanism was found to be guided by principles of community participation and operationalised in diverse ways including:

interventions being implemented with the endorsement of community leaders, [7, 9, 12, 17, 18, 20, 26] input by community members in formative evaluations of interventions, [4, 5, 63] or interventions developed by, and extensively focus-tested with, the target group [6, 8, 14, 21, 25] [9] Similarly, there was little evidence to support this mechanism across the views studies with 32 studies reporting no evidence or only partial evidence indicating that '*endorsement*' was seen as less important by immigrants themselves in terms of culturally appropriate behavioural HIV prevention. Only eight studies reported sound or moderate evidence to support this mechanism and this minority of studies stressed the potential for '*endorsement*' mechanisms to mitigate some of the stigma associated with HIV with faith leaders singled out as having a strong role to play in some studies, [33, 41, 47, 49] while other studies stressed the potential role of ethnic community endorsement to promote social capital in ways that were conducive to HIV prevention, [64, 87, 88] and another study highlighted the opportunities for collaboration between HIV and immigrant community agencies to facilitate endorsement of interventions [36].

Response and resistance

The theorised response of immigrants to this mechanism is that they perceive the intervention is 'allowed' and supported by community leaders. In general the interventions did not report on the specific feedback of program participants on this mechanism. In intervention studies that reported no evidence or only partial evidence it was difficult to infer any positive response or resistance to this mechanism. Even in interventions that reported sound or moderate evidence for this mechanism specific feedback was limited. One study reported that the endorsement of the soccer managers, players and captains as a key to an HIV/AIDS soccer tournament gaining acceptance among African-born communities in the UK [18] while another reported that community endorsement was critical to the establishment of a dedicated community-based HIV agency within existing multi-ethnic Asian and Pacific Islander community structures [9]. The sole resistance around

this mechanism was reported in the first implementation phase of a national program in Switzerland where there was widespread denial of HIV as being a pertinent health issue among community leaders [20]. This denial and stigma was widely reported in views studies which were concerned with the sexual transmission of HIV where there were a multiple potential resistances to '*endorsement*' including widespread stigma towards HIV-positive immigrants [37].

Context

This mechanism was not widely found in intervention or views studies but where it was reported it was seen to offer overarching support around a notion of 'shared consent' for the intervention. At an individual and community level HIV can often perceived in terms of morality. The views studies indicated that the endorsement of community leaders – and in particular faith-based leaders – could help mitigate some of the stigma and denial around HIV. In practice, securing the endorsement of faith-based leaders who are often seen as sources of compassion and guardians of morality may be challenging. The partial evidence to support this mechanism from the intervention and views studies was largely consistent with how '*endorsement*' had originally been theorised though the evidence clarified how this mechanism was enacted in real-world HIV interventions with immigrants.

Discussion of '*endorsement*' mechanisms

While this mechanism was not widely found in intervention or views studies it does mirror the literature on effective health promotion with immigrants [56] and the literature on behavioural HIV prevention strategies which draw on influential leaders to endorse change [62]. In practice, securing the endorsement of immigrant community leaders in a HIV context can present some dilemmas. In particular, modern plural nation-states often strive to achieve a "society which is unitary in the public domain but which encourages diversity in what is thought of as private or communal matters" [89(p.208)]. Arguably, the behaviours which drive HIV transmission are intensely private and yet they form part of an important public health issue which can make immigrant community leaders reluctant to endorse what can be seen to be a contentious and stigmatised health issue. Faith-based

institutions may be the least likely of immigrant community institutions to endorse HIV interventions as a recent study of Asian and Pacific Islander institutions in New York City found [90, 91]. The study categorised faith-based institutions as “community sentinels” (p. 244) (the least progressive of the immigrant community institutions) and arts organisations categorised as “paradigm shifters” (p. 244) (the most progressive institutions in terms of willingness to support HIV interventions) [91]. Indeed the key resistance from the wider literature around this mechanism is the denial and reticence among immigrant community leaders to be associated with HIV interventions [90, 91]. Denial of HIV in immigrant communities was identified as a major factor underlying low rates of HIV testing among sub-Saharan African immigrants in Europe in a literature review to guide the development of interventions [92]. The reticence and denial may be in part due to community structures being more aligned with what are perceived to be conservative values and which therefore might not seek to address issues perceived to be on the margins of the ethnic community, such as homophobic attitudes towards gay men [93]. It is therefore important in HIV prevention with immigrants to take a broad view of the term ‘community leader’ as there are marginalised groups, such as gay men, injecting drug users and sex workers, who may not be part of formal immigrant community structures. For example, ‘*endorsement*’ as theorised here can be through consultation with, and the endorsement of, ‘leaders’ drawn from networks of Latino injecting drug users.

References:

1. Raj, A., et al., *Is a general women's health promotion program as effective as an HIV-intensive prevention program in reducing HIV risk among Hispanic women?* Public Health Reports, 2001. **116**(Nov-Dec): p. 599-607.
2. Amaro, H., et al., *Implementation and long-term outcomes of two HIV intervention programs for Latinas.* Health Promotion Practice, 2002. **3**(2): p. 245-54.
3. Chemtov, D., et al., *A culturally specific educational program to reduce the risk of HIV and HBV transmission among Ethiopiam immigrants to Israel: A preliminary report of training veteran immigrants as health educators.* Israeli Journal of Medical Science, 1993. **29**: p. 437-442.
4. Conner, R.F., *Developing and implementing culturally competent evaluation: A discussion of multicultural validity in two HIV prevention programs for Latinos.* New Directions for Evaluation, 2004(102): p. 51-65.

5. Conner, R.F., et al., *The SOLAAR HIV prevention program for gay and bisexual Latino men: USing social marketing to build capacity for service provision and evaluation.* AIDS Education and Prevention, 2005. **17**(4): p. 361-374.
6. Diaz, R.M., *Latino gay men and HIV: Culture, sexuality and risk behavior*. 1998, New York: Routledge.
7. Flaskerud, J.H. and A.M. Nyamathi, *Collaborative inquiry with low-income Latina women.* Journal of Health Care for the Poor and Underserved, 2000. **11**(3): p. 326-342.
8. Hovey, J.D., V. Booker, and L.D. Seligman, *Using theatrical presentations as a means of disseminating knowledge of HIV/AIDS risk factors to migrant farmworkers: an evaluation of the effectiveness of the Informate program.* Journal of Immigrant and Minority Health, 2007. **9**(2): p. 147-56.
9. Loue, S., L. Lloyd, and E. Phoombour, *Organising Asian and Pacific Islanders in an urban community to reduced HIV risk: A case study.* AIDS Education and Prevention, 1996. **8**(5): p. 381-393.
10. Martijn, C., et al., *The effects of AIDS prevention programs by lay health advisors for migrants in The Netherlands.* Patient Education and Counseling, 2004. **53**(2): p. 157-65.
11. McMahon, T., et al., *Evaluation of an ethnic media campaign on patterns of HIV testing among people from culturally and linguistically diverse backgrounds in Australia.* Sexual Health, 2004. **1**(2): p. 91-94.
12. Nakyonyi, M.M., *HIV/AIDS education participation by the African community.* Canadian Journal of Public Health, 1993. **84**(Suppl 1): p. s19-s23.
13. Shtarkshall, R. and V. Soskolne, *Migrant populations and HIV/AIDS. The development and implementation of programmes: theory, methodology and practice*, in *UNAIDS Best Practice - Key Material*. 2000, UNAIDS/UNESCO: Paris.
14. Somerville, G.G., et al., *Adapting the popular opinion leader intervention for Latino young migrant men who have sex with men.* AIDS Education and Prevention, 2000. **18**(Suppl A): p. 137-148.
15. Van Servellen, G., et al., *Program to enhance health literacy and treatment adherence in low-income HIV-infected Latino men and women.* AIDS Patient Care and STDs, 2003. **17**(11): p. 581-594.
16. Wolfers, M., et al., *Using intervention mapping to develop a programme to prevent sexually transmittable infections, including HIV, among heterosexual migrant men.* BMC Public Health, 2007. **7**(141).
17. Worth, H., N. Denholm, and J. Bannister, *HIV/AIDS and the African Refugee Education Program in New Zealand.* AIDS Education and Prevention, 2003. **15**(4): p. 346-356.
18. Marahaj, K., I. Warwick, and G. Whitty, *As assessment of HIV prevention interventions with refugees and asylum seekers*. 1996, HERU, University of London: London.
19. Peragallo, N., et al., *A randomized clinical trial of an HIV-risk-reduction intervention among low-income Latina women.* Nursing Research, 2005. **54**(2): p. 108-118.
20. Haour-Knipe, M., F. Fleury, and F. Dubois-Arber, *HIV/AIDS prevention for migrants and ethnic minorities: three phases of evaluation.* Social Science & Medicine, 1999. **49**(10): p. 1357-1372.
21. Carballo-Dieguez, A., et al., *A randomized controlled trial to test an HIV-prevention intervention for Latino gay and bisexual men: Lessons learned.* AIDS Care, 2005. **17**(3): p. 314-328.
22. Kocken, P., et al., *Effects of peer-led AIDS education aimed at Turkish and Moroccan male immigrants in the Netherlands: A randomised controlled evaluation study.* European Journal of Public Health 2001. **11**(2): p. 153-159.
23. Choi, K.-H., et al., *The efficacy of brief group counseling in HIV risk reduction among homosexual Asian and Pacific Islander men.* AIDS, 1996. **10**(1): p. 81-87.

24. Flaskerud, J.H. and A.M. Nyamathi, *An AIDS education program for Vietnamese women.* New York State Journal of Medicine, 1988. **88**(12): p. 632-637.
25. Flaskerud, J.H., A.M. Nyamathi, and G.C. Uman, *Longitudinal effects of an HIV testing and counseling programme for low-income Latina women.* Ethnicity & Health 1997. **2**(1-2): p. 89-103.
26. Gomez, C.A., M. Hernandez, and B. Faigeles, *Sex in the new world: An empowerment model for HIV prevention in Latina immigrant women.* Health Education & Behavior, 1999. **26**(2): p. 200-212.
27. Kim, Y.-J., N. Peragallo, and B. DeForge, *Predictors of participation in an HIV risk reduction intervention for socially deprived Latino women: A cross sectional cohort study.* International Journal of Nursing Studies 2006. **43**(5): p. 527-534.
28. Martin, M., et al., *The Evaluation of a Latino Community Health Worker HIV Prevention Program.* Hispanic Journal of Behavioral Sciences, 2005. **27**(3): p. 371-384.
29. Mishra, S.I. and R.F. Conner, *Evaluation of an HIV prevention program among Latino farmworkers*, in *AIDS crossing borders: The spread of HIV among migrant Latinos*, S.I. Mishra, R.F. Conner, and R.J. Magana, Editors. 1996, Westfield Press: Boulder, Colorado. p. 157-183.
30. Organista, K.C., et al., *An exploratory study of HIV prevention with Mexican/Latino migrant day laborers.* Journal of HIV/AIDS and Social Services, 2006. **5**(2): p. 89-114.
31. Soskolne, V. and R. Shtarkshall, *Migration and HIV prevention programmes: linking structural factors, culture, and individual behaviour - an Israeli experience.* Social Science and Medicine, 2002. **55**: p. 1297-1307.
32. Korner, H., *'If I had my residency I wouldn't worry': Negotiating migration and HIV in Sydney, Australia.* Ethnicity and Health, 2007. **12**(3): p. 205-225.
33. Chin, J.J., et al. *Looking for a place to call home: A needs assessment of Asian and Pacific Islanders living with HIV/AIDS in New York*. 2007 [cited 2008 3 June]; Available from: www.nyam.org.
34. Kang, E., et al., *The "demon plague" and access to care among Asian undocumented immigrants living with HIV disease in New York City.* Journal of Immigrant Health, 2003. **5**(2): p. 49-58.
35. Korner, H., M. Petrohilos, and D. Madeddu, *Living with HIV and cultural diversity in Sydney*. 2005, National Centre in HIV Social Research: Sydney.
36. Yoshikawa, H., et al., *What front-line CBO staff tell us about culturally anchored theories of behavior change in HIV prevention for Asian/Pacific Islanders.* American Journal of Community Psychology, 2003. **32**(1/2): p. 143-158.
37. Dodds, C., et al. *Outsider status: Stigma and discrimination experienced by gay men and African people with HIV*. 2004 [cited 2008 8 February 2008]; Available from: http://www.sigmaresearch.org.uk/downloads/report04f.pdf.
38. Ayala, G. and R. Diaz, *Racism, poverty and other truths about sex: Race, class and HIV risk among Latino gay men.* Revista Interamericana de Psicologia, 2001. **35**(2): p. 59-77.
39. Wilson, P.A. and H. Yoshikawa, *Experiences of and responses to social discrimination among Asian and Pacific Islander gay men: their relationship to HIV risk.* AIDS Education and Prevention, 2004. **16**(1): p. 68-83.
40. Rhodes, S.D., et al., *Exploring Latino men's HIV risk using community-based participatory research.* American Journal of Health Behavior 2007. **31**(2): p. 146-158.
41. Chinouya, M. and E. O'Keefe, *God will look after us: Africans, HIV and religion in Milton Keynes.* Diversity in Health and Social Care, 2005. **2**(3): p. 177-86.
42. Nemoto, T., et al., *Risk behaviors among Asian women who work at massage parlors in San Francisco: perspectives from masseuses and owners/managers.* AIDS Education and Prevention, 2005. **17**(5): p. 444-56.
43. Shedlin, M.G., C.U. Decena, and D. Oliver-Velez, *Initial acculturation and HIV risk among new Hispanic immigrants.* Journal of the National Medical Association, 2005. **97**(7): p. 32S-37S.

44. Moreno, C.L., *The relationship between culture, gender, structural factors, abuse, trauma, and HIV/AIDS for Latinas.* Qualitative Health Research 2007. **17**: p. 340-352.
45. Flaskerud, J.H., et al., *Sexual practices, attitudes and knowledge related to HIV transmission in Low income Los Angeles Hispanic women.* Journal of Sex Research, 1996. **33**(4): p. 343-353.
46. Dodds, C., *HIV-Related Stigma in England: Experiences of Gay Men and Heterosexual African Migrants Living with HIV.* Journal of Community & Applied Social Psychology 2006. **16**(6): p. 472-480.
47. Anderson, J. and L. Doyal, *Women from Africa living with HIV in London: a descriptive study.* AIDS Care: Psychological & Socio Medical Aspects of AIDS/HIV, 2004. **16**(1): p. 95-105.
48. Shedlin, M.G. and L. Shulman, *Qualitative needs assessment of HIV services among Dominican, Mexican and Central American immigrant populations living in the New York City area.* AIDS Care, 2004. **16**(4): p. 434-445.
49. Doyal, L. and J. Anderson. *My heart is loaded: African women with HIV surviving in London*. 2003 [cited 2008 10 October]; Available from: www.tht.org.uk.
50. Doyal, L. and J. Anderson, *HIV-positive African women surviving in London: report of a qualitative study.* Gender & Development, 2006. **14**(1): p. 95-104.
51. Doyal, L. and J. Anderson, *'My fear is to fall in love again..' How HIV-positive African women survive in London.* Social Science & Medicine, 2005. **60**: p. 1729-1738.
52. Zuniga, M.L., et al., *Exploring care access issues for Mexican-origin Latinos living with HIV in the San Diego/Tijuana border region.* Journal of HIV/AIDS and Social Services, 2006. **5**(2): p. 37-54.
53. Durantini, M.R., et al., *Conceptualizing the influence of social agents of behaviour change: a meta-analysis of the effectiveness of HIV-prevention interventionists for different groups.* Psychological Bulletin, 2006. **132**(2): p. 212-248.
54. Conner, R.F., S.I. Mishra, and R.J. Magana, *HIV prevention policies and programs: Perspectives from researchers, migrant workers and policymakers*, in *AIDS crossing borders: The spread of HIV among migrant Latinos*, S.I. Mishra, R.F. Conner, and R.J. Magana, Editors. 1996, Westfield Press: Boulder, Colorado. p. 186-214.
55. Dushay, R.A., et al., *Lowering HIV risk among ethnic minority drug users: Comparing culturally targeted intervention to a standard intervention.* American Journal of Drug & Alcohol Abuse, 2001. **27**(3): p. 501-524.
56. Grzywacz, J.G., et al., *Serving racial and ethnic populations with health promotion.* American Journal of Health Promotion, 2004. **18**(5): p. 8-12.
57. Idali Torres, M. and G.P. Cernada, *Cultural landscapes and cultural brokers of sexual and reproductive health in US Latino and Latin American populations.* International Quarterly of Community Health Education, 2001/2002. **21**(2): p. 109-132.
58. Peterson, J.L. and A. Carballo-Dieguez, *HIV prevention among African-American and Latino men who have sex with men*, in *Handbook of HIV Prevention*, J.L. Peterson and R. DiClemente, Editors. 2000, Kluwer Academic/Plenum Publishers: New York. p. 217-224.
59. Scott, K.D., A. Gilliam, and K. Branxton, *Culturally competent HIV prevention strategies from women of color in the United States.* Health Care for Women International, 2005. **26**: p. 17-45.
60. Wilson, B.D.M. and R.L. Miller, *Examining strategies for culturally grounded HIV prevention: A review.* Aids Education and Prevention, 2003. **15**(2): p. 184-202.
61. Rogers, E.M., *Diffusion theory: A theoretical approach to promote community-level change*, in *Handbook of HIV Prevention*, J.L. Peterson and R. DiClemente, Editors. 2000, Kluwer Academic/Plenum Publishers: New York. p. 57-66.
62. Coates, T.J., L. Richter, and C. Caceres, *Behavioural strategies to reduced HIV transmission: how to make them work better.* Lancet, 2008. **372**(9639): p. 669-684.

63. Darrow, W.W., et al., *Eliminating disparities in HIV disease:Community mobilization to prevent HIV transmission among black and Hispanic young adults in Broward County, Florida.* Ethnicity and Disease, 2004. **14**(Suppl 1): p. 108-116.
64. Bhattacharya, G., *Social capital and HIV risks among acculturating Asian Indian men in New York City.* AIDS Education and Prevention, 2005. **17**(6): p. 555-567.
65. Dawson, M.T. and S.M. Gifford, *Social change, migration and sexual health: Chilean women in Chile and Australia.* Women & Health, 2003. **38**(4): p. 39-56.
66. Doyle, Y.G., A. Furey, and J. Flowers, *Sick individuals and sick populations: 20 years on.* Journal of Epidemiology and Community Health, 2006. **60**: p. 396-398.
67. Apostolopoulos, Y., et al., *STI/HIV risks for Mexican Migrant Labourers: Exploratory ethnographies.* Journal of the Immigrant and Minority Health, 2006. **8**(3): p. 291-302.
68. Castro-Vazquez, G. and M. Tarui, *'Pueblo chico, infierno grande' - Community support and HIV/AIDS among HIV-positive Latin Americans in Japan.* Ethnicities, 2006. **6**(1): p. 52-73.
69. Diaz, R., G. Ayala, and B.V. Marin, *Latino gay men and HIV: risk behavior as a sign of oppression.* Focus, 2000. **15**(7): p. 1-4.
70. Diaz, R.M. and G. Ayala, *Love, passion and rebellion: ideologies of HIV risk among Latino gay men in the USA.* Culture, Health & Sexuality, 1999. **1**(3): p. 277-293.
71. Steel, J., et al., *Pre-migration trauma and HIV-risk behavior.* Transcultural Psychiatry, 2003. **40**(1): p. 91-108.
72. Chin, D., *HIV-related sexual risk assessment among Asian/Pacific Islander American women.* Social Science & Medicine, 1999. **49**(2): p. 241-278.
73. Gifford, S.M., et al., *Risking for protection: discourses around 'safe sex' among Chilean, Turkish and second-generation Greek women living in Melbourne, Australia.* Ethnicity and Health, 1998. **3**(1/2): p. 95-116.
74. Korner, H., *Late HIV diagnosis of people from culturally and linguistically diverse backgrounds in Sydney: the role of culture and community.* AIDS Care: Psychological & Socio Medical Aspects of AIDS/HIV, 2007. **19**(2): p. 168-78.
75. Korner, H., *Negotiating cultures: disclosure of HIV-positive status among people from minority ethnic communities in Sydney.* Culture, Health & Sexuality, 2007. **9**(2): p. 137-52.
76. Poon, M.K.-L. and P.T.-T. Ho, *A qualitative analysis of cultural and social vulnerabilities to HIV infection among gay, lesbian, and bisexual Asian youth.* Journal of Gay & Lesbian Social Services, 2002. **14**(3): p. 43-78.
77. Hlaing, W.W.M. and W.W. Darrow, *HIV risk reduction among young minority adults in Broward County.* Journal of Health Care for the Poor and Underserved, 2006. **17**(2): p. 159-173.
78. Rivers, K. and P. Aggleton, *HIV prevention in industrialized countries*, in *Handbook of HIV Prevention*, J.L. Peterson and R. DiClemente, Editors. 2000, Kluwer Academic/Plenum Publishers: New York. p. 245-266.
79. Catford, J., *Health promotion: origins, obstacles, and opportunities*, in *Understanding health: a determinants approach.*, H. Keleher and B. Murphy, Editors. 2004, Oxford University Press: South Melbourne, Vic. p. 148-151.
80. Nutbeam, D. and E. Harris, *Theory in a nutshell: A practical guide to health promotion theories.* 2nd ed. 2004, North Ryde, NSW: McGraw Hill.
81. Hankins, C.A. and B.O. De Zalduondo, *Combination prevention: a deeper understanding of effective HIV prevention.* AIDS, 2010. **24**(Suppl 4): p. S70-S80.
82. Marin, B.V., *HIV prevention in the Hispanic community: Sex, culture and empowerment.* Journal of Transcultural Nursing, 2003. **14**(3): p. 186-192.
83. Organista, K.C., H. Carrillo, and G. Ayala, *HIV prevention with Mexican immigrants: Review, critique, and recommendations.* Journal of Acquired Immune Deficiency Syndrome, 2004. **37**(Suppl 4): p. s227-S239.

84. Raj, A., H. Amaro, and E. Reed, *Culturally tailoring HIV/AIDS prevention programs: Why, When, and How*, in *Handbook of Cultural Health Psychology*, S. Kazarian and D. Evans, Editors. 2001. p. 195-239.
85. SenGupta, S., R. Hopson, and M. Thompson-Robinson, *Cultural competence in evaluation: An overview.* New Directions for Evaluation, 2004(102): p. 5-19.
86. Mao, L., J. McCormick, and P. Van de Ven, *Ethnic and gay identification: gay Asian men dealing with the divide.* Culture, Health and Sexuality, 2002. **4**(4): p. 419-430.
87. Shedlin, M.G. and S. Deren, *Cultural factors influencing HIV risk behavior among Dominicans in New York City.* Journal of Ethnicity in Substance Abuse, 2002. **1**(1): p. 71-95.
88. Dawson, M.T. and S.M. Gifford, *Narratives, culture and sexual health: Personal life experiences of Salvadorean and Chilean women living in Melbourne, Australia.* Health, 2001. **5**: p. 403-423.
89. Rex, J., *Multicultural and plural societies*, in *The Ethnicity Reader: Nationalism, multiculturalism and migration*, M. Guibernau and J. Rex, Editors. 1997, Polity Press: Oxford. p. 205-220.
90. Chin, J.J., et al., *Chinese and South Asian religious institutions and HIV prevention in New York City.* Aids Education and Prevention, 2005. **17**(5): p. 484-502.
91. Chin, J.J., et al. (2008) *Paradigm shifters, professionals, and community sentinels: Immigrant community institutions' roles in shaping places and implications for stigmatized public health initiatives*. Health & Place **Volume**, 866-882
92. Prost, A., et al., *Social, behavioural and intervention research among people of Sub-Saharan African origin living with HIV in the UK and Europe: Literature review and recommendations for intervention.* Aids and Behavior, 2008. **12**(2): p. 170-194.
93. Ramirez-Valles, J., *The quest for effective HIV prevention interventions for Latino gay men.* American Journal of Preventive Medicine, 2007. **32**(4S): p. S34-S35.
